# Spatiotemporal Deep Video-Phenomapping Decodes Microvascular Rarefaction in Middle-Aged and Elder Renovascular Hypertension: A Multi-Modal Study Integrating Spatial Transcriptomics and Mitochondrial Pyroptosis

**DOI:** 10.34133/research.1339

**Published:** 2026-07-03

**Authors:** Lingjie Ju, Ri Ji, Hong Meng, Peng Li, Jiaxue Du, Yang Wang, Xuehua Xi, Luzeng Chen, Ying Xiong, Yongjun Li, Pintong Huang, Bo Zhang, Junhong Ren

**Affiliations:** ^1^ Department of Sonography, Beijing Hospital, National Center for Gerontology, Institute of Geriatric Medicine, Chinese Academy of Medical Sciences and Peking Union Medical College, Beijing 100005, China.; ^2^ Department of Ultrasound, Ruijin Hospital, Shanghai Jiao Tong University School of Medicine, Shanghai, China.; ^3^ Department of Echocardiography, State Key Laboratory of Cardiovascular Disease, Fuwai Hospital, National Center for Cardiovascular Diseases, Chinese Academy of Medical Sciences and Peking Union Medical College, Beijing, China.; ^4^ The Key Laboratory of Geriatrics, Beijing Institute of Geriatrics, Institute of Geriatric Medicine, Chinese Academy of Medical Sciences, Beijing Hospital, National Center for Gerontology, Beijing, China.; ^5^ Department of Sonography, Beijing Hospital, National Center for Gerontology, Institute of Geriatric Medicine, Chinese Academy of Medical Sciences, Beijing 100730, China.; ^6^ Department of Ultrasound, China-Japan Friendship Hospital; National Center for Respiratory Medicine; State Key Laboratory of Respiratory Health and Multimorbidity; National Clinical Research Center for Respiratory Diseases; Institute of Respiratory Medicine, Chinese Academy of Medical Sciences; Center of Respiratory Medicine, China-Japan Friendship Hospital, Beijing, China.; ^7^ Department of Ultrasound Medicine, Peking University First Hospital, Beijing 100034, China.; ^8^ Department of Ultrasound Medicine, Civil Aviation General Hospital, Beijing, China.; ^9^ Department of Vascular Surgery, Beijing Hospital, National Center for Gerontology, Institute of Geriatric Medicine, Chinese Academy of Medical Sciences; Chinese Academy of Medical Sciences and Peking Union Medical College, Beijing 100730, China.; ^10^ Department of Ultrasound Medicine, The Second Affiliated Hospital, Zhejiang University School of Medicine, Hangzhou, China.

## Abstract

In middle-aged and older atherosclerotic renal artery stenosis (ARAS), the anatomical severity of stenosis is a poor surrogate for microvascular competence, and the renal benefit of revascularization is unpredictable. We developed Renal-Video-AI, a self-supervised deep learning framework (Video Swin Transformer with VideoMAE pretraining) that extracts spatiotemporal hemodynamic features from contrast-enhanced ultrasound, and applied it to a multi-center Discovery Cohort (*N* = 1,226), an independent External Validation Cohort (*N* = 122), a prospective Multimodal Cohort with paired 10x Visium spatial transcriptomics (*N* = 57), and an aged two-kidney-one-clip (2K1C) murine model. Unsupervised phenomapping identified 3 intrinsic hemodynamic phenotypes—Preserved, Delayed, and Rarefied. The Rarefied phenotype predicted major adverse renal events (MAREs) independently of anatomical stenosis [hazard ratio (HR) 4.82, 95% confidence interval (CI) 3.10 to 6.50; Fine–Gray subdistribution HR (sHR) 5.1], and adding the phenotype to a standard clinical model improved the C-statistic from 0.72 to 0.88. A significant phenotype-by-treatment interaction (*P* < 0.01) showed that stenting reduced events only in the Delayed phenotype (HR 0.52, 95% CI 0.35 to 0.78), not in the Preserved (HR 0.98) or Rarefied (HR 1.05) phenotypes. In absolute terms, stenting reduced the 3-year cumulative incidence of MARE in the Delayed phenotype from 25.4% to 13.2% (absolute risk reduction 12.2%; number needed to treat = 8, 95% CI 6 to 13), with no benefit in the Preserved (8.4% versus 8.0%) or Rarefied (38.6% versus 39.4%) phenotypes. Spatial transcriptomics localized a hypoxia and pyroptosis signature to rarefied tissue, and the aged 2K1C model revealed a mitochondrial reactive oxygen species (ROS)–NLRP3–pyroptosis axis whose pharmacological inhibition (MCC950) restored microvascular perfusion. AI video-phenomapping thus reframes the revascularization decision around microvascular competence rather than anatomy, identifying both therapeutic futility (Rarefied) and a treatable window (Delayed), and nominates NLRP3-driven pyroptosis as a therapeutic target.

## Introduction

Atherosclerotic renal artery stenosis (ARAS) represents a burgeoning epidemic in the aging population, affecting nearly 7% of individuals over 65 years of age [1]. Beyond its role as a secondary cause of hypertension, ARAS is increasingly recognized as a potent driver of “ischemic nephropathy”, a progressive clinical entity associated with a markedly elevated risk of end-stage renal disease (ESRD) and cardiovascular mortality [2]. Despite the intuitive appeal of restoring blood flow to an ischemic kidney, the management of ARAS remains one of the most contentious conundrums in vascular medicine. Landmark randomized controlled trials, notably CORAL [3] and ASTRAL [4], failed to demonstrate a survival or renal benefit of renal artery stenting over optimal medical therapy. These negative results have engendered a pervasive “therapeutic nihilism”, leaving clinicians without clear guidance on how to manage the subset of patients who might truly benefit from revascularization [5].

The failure of these trials underscores a fundamental “anatomy–physiology dissociation”: The degree of large-vessel stenosis is a poor surrogate for tissue-level viability [6]. We postulate that the “missing link” in this equation is microvascular rarefaction—the loss of the peritubular capillary network. In the aging kidney, rarefaction represents a “point of no return”; once the downstream microvasculature is obliterated by fibrosis, restoring upstream macrovascular flow is futile [7]. Consequently, the critical challenge in ARAS is not merely detecting stenosis, but distinguishing between “reversible ischemia” (where the microvasculature is intact and salvageable) and “irreversible rarefaction” [8]. However, noninvasive quantification of renal microvascular health remains an elusive goal. While Doppler ultrasonography [resistive index (RI)] and blood oxygen level-dependent (BOLD) magnetic resonance imaging (MRI) have been proposed, they lack the spatiotemporal resolution to capture the chaotic hemodynamics of capillary loss or are limited by cost and accessibility [9].

Contrast-enhanced ultrasound (CEUS) offers a unique window into renal perfusion dynamics, but the complexity of video data—compounded by respiratory motion artifacts—renders manual quantification unreliable [10]. Emerging advances in computer vision, particularly video vision transformers and self-supervised learning (SSL), now provide the computational power to decode these invisible spatiotemporal features [11]. By treating video as a high-dimensional volume, these deep learning frameworks can extract latent hemodynamic phenotypes that may serve as digital biomarkers of microvascular integrity [12]. Furthermore, the biological driver of rarefaction in the aging kidney remains incompletely understood. While classical theories focus on hypoxia-driven apoptosis, growing evidence suggests that “inflammaging”—the convergence of aging and inflammation—plays a pivotal role [13]. Specifically, aging endothelial cells exhibit mitochondrial dysfunction and ROS accumulation, which may trigger the NLRP3 inflammasome and subsequent pyroptosis (inflammatory cell death) [14]. Unlike immunologically silent apoptosis, pyroptosis releases a storm of proinflammatory cytokines [e.g., interleukin-1β (IL-1β)], potentially accelerating fibrotic remodeling and capillary dropout [15].

Here, we conducted this study integrating clinical artificial intelligence (AI), spatial transcriptomics, and mechanistic interrogation to redefine the landscape of aging ARAS. First, we developed “Renal-Video-AI”, a motion-corrected deep learning framework, to identify unsupervised hemodynamic phenotypes in a large multi-center cohort (*N* = 1,226). Second, we validated these digital phenotypes using paired spatial transcriptomics (*N* = 57), establishing a “digital-biological twin” that links AI-detected perfusion defects to molecular signatures of endothelial loss. Finally, utilizing an aged murine model of 2K1C stenosis, we elucidated the causal role of the mitochondrial–pyroptosis axis in driving microvascular rarefaction. Our findings show a novel precision medicine tool to guide revascularization and identify the NLRP3 pathway as a druggable target for preserving renal function in the elderly.

## Results

### Unsupervised video-phenomapping reveals distinct spatiotemporal hemodynamics in middle-aged and older ARAS

**Fig. 1.**
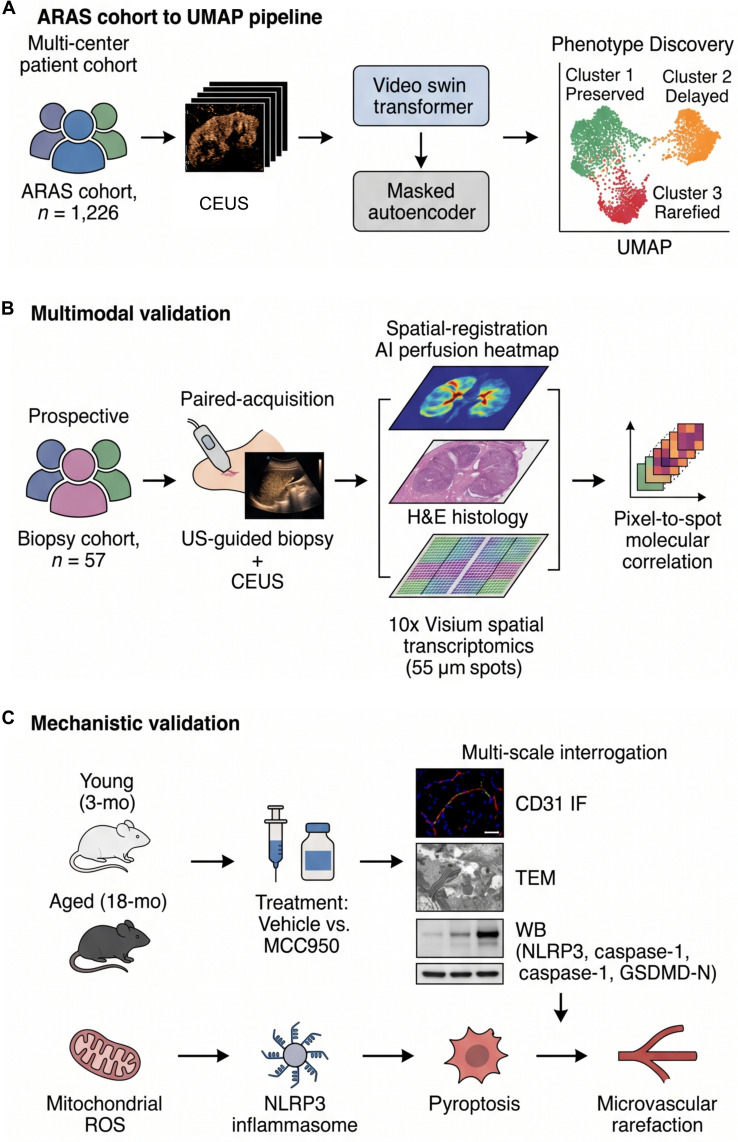
Study design and the 3-tier triangulation framework. (A) Discovery and phenotype discovery: A multi-center cohort of patients with atherosclerotic renal artery stenosis (ARAS; *n* = 1,226) underwent contrast-enhanced ultrasound (CEUS); a Video Swin Transformer with masked-autoencoder pretraining encoded each study, and unsupervised clustering of the UMAP projection defined 3 hemodynamic phenotypes (cluster 1, Preserved; cluster 2, Delayed; cluster 3, Rarefied). (B) Multimodal validation: A prospective biopsy cohort (*n* = 57) underwent paired US-guided biopsy and CEUS, with spatially registered AI perfusion heatmaps, H&E histology, and 10x Visium spatial transcriptomics (55-μm spots) integrated by pixel-to-spot molecular correlation. (C) Mechanistic validation: Young (3-month) and aged (18-month) mice treated with vehicle or MCC950 were interrogated at multiple scales (CD31 immunofluorescence, transmission electron microscopy, Western blot), supporting a mitochondrial ROS → NLRP3 inflammasome → pyroptosis → microvascular rarefaction axis.

To decode the heterogeneity of ischemic renal injury in the middle-aged and older population, we assembled a large, multi-center retrospective Discovery Cohort (*N* = 1,226; mean age 68 years) of patients with ARAS (luminal narrowing ≥ 50%) and developed “Renal-Video-AI”, a self-supervised deep learning framework that extracts latent spatiotemporal hemodynamic features from CEUS without human supervision (Fig. 1).

**Fig. 2.**
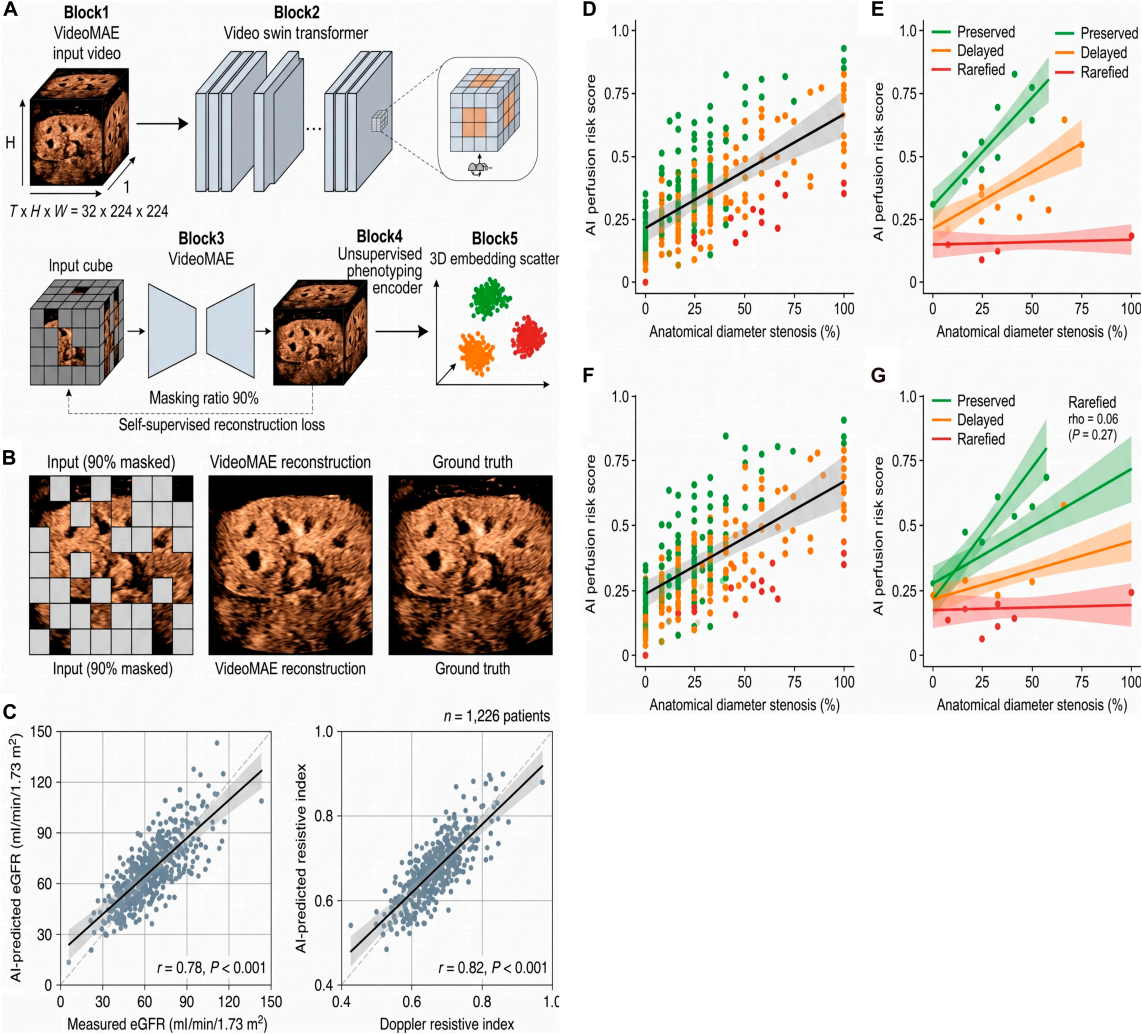
The Renal-Video-AI framework, its physiological validation, and the anatomy–physiology dissociation. (A) Architecture: VideoMAE input cubes (*T* × *H* × *W* = 32 × 224 × 224) are encoded by a Video Swin Transformer; self-supervised reconstruction at 90% masking trains the encoder, and an unsupervised phenotyping head produces a 3D embedding. (B) Representative VideoMAE reconstruction of a 90% masked CEUS frame versus ground truth. (C) AI-derived indices correlate with measured estimated glomerular filtration rate (eGFR) (*r* = 0.78) and the Doppler resistive index (*r* = 0.82; both *P* < 0.001; *n* = 1,226); lines are ordinary-least-squares fits with 95% bands. (D to G) Relationship between anatomical diameter stenosis and the AI perfusion risk score, shown pooled (D and F) and stratified by phenotype (E and G); the association is strong in the Preserved phenotype but abolished in the Rarefied phenotype (Spearman ρ = 0.06, *P* = 0.27), demonstrating dissociation of macrovascular anatomy from microvascular perfusion.

Because respiratory motion is the principal obstacle to renal perfusion quantification, a deformable B-spline registration module stabilized each video sequence, reducing mean interframe displacement from 12.4 ± 3.1 mm to 0.4 ± 0.2 mm (*P* < 0.001, paired Wilcoxon test), with 96.8% of acquisitions passing automated quality control and 3.2% requiring manual reprocessing (Fig. 2A and Fig. S2). A masked-autoencoder (VideoMAE) strategy with a 90% masking ratio then learned to reconstruct microvascular continuity from sparse inputs (Fig. 2B). The resulting latent features correlated strongly with the Doppler RI (*r* = 0.82) and measured estimated glomerular filtration rate (eGFR) (*r* = 0.78; both *P* < 0.001; Fig. 2C), confirming that the model captured biologically meaningful hemodynamic signals rather than imaging artifacts.

### Identification of the “Rarefied” phenotype and the anatomy–physiology dissociation

**Fig. 3.**
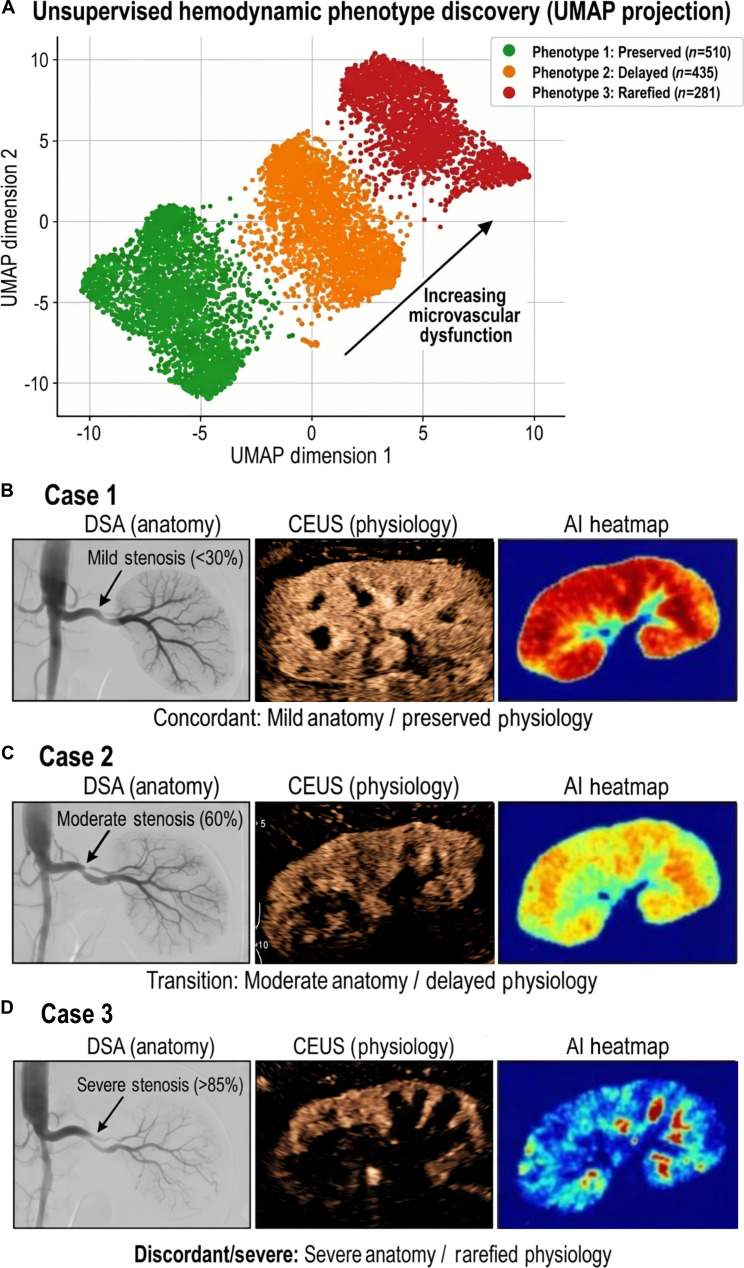
Unsupervised hemodynamic phenotypes and representative anatomy–physiology discordance. (A) UMAP of the discovery cohort colored by phenotype: Preserved (*n* = 510), Delayed (*n* = 435), and Rarefied (*n* = 281); the arrow indicates increasing microvascular dysfunction. (B to D) Representative triads of digital subtraction angiography (DSA) (anatomy), CEUS (physiology), and AI heatmap: (B) case 1, mild stenosis (<30%) with preserved physiology (concordant); (C) case 2, moderate stenosis (60%) with delayed physiology (transitional); (D) case 3, severe stenosis (>85%) with rarefied physiology (discordant).

Uniform manifold approximation and projection (UMAP) with Leiden community detection on the latent space resolved 3 hemodynamic phenotypes intrinsic to middle-aged and older ARAS (Fig. 3A and Fig. S1): cluster 1 (Preserved, *n* = 510, 41.6%; rapid wash-in, high peak enhancement, homogeneous cortical perfusion), cluster 2 (Delayed, *n* = 435, 35.5%; prolonged transit but preserved cortical integrity), and cluster 3 (Rarefied, *n* = 281, 22.9%; cortical perfusion defects, low peak enhancement, slow wash-out). The 3 phenotypes also differed significantly in baseline demographics, renal function, and comorbidity burden, with the Rarefied phenotype showing the oldest age, lowest eGFR, and highest prevalence of diabetes and proteinuria (Table 1; all *P* < 0.001). The phenotypes differed across all morphological, CEUS time–intensity curve (TIC), and AI-derived parameters (Table 2; all *P* < 0.001).

To render the phenotypes interpretable, we decomposed 8 CEUS-derived features by SHapley Additive exPlanations (SHAP) applied to a multinomial gradient-boosted (XGBoost) classifier [classification accuracy 0.892, 95% confidence interval (CI) 0.874 to 0.910; macro-F1 0.876; Fig. S9]. All 8 features differed across phenotypes (*P* < 0.001). Separation was driven most strongly by peak enhancement (mean |SHAP| 0.214; 18.6 versus 14.4 versus 8.8 dB for Preserved, Delayed, and Rarefied), the perfusion heterogeneity index (28%; 12% versus 28% versus 56%), the wash-in rate (0.152; 2.8 versus 1.6 versus 0.7 dB/s), and the time-to-peak (0.131; 12.6 versus 18.8 versus 26.2 s); the cortico-medullary gradient ranked fifth (0.108; 1.84 versus 1.42 versus 0.92). Notably, the Delayed phenotype lay close to the Preserved phenotype on architectural metrics (cortico-medullary gradient, perfusion heterogeneity) but close to the Rarefied phenotype on kinetic metrics (time-to-peak, wash-in rate), consistent with proximal macrovascular obstruction superimposed on a structurally preserved distal microvascular bed. Microvascular rarefaction underlay this gradient. In the biopsy cohort (*n* = 20/22/15), 3 orthogonal measures of capillary density decreased stepwise from Preserved to Rarefied: CD31^+^ peritubular-capillary area (8.6 ± 1.4%/6.2 ± 1.3%/3.1 ± 1.0%), the deconvolution-derived peritubular-capillary endothelial-cell (PTC-EC) fraction (24.8 ± 4.2%/18.4 ± 3.9%/9.6 ± 3.1%), and PECAM1 expression (3.8 ± 0.6/2.9 ± 0.5/1.4 ± 0.4; all *P* < 0.001, one-way analysis of variance (ANOVA) with Tukey post-hoc; Fig. S10). However, anatomical stenosis and functional perfusion were dissociated. Among patients with severe stenosis [>70% on digital subtraction angiography (DSA)], 27.5% were classified as Preserved (robust collateral compensation), whereas 20% of those with moderate stenosis (50% to 70%) were classified as Rarefied (premature microvascular failure; Fig. 3B). Representative case triads (DSA–CEUS–AI heatmap) confirmed that patients with identical stenosis grades could exhibit diametrically opposed microvascular states (Fig. 3C and D).

We tested this dissociation quantitatively (Table S14). Across the cohort, the correlation between stenosis severity and the AI perfusion risk score was modest (Spearman ρ = 0.29, 95% CI 0.24 to 0.34; *P* < 0.001) but strongly phenotype-dependent: ρ = 0.61 in Preserved, 0.42 in Delayed, and 0.06 (95% CI −0.04 to 0.16; *P* = 0.27) in Rarefied. Concordant patterns were observed for the time-to-peak prolongation index (pooled ρ = 0.32) and the wash-in slope (pooled ρ = −0.27), each abolished within the Rarefied phenotype (Fisher *r*-to-*z*, Preserved versus Rarefied Δρ = 0.55, *P* < 0.001), establishing anatomy–physiology dissociation as a defining property of the Rarefied subgroup (Fig. 2D to G).

### The “Rarefied” phenotype predicts renal failure and therapeutic futility

**Fig. 4.**
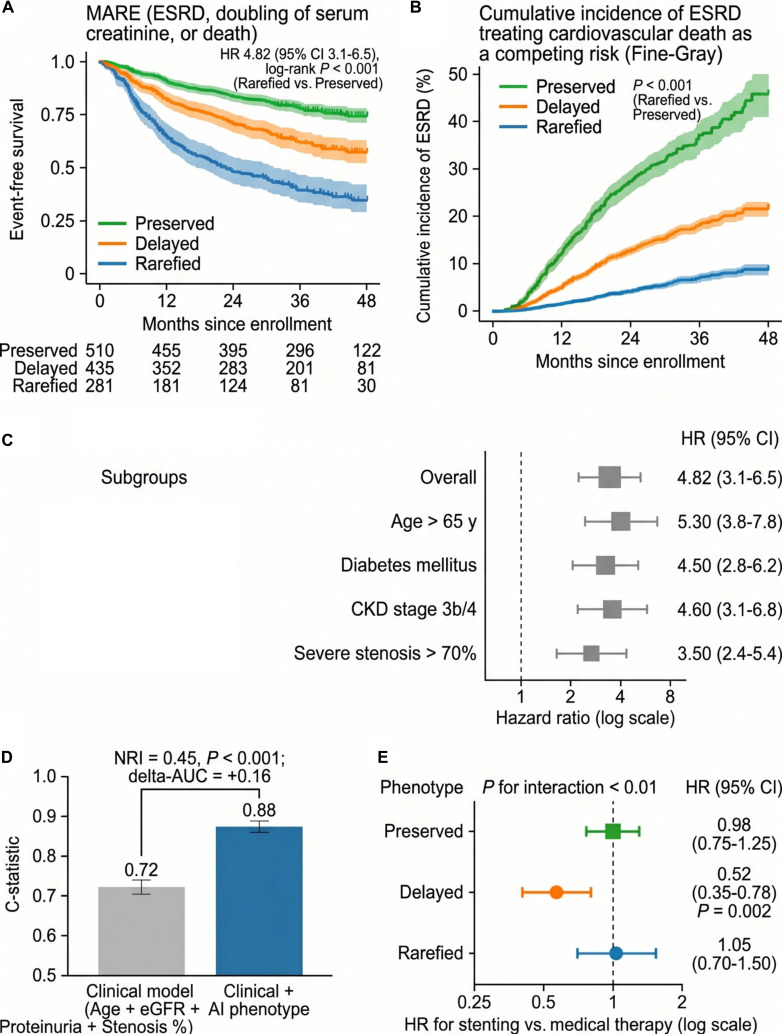
AI phenotypes predict renal prognosis and the response to revascularization. (A) Kaplan–Meier survival free of major adverse renal events [MAREs: end-stage renal disease (ESRD), doubling of serum creatinine, or death from renal causes] with number-at-risk table (Rarefied versus Preserved HR 4.82, 95% CI 3.1 to 6.5; log-rank *P* < 0.001). (B) Cumulative incidence of ESRD with cardiovascular death as a competing risk (Fine–Gray; Rarefied versus Preserved *P* < 0.001). (C) Subgroup forest plot of the prognostic HR (overall 4.82; age >65 years 5.30; diabetes 4.50; CKD stage 3b/4 4.60; severe stenosis >70% 3.50). (D) Adding the AI phenotype to a clinical model (age, eGFR, proteinuria, stenosis %) raises the C-statistic from 0.72 to 0.88 (net reclassification improvement 0.45, *P* < 0.001; ΔAUC +0.16). (E) Phenotype-by-treatment interaction (*P* < 0.01): Stenting versus medical therapy reduces events only in the Delayed phenotype (HR 0.52, 95% CI 0.35 to 0.78; *P* = 0.002), not in the Preserved (0.98) or Rarefied (1.05) phenotypes.

Over a median follow-up of 3.5 years [interquartile range (IQR) 2.1 to 4.8], the phenotypes separated sharply for the composite primary endpoint of major adverse renal events (MAREs: ESRD, sustained doubling of serum creatinine, or death from renal causes), with crude incidences of 9.4%, 21.1%, and 45.6% (48/510, 92/435, and 128/281; Tables 1 and 3A). Kaplan–Meier analysis gave a hazard ratio (HR) of 4.82 (95% CI 3.10 to 6.50; log-rank *P* < 0.001) for Rarefied versus Preserved (Fig. 4A), which remained robust after accounting for the competing risk of cardiovascular death [Fine–Gray subdistribution HR (sHR) 5.10, 95% CI 3.24 to 7.12; Fig. 4B]. The prognostic value was consistent across strata, including age >65 years, diabetes, chronic kidney disease (CKD) stage 3b/4, and severe stenosis (Fig. 4C and Table 3C). Adding the AI phenotype to a standard clinical model (age + eGFR + proteinuria + stenosis %) improved the C-statistic from 0.72 to 0.88 (Δ +0.16, 95% CI 0.10 to 0.22), with a net reclassification improvement of 0.45 (*P* < 0.001) (Fig. 4D and Table 4). Most importantly, the AI phenotype interacted significantly with treatment modality (*P*-interaction < 0.01; Fig. 4E and Table 3D). Stenting conferred no benefit over medical therapy in the Preserved phenotype (HR 0.98, 95% CI 0.75 to 1.25; *P* = 0.85) and was futile in the Rarefied phenotype (HR 1.05, 95% CI 0.70 to 1.50; *P* = 0.72), where revascularization cannot restore an obliterated microvascular bed. In the Delayed phenotype, by contrast, stenting was associated with a significant reduction in events (HR 0.52, 95% CI 0.35 to 0.78; *P* = 0.002), defining a therapeutic window of reversible ischemia. In absolute terms (Aalen–Johansen estimator with cardiovascular death as a competing risk; Table 3E), stenting reduced the 3-year cumulative incidence of MARE in the Delayed phenotype from 25.4% (95% CI 20.7 to 30.1) to 13.2% (95% CI 8.4 to 18.0)—an absolute risk reduction of 12.2% (number needed to treat = 8, 95% CI 6 to 13). No benefit was seen in the Preserved [8.4% versus 8.0%; absolute risk reduction (ARR) 0.4%] or Rarefied (38.6% versus 39.4%; ARR −0.8%) phenotypes, and the CI excluded a clinically meaningful benefit of revascularization in the Rarefied subgroup.

**Table 1.** Baseline characteristics of the Discovery Cohort (*N* = 1,226), stratified by AI-identified hemodynamic phenotype. Mean ± SD or *n* (%). *P* from one-way ANOVA (continuous) or Pearson χ^2^ (categorical). Indented rows are components of the MARE composite (counted once in MARE). eGFR by CKD-EPI. MARE = ESRD + sustained doubling of serum creatinine + death from renal causes.

Variable	Preserved (*n* = 510)	Delayed (*n* = 435)	Rarefied (*n* = 281)	*P*
Demographics				
Age, years	64.8 ± 8.6	68.2 ± 8.8	73.5 ± 7.4	<0.001
Male sex, *n* (%)	296 (58.0)	262 (60.2)	185 (65.8)	0.08
BMI, kg/m^2^	25.2 ± 3.4	25.6 ± 3.6	24.4 ± 3.8	0.35
Current smoker, *n* (%)	138 (27.1)	135 (31.0)	96 (34.2)	0.08
Comorbidities				
Hypertension duration, years	12.2 ± 6.5	14.8 ± 7.2	18.4 ± 8.6	<0.001
Diabetes mellitus, *n* (%)	126 (24.7)	152 (34.9)	124 (44.1)	<0.001
Coronary artery disease, *n* (%)	112 (22.0)	118 (27.1)	96 (34.2)	<0.001
Prior stroke/transient ischemic attack, *n* (%)	44 (8.6)	48 (11.0)	46 (16.4)	0.003
Peripheral artery disease, *n* (%)	62 (12.2)	74 (17.0)	64 (22.8)	<0.001
Atrial fibrillation, *n* (%)	38 (7.5)	42 (9.7)	36 (12.8)	0.04
Renal function				
eGFR, ml/min/1.73m^2^	71.8 ± 18.2	55.4 ± 19.8	35.2 ± 15.6	<0.001
Serum creatinine, μM	96.8 ± 26.4	128.4 ± 40.2	182.6 ± 56.8	<0.001
Proteinuria ≥ 1 g/day, *n* (%)	56 (11.0)	98 (22.5)	108 (38.4)	<0.001
CKD stage 1–2, *n* (%)	308 (60.4)	148 (34.0)	34 (12.1)	<0.001
CKD stage 3a–3b, *n* (%)	170 (33.3)	196 (45.1)	108 (38.4)	
CKD stage 4–5, *n* (%)	32 (6.3)	91 (20.9)	139 (49.5)	
Anatomical stenosis (CTA/DSA)				
Stenosis degree, %	63.8 ± 12.4	66.2 ± 13.8	70.4 ± 14.6	<0.001
Mild (50–60%), *n* (%)	217 (42.5)	130 (29.9)	42 (14.9)	<0.001
Moderate (60–70%), *n* (%)	198 (38.8)	186 (42.8)	108 (38.4)	
Severe (>70%), *n* (%)	95 (18.6)	119 (27.4)	131 (46.6)	
Bilateral RAS, *n* (%)	52 (10.2)	62 (14.3)	58 (20.6)	<0.001
Doppler hemodynamics				
Resistive index	0.63 ± 0.06	0.72 ± 0.07	0.82 ± 0.06	<0.001
Peak systolic velocity, cm/s	242 ± 82	265 ± 88	282 ± 98	0.008
Baseline medications				
ACEi/ARB, *n* (%)	382 (74.9)	338 (77.7)	202 (71.9)	0.18
Statin, *n* (%)	346 (67.8)	310 (71.3)	204 (72.6)	0.29
Antiplatelet, *n* (%)	308 (60.4)	282 (64.8)	194 (69.0)	0.04
Calcium channel blocker, *n* (%)	215 (42.2)	202 (46.4)	138 (49.1)	0.13
Treatment during follow-up				
Renal artery stenting, *n* (%)	142 (27.8)	168 (38.6)	85 (30.2)	0.002
Medical therapy alone, *n* (%)	368 (72.2)	267 (61.4)	196 (69.8)	
Clinical outcomes (median follow-up 3.5 y, IQR 2.1–4.8)				
MARE composite, *n* (%)	48 (9.4)	92 (21.1)	128 (45.6)	<0.001
ESRD, *n* (%)	12 (2.4)	36 (8.3)	64 (22.8)	<0.001
Doubling of creatinine, *n* (%)	28 (5.5)	48 (11.0)	82 (29.2)	<0.001
Death from renal causes, *n* (%)	8 (1.6)	16 (3.7)	22 (7.8)	<0.001
Cardiovascular death, *n* (%)	22 (4.3)	34 (7.8)	38 (13.5)	<0.001
All-cause mortality, *n* (%)	38 (7.5)	58 (13.3)	68 (24.2)	<0.001
Lost to follow-up, *n* (%)	18 (3.5)	16 (3.7)	12 (4.3)	0.86

**Table 2.** Morphological, hemodynamic, and AI-derived parameters across phenotypes. Mean ± SD. *P* from one-way ANOVA; post-hoc by Tukey HSD (orderings at *P* < 0.05 in parentheses). AI perfusion risk score: 0 = preserved, 1 = severe rarefaction. ΔeGFR = change from baseline.

Parameter	Preserved (*n* = 510)	Delayed (*n* = 435)	Rarefied (*n* = 281)	*P* (post hoc)
B-mode renal morphology				
Kidney length, cm	10.8 ± 1.1	10.1 ± 1.3	9.0 ± 1.5	<0.001
Cortical thickness, mm	14.4 ± 2.6	12.2 ± 2.8	9.6 ± 2.4	<0.001
Cortical echogenicity (0–3)	0.8 ± 0.5	1.4 ± 0.6	2.2 ± 0.5	<0.001
Kidney volume, ml	146 ± 30	130 ± 36	106 ± 32	<0.001
CEUS time–intensity curve parameters				
Peak enhancement (PE), dB	18.6 ± 4.0	14.4 ± 3.6	8.8 ± 3.2	<0.001
Time to peak (TTP), s	12.6 ± 3.2	18.8 ± 4.0	26.2 ± 6.4	<0.001
Wash-in rate (WiR), dB/s	2.8 ± 0.7	1.6 ± 0.5	0.7 ± 0.3	<0.001
Mean transit time (MTT), s	22.2 ± 5.4	32.6 ± 6.8	48.4 ± 10.2	<0.001
AUC (area under TIC), dB·s	282 ± 62	216 ± 55	122 ± 46	<0.001
AI-derived video features (Renal-Video-AI)				
AI perfusion risk score (0–1)	0.18 ± 0.11	0.52 ± 0.14	0.84 ± 0.09	<0.001
Perfusion heterogeneity index	0.12 ± 0.04	0.28 ± 0.07	0.56 ± 0.11	<0.001
Perfusion defect area, %	4.2 ± 2.8	18.6 ± 7.8	42.4 ± 11.8	<0.001
Renal function trajectory (ΔeGFR)				
ΔeGFR at 1 y, ml/min/1.73 m^2^	−1.2 ± 4.6	−4.8 ± 5.8	−12.6 ± 8.2	<0.001
ΔeGFR at 3 y, ml/min/1.73 m^2^	−2.6 ± 6.2	−10.4 ± 8.4	−24.8 ± 11.6	<0.001
Annual eGFR slope, ml/min/y	−0.8 ± 2.0	−3.4 ± 2.6	−8.4 ± 3.4	<0.001

**Table 3.** Prognosis across phenotypes—event rates, hazard ratios, subgroups, treatment interaction, and 3-year cumulative incidence. HR from Cox models; sHR from Fine–Gray with cardiovascular death as competing risk. PH assumption verified (Schoenfeld global *P* = 0.42).1

A. Event counts, incidence rates, and hazard ratios (versus Preserved)						
Endpoint	Preserved	Delayed	Rarefied	HR Delayed vs. Pres.	HR Rarefied vs. Pres.	P
MARE composite, n (%)/100 PY	48 (9.4); 2.8	92 (21.1); 6.6	128 (45.6); 17.5	2.48 (1.72–3.58)	4.82 (3.10–6.50)	<0.001
ESRD, n (%)/100 PY	12 (2.4); 0.7	36 (8.3); 2.6	64 (22.8); 8.8	3.82 (1.98–7.38)	9.65 (5.12–18.2)	<0.001
Doubling creatinine, n (%)/100 PY	28 (5.5); 1.6	48 (11.0); 3.5	82 (29.2); 11.2	2.22 (1.40–3.52)	5.86 (3.84–8.94)	<0.001
All-cause mortality, n (%)/100 PY	38 (7.5); 2.2	58 (13.3); 4.2	68 (24.2); 9.3	1.62 (1.08–2.44)	2.85 (1.92–4.24)	<0.001
CV mortality, n (%)/100 PY	22 (4.3); 1.3	34 (7.8); 2.4	38 (13.5); 5.2	1.88 (1.12–3.16)	3.22 (1.96–5.28)	<0.001
B. Adjusted models for MARE (Delayed and Rarefied versus Preserved)						
Model			Delayed vs. Preserved, HR (95% CI)	Rarefied vs. Preserved, HR (95% CI)		
Model 1 (unadjusted)			2.48 (1.72–3.58)	4.82 (3.10–6.50)		
Model 2 (+ age, eGFR, proteinuria, stenosis)			2.12 (1.45–3.10)	4.15 (2.62–5.84)		
Model 3 (+ sex, DM, CAD)			1.98 (1.34–2.92)	3.86 (2.40–5.52)		
Fine–Gray subdistribution (sHR)			2.65 (1.82–3.86)	5.10 (3.24–7.12)		
C. Subgroup analysis—MARE HR for Rarefied versus Preserved (model 2)						
Subgroup			Adjusted HR (95% CI)		P	
Age >65 y			5.30 (3.18–7.78)		<0.001	
Diabetes mellitus			4.50 (2.28–6.20)		<0.001	
CKD stage 3b/4			4.60 (3.12–6.80)		<0.001	
Severe stenosis (>70%)			3.50 (2.20–5.40)		<0.001	
D. Treatment interaction—stenting versus medical therapy (P-interaction < 0.01)						
Phenotype	Stented n	Medical n	HR (95% CI)	P	Interpretation	
Preserved	142	368	0.98 (0.75–1.25)	0.85	No benefit	
Delayed	168	267	0.52 (0.35–0.78)	0.002	Benefit	
Rarefied	85	196	1.05 (0.70–1.50)	0.72	Futile	
E. Three-year cumulative incidence of MARE by arm (NEW panel; merged from the former standalone outcome table)						
Phenotype	Arm	n	3-y cumulative incidence, % (95% CI)	Stenting vs. medical		
Preserved	Medical	368	8.4 (5.6–11.2)	—		
Preserved	Stenting	142	8.0 (3.5–12.5)	ARR 0.4%; HR 0.98 (0.75–1.25)		
Delayed	Medical	267	25.4 (20.7–30.1)	—		
Delayed	Stenting	168	13.2 (8.4–18.0)	ARR 12.2%, NNT 8; HR 0.52 (0.35–0.78)		
Rarefied	Medical	196	38.6 (32.4–44.8)	—		
Rarefied	Stenting	85	39.4 (29.8–49.0)	ARR −0.8%; HR 1.05 (0.70–1.50)		

DM, diabetes; CAD, coronary artery disease

**Table 4.** Stepwise multivariable Cox proportional-hazards models for MARE. HR (95% CI). *P < 0.05, **P < 0.01, ***P < 0.001. PH assumption verified (Schoenfeld global *P* = 0.42); VIF all < 2.5. Stenosis degree is not significant in any model, quantitatively demonstrating the anatomy–physiology dissociation.

Variable	Model 1	Model 2	Model 3	Model 4 (full)
AI video phenotype (ref: Preserved)				
Cluster 2 (Delayed)	2.48 (1.72–3.58)***	2.12 (1.45–3.10)***	1.98 (1.34–2.92)***	1.86 (1.24–2.78)**
Cluster 3 (Rarefied)	4.82 (3.10–6.50)***	4.15 (2.62–5.84)***	3.86 (2.40–5.52)***	3.52 (2.18–5.12)***
Clinical variables				
Age, per 10-y increase	—	1.28 (1.08–1.52)**	1.22 (1.02–1.46)*	1.18 (0.98–1.42)
eGFR, per 10 ml/min decrease	—	1.35 (1.18–1.54)***	1.28 (1.12–1.48)***	1.22 (1.06–1.42)**
Proteinuria ≥ 1 g/day	—	1.82 (1.32–2.52)***	1.68 (1.20–2.34)**	1.54 (1.10–2.18)*
Stenosis, per 10% increase	—	1.12 (0.98–1.28)	1.08 (0.94–1.24)	1.06 (0.92–1.22)
Male sex	—	—	1.15 (0.82–1.62)	1.12 (0.80–1.58)
Diabetes mellitus	—	—	1.42 (1.04–1.94)*	1.35 (0.98–1.86)
Coronary artery disease	—	—	1.28 (0.92–1.78)	1.22 (0.86–1.72)
Doppler parameter				
RI ≥ 0.80 vs. < 0.80	—	—	—	1.45 (1.02–2.06)*
Model performance				
C-statistic (95% CI)	0.74 (0.70–0.78)	0.80 (0.76–0.84)	0.82 (0.78–0.86)	0.88 (0.85–0.91)
Akaike information criterion	2845	2718	2692	2648
ΔC-statistic vs. clinical model ^a^	—	—	—	+0.16 (0.10–0.22)
NRI vs. clinical model	—	—	—	0.45 (0.32–0.58)
IDI vs. clinical model	—	—	—	0.12 (0.08–0.16)

^a^ Clinical model alone (age + eGFR + proteinuria + stenosis, without AI phenotype) C = 0.72 (0.68 to 0.76).

**Table 5.** Diagnostic and predictive performance in the External Validation Cohort (*N* = 122)

Metric	AI phenotype model	Clinical model ^a^	Δ (AI vs. clinical)	*P*
Phenotype distribution (*N* = 122)				
Preserved, *n* (%)	40 (32.8)	—	—	—
Delayed, *n* (%)	52 (42.6)	—	—	—
Rarefied, *n* (%)	30 (24.6)	—	—	—
MARE events, *n* (%)	32 (26.2)	—	—	—
Median follow-up, years (IQR)	3.2 (1.8–4.5)	—	—	—
Discrimination				
C-statistic at 1 y	0.86 (0.78–0.94)	0.74 (0.64–0.84)	+0.12	0.02
C-statistic at 3 y	0.85 (0.78–0.92)	0.72 (0.63–0.81)	+0.13	0.01
C-statistic at 5 y	0.83 (0.76–0.90)	0.70 (0.61–0.79)	+0.13	0.02
NRI (95% CI)	0.42 (0.28–0.56)	—	—	<0.001
IDI (95% CI)	0.10 (0.06–0.14)	—	—	<0.001
Calibration				
Hosmer–Lemeshow *P*	0.68	0.45	—	—
Brier score	0.148	0.195	−0.047	—
Calibration slope	0.92	0.84	—	—
Scanner/vendor independence [AI risk score, median (IQR)]				
GE Logiq E9 (*n* = 34)	0.48 (0.22–0.72)	—	—	—
Canon Aplio i800 (*n* = 28)	0.45 (0.20–0.68)	—	—	—
Siemens Sequoia (*n* = 32)	0.50 (0.24–0.74)	—	—	—
Philips EPIQ 7 (*n* = 28)	0.46 (0.21–0.70)	—	—	—
Kruskal–Wallis *P*	—	—	—	0.72
Prognosis in validation cohort				
MARE: Rarefied vs. Preserved, HR	4.28 (2.12–8.64)	—	—	<0.001
Treatment *P*-interaction	0.03	—	—	—
Cohen’s κ (vs. Discovery)	0.82	—	—	—
Overall phenotype agreement	86.1%	—	—	—

^a^ Clinical model = age + eGFR + proteinuria + stenosis. Vendor independence tested by Kruskal–Wallis across 4 systems. Phenotype concordance assessed by applying the Discovery-trained model to the Validation data.

Because stenting rates differed across phenotypes (27.8%, 38.6%, and 30.2% for Preserved, Delayed, and Rarefied; *P* = 0.002), we performed a within-phenotype 1:1 propensity score-matched sensitivity analysis (caliper 0.2 SD; 354 matched pairs—Preserved 128, Delayed 152, Rarefied 74; all post-match standardized mean differences < 0.10; Table S12 and Fig. S11). Matching preserved the interaction: Stenting reduced MARE only in the Delayed phenotype (matched HR 0.51, 95% CI 0.33 to 0.79), with no benefit in the Preserved (HR 0.94) or Rarefied (HR 1.07) phenotypes (*P*-interaction = 0.01). The concordance across unadjusted (HR 0.98/0.52/1.05), multivariable-adjusted, and matched estimates supports the robustness of the interaction. At the individual level, prespecified 24-month response trajectories were heterogeneous within each phenotype (Table S16). Favorable responses (strong plus modest responder) accounted for 71.4% of stented Delayed patients, 60.6% of Preserved, and 23.6% of Rarefied (Pearson χ^2^ = 89.4, df = 6, *P* < 0.001; Table S13), and progressors were concentrated in the Rarefied phenotype (48.2%). No phenotype was monolithic, however: 23.6% of stented Rarefied patients still achieved a favorable trajectory and 12.7% of stented Preserved patients progressed, supporting AI phenotyping as a probabilistic enrichment strategy rather than a deterministic decision gate.

In a multivariable model of all 395 stented patients (Table S11A; C-statistic 0.84, optimism-corrected 0.82), the independent predictors of 3-year event-free survival were the Delayed [adjusted odds ratio (OR) 3.85 versus Rarefied, 95% CI 2.10 to 7.06] and Preserved (OR 3.42, 1.82 to 6.43) phenotypes, a higher PTC-EC fraction (OR 2.18 per +10%, 1.46 to 3.25), lower baseline proteinuria (OR 0.52 per +1 g/g, 0.38 to 0.71), a shorter CEUS time-to-peak (OR 0.74 per +5 s, 0.61 to 0.90), and a lower AI perfusion risk score (OR 0.69 per +0.1, 0.58 to 0.82). Anatomical stenosis severity (OR 1.05 per +10%; *P* = 0.53), baseline eGFR, age, sex, and diabetes were not retained, quantitatively confirming that recoverability is governed by the downstream microvascular state rather than by upstream stenosis. Within the Delayed phenotype (Table S11B), a higher PTC-EC fraction (OR 2.04 per +10%, 1.22 to 3.41), lower proteinuria (OR 0.57 per +1 g/g, 0.39 to 0.83), a shorter time-to-peak (OR 0.78 per +5 s, 0.62 to 0.98), and a lower AI perfusion risk score (OR 0.66 per +0.1, 0.52 to 0.84) independently predicted event-free survival. Descriptively, favorable responders differed from unfavorable responders by a lower AI perfusion risk score (0.42 versus 0.62), lower proteinuria (median 0.32 versus 1.18 g/day), and higher baseline eGFR (62.4 versus 48.2 ml/min/1.73 m^2^), with a 24-month eGFR change of −2.4% versus −16.8%. A prespecified “cluster 2-stentable” rule—Delayed assignment with proteinuria < 0.5 g/day and a low perfusion heterogeneity index (*n* = 88)—achieved an HR for MARE of 0.31 (95% CI 0.18 to 0.54; *P* < 0.001) with stenting.

Findings generalized to the External Validation Cohort (*N* = 122), in which the phenotype distribution was broadly comparable with the Discovery Cohort (Preserved 32.8%, Delayed 42.6%, Rarefied 24.6%; Table 5), the time-dependent AUC for 3-year MARE prediction was 0.85 (95% CI 0.78 to 0.92), and the AI risk score did not differ across 4 ultrasound vendors (Kruskal–Wallis *P* = 0.72; Fig. S3 and Tables S1 and S2), confirming scanner independence. Phenotype agreement with the Discovery-trained model was high (Cohen’s κ = 0.82; overall agreement 86.1%).

### Spatial transcriptomics validates the molecular basis of rarefaction

**Fig. 5.**
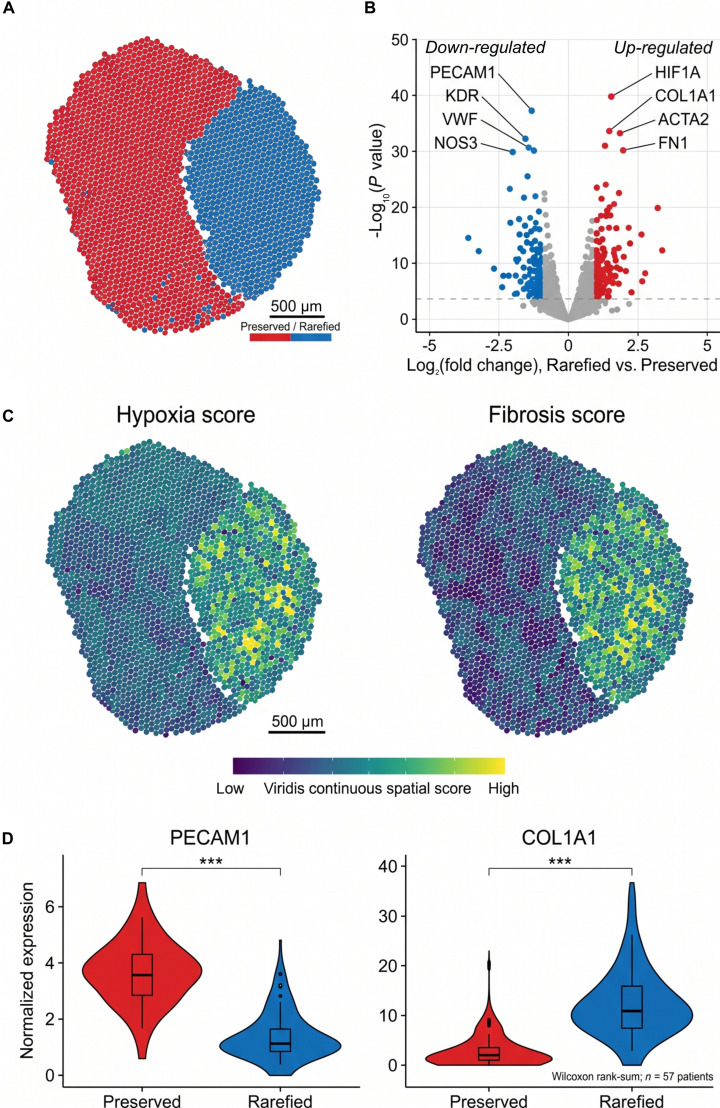
Spatial transcriptomics validates the molecular basis of rarefaction. (A) Representative 10x Visium section partitioned into AI-defined preserved (red) and rarefied (blue) domains; scale bar, 500 μm. (B) Volcano plot (rarefied versus preserved): down-regulated endothelial markers (PECAM1, KDR, VWF, NOS3) and up-regulated hypoxia/fibrosis genes (HIF1A, COL1A1, ACTA2, FN1). (C) Spatial maps of the hypoxia and fibrosis scores (viridis), colocalized to rarefied tissue; scale bar, 500 μm. (D) Reduced PECAM1 and increased COL1A1 in rarefied versus preserved zones (***P < 0.001; Wilcoxon rank-sum; *n* = 57 patients).

To establish the biological ground truth of the phenotypes, paired CEUS and 10x Visium spatial transcriptomics were obtained in the 57-patient Multimodal Validation Cohort (20 Preserved, 22 Delayed, 15 Rarefied; Table S4). Spatial mapping separated preserved (normal perfusion) from rarefied (low perfusion) domains (Fig. 5A). Differential expression in rarefied zones (Fig. 5B) showed down-regulation of endothelial homeostatic markers (PECAM1, KDR, VWF, NOS3) and up-regulation of hypoxia (HIF1A) and profibrotic genes (COL1A1, ACTA2, FN1), with colocalized elevation of hypoxia and fibrosis scores (Fig. 5C and Tables S5 and S6). Regional quantification confirmed reduced PECAM1 and increased COL1A1 in rarefied versus preserved zones (*P* < 0.001, Wilcoxon rank-sum, *n* = 57; Fig. 5D).

**Fig. 6.**
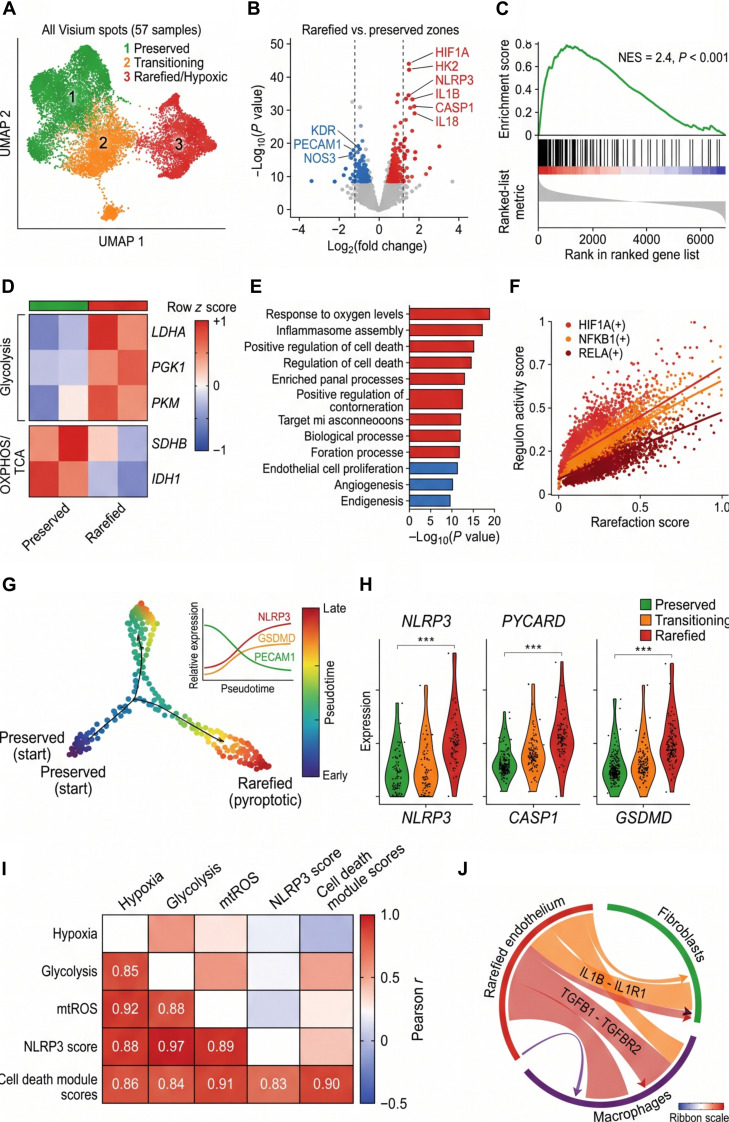
Spatial-transcriptomic dissection of the hypoxia-to-pyroptosis cascade. (A) UMAP of all Visium spots (57 samples): Preserved, Transitioning, and Rarefied/Hypoxic zones. (B) Volcano plot (rarefied versus preserved zones): down-regulated KDR, PECAM1, NOS3 and up-regulated HIF1A, HK2, NLRP3, IL1B, CASP1, IL18. (C) GSEA for Hallmark hypoxia (normalized enrichment score 2.4, *P* < 0.001). (D) Metabolic heatmap showing a glycolytic shift (LDHA, PGK1, PKM up; SDHB, IDH1 down; row *z* score). (E) Gene Ontology enrichment: up-regulated response to oxygen levels, inflammasome assembly and positive regulation of cell death; down-regulated endothelial cell proliferation and angiogenesis. (F) SCENIC regulon activity (HIF1A, NFKB1, RELA) versus rarefaction score. (G) Monocle 3 pseudotime trajectory from preserved to pyroptotic endothelium (inset: NLRP3 and GSDMD rise, PECAM1 falls). (H) Pyroptosis effectors (NLRP3, PYCARD, CASP1, GSDMD) across zones (***P < 0.001). (I) Pearson correlation matrix of hypoxia, glycolysis, mtROS, NLRP3, and cell-death module scores (*r* = 0.83 to 0.97). (J) CellChat chord diagram of signaling from rarefied endothelium to fibroblasts and macrophages (IL1B–IL1R1, TGFB1–TGFBR2).

Higher-resolution analysis delineated a continuum from preserved through transitioning to rarefied/hypoxic microenvironments (Fig. 6A). Rarefied zones down-regulated endothelial genes (KDR, PECAM1, NOS3) and up-regulated hypoxia (HIF1A), glycolysis (HK2), and pyroptosis genes (NLRP3, IL1B, CASP1, IL18; Fig. 6B). The hypoxic signature was validated by Gene Set Enrichment Analysis (GSEA) (Hallmark hypoxia normalized enrichment score 2.4, *P* < 0.001; Fig. 6C) and accompanied by a Warburg-like shift from oxidative phosphorylation (SDHB, IDH1 down) toward glycolysis (LDHA, PGK1, PKM up; Fig. 6D). Gene Ontology enrichment and SCENIC regulon analysis implicated HIF1A and NFKB1/RELA in oxygen sensing, inflammasome assembly, and positive regulation of cell death (Fig. 6E and F and Table S9). Monocle 3 pseudotime traced progression from healthy endothelium to a pyroptotic state (NLRP3 and GSDMD rising as PECAM1 falls; Fig. 6G), corroborated by coordinate up-regulation of NLRP3, PYCARD, CASP1, and GSDMD (*P* < 0.001; Fig. 6H) and by strong correlations (*r* = 0.83 to 0.97) linking hypoxia, glycolysis, mitochondrial ROS, NLRP3, and cell-death module scores (Fig. 6I). CellChat predicted that rarefied endothelium signals to fibroblasts and macrophages via IL1B–IL1R1 and TGFB1–TGFBR2 (Fig. 6J). Reference-based cell-type deconvolution further resolved a peritubular-capillary endothelial subpopulation enriched for senescence markers (CDKN2A, CDKN1A), SASP factors (IL6, CXCL10, MMP3), and NLRP3-pathway transcripts (NLRP3, CASP1, GSDMD), with loss of canonical capillary-identity markers—the inflammaging PTC-EC (iPTC-EC; Fig. S7 and Table S8). The iPTC-EC signature was selectively enriched in rarefied-territory tissue, providing a candidate human cellular correlate of the murine senescent–pyroptotic endothelium described below. Because the percutaneous needle-biopsy cores (median 6.4 mg) precluded droplet-based single-cell or single-nucleus sequencing, endothelial heterogeneity was resolved by deconvolution rather than by direct single-cell capture.

### Aging exacerbates ischemic injury via the mitochondrial–pyroptosis axis

**Fig. 7.**
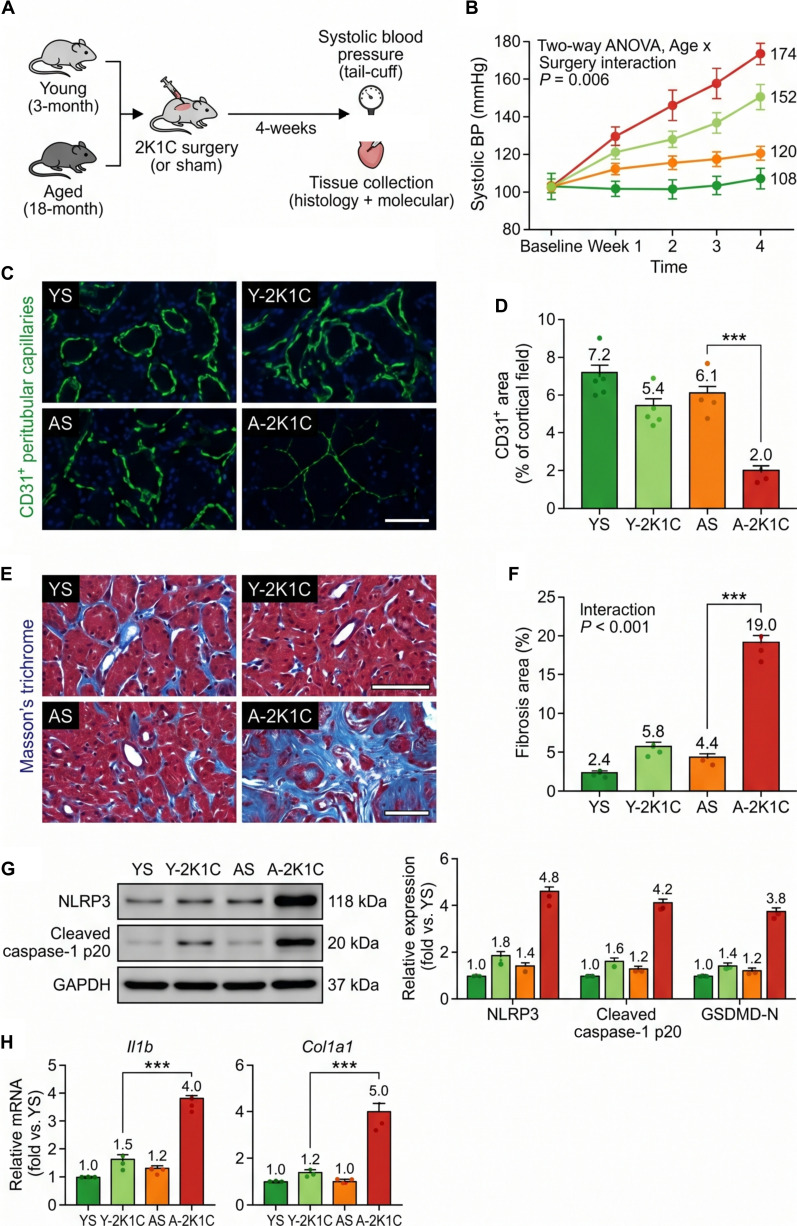
Aging exacerbates ischemic microvascular rarefaction in the 2K1C model. (A) Design: Young (3-month) and aged (18-month) mice underwent 2K1C or sham surgery and were assessed at 4 weeks. (B) Systolic blood pressure time course [week 4: young-sham (YS) 108, aged-sham (AS) 120, young/aged two-kidney-one-clip (Y-2K1C) 152, A-2K1C 174 mmHg]. (C and D) CD31 immunofluorescence and cortical capillary density (YS 7.2, Y-2K1C 5.4, AS 6.4, A-2K1C 2.2% of field; scale bar, 50 μm). (E and F) Masson trichrome and interstitial fibrosis (YS 2.4, Y-2K1C 5.8, AS 4.2, A-2K1C 18.6%; age × surgery *P*-interaction < 0.001; scale bar, 100 μm). (G) Western blot with densitometry for NLRP3, cleaved caspase-1 (p20), and GSDMD-N. (H) Renal Il1b and Col1a1 mRNA. Data are mean ± SEM. ***P < 0.001.

**Fig. 8.**
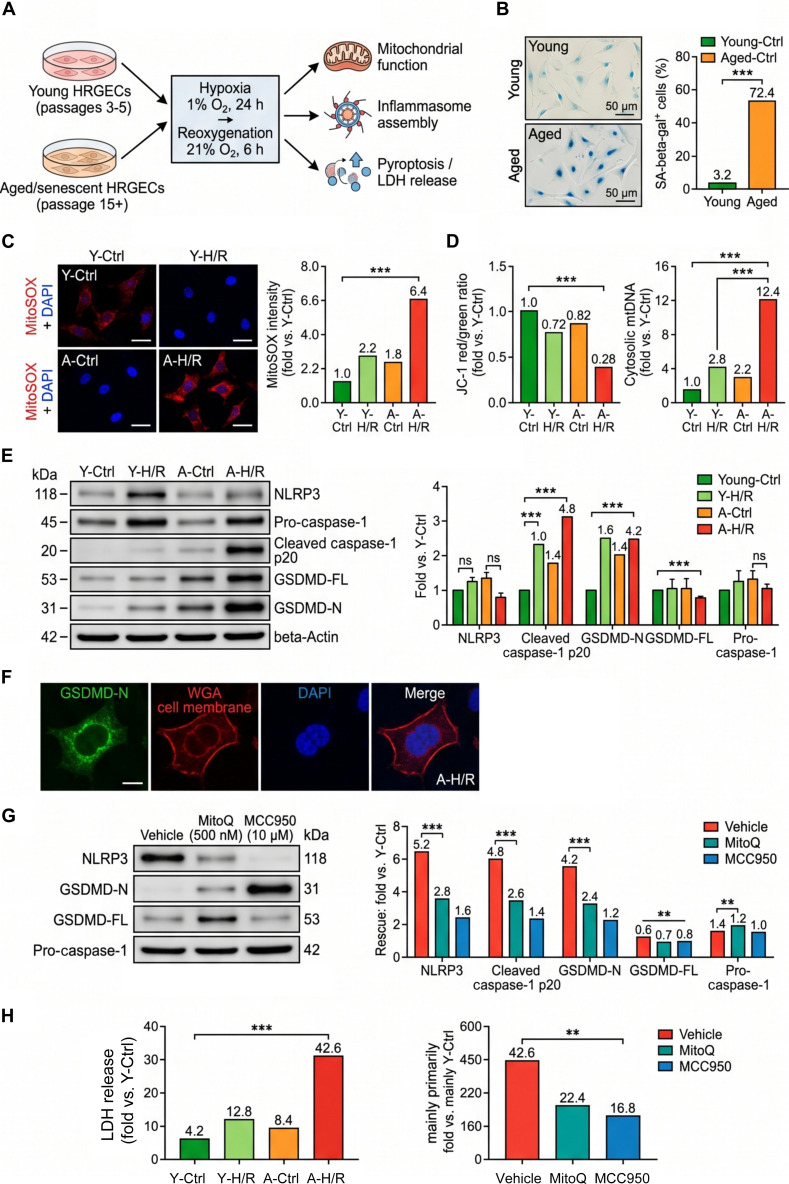
A mitochondrial ROS–NLRP3–pyroptosis axis drives endothelial death in vitro. (A) Design: Young (passages 3 to 5) and aged/senescent (passage 15+) human renal glomerular endothelial cells (HRGECs) subjected to hypoxia (1% O_2_, 24 h) and reoxygenation (21% O_2_, 6 h). (B) SA-β-gal positivity (young 3.2%, aged 72.4%; scale bar, 50 μm). (C) Mitochondrial superoxide (MitoSOX). (D) Mitochondrial membrane potential (JC-1 red/green ratio) and cytosolic mtDNA. (E) Western blot with densitometry for NLRP3, pro- and cleaved caspase-1 (p20), GSDMD-FL, and GSDMD-N. (F) GSDMD-N/WGA/DAPI immunofluorescence showing membrane translocation (A-H/R). (G) Pharmacological rescue by MitoQ (500 nM) and MCC950 (10 μM). (H) Lactate dehydrogenase release across the 4 conditions and after rescue. Data are mean ± SEM. **P < 0.01, ***P < 0.001. H/R, hypoxia/reoxygenation.

To probe causality, we used a two-kidney-one-clip (2K1C) model in young (3-month) and aged (18-month) C57BL/6 mice (Fig. 7A). The 4 groups were balanced before surgery for 11 metabolic, hemodynamic, and renal parameters (within each age stratum all *P* > 0.10, Bonferroni-corrected; the expected aging effect was preserved between strata), so post-surgical differences could not be attributed to baseline metabolic heterogeneity. At 4 weeks, Aged-2K1C kidneys showed severe cortical perfusion defects on CEUS—mimicking the human Rarefied phenotype—whereas Young-2K1C kidneys preserved relative perfusion (Fig. 7B and C), with reduced CD31^+^ capillary density and extensive interstitial fibrosis in the aged group and a significant age × surgery interaction (*P* < 0.001; Fig. 7D to F). Western blotting and quantitative polymerase chain reaction (qPCR) confirmed activation of the pyroptosis cascade (NLRP3, cleaved caspase-1 p20, GSDMD-N, Il1b, Col1a1; Fig. 7G and H). Transmission electron microscopy identified the upstream trigger: endothelial cells in Aged-2K1C kidneys showed mitochondrial swelling, cristolysis, and vacuolization (Figs. 7F and 8A), with excess mitochondrial superoxide (MitoSOX) and collapse of membrane potential (JC-1) accompanied by cytosolic mitochondrial DNA (mtDNA) accumulation (Fig. 8B to D). ASC/NLRP3 specks were present in aged ischemic endothelium, and Western blotting confirmed cleaved caspase-1 (p20) and the pore-forming GSDMD-N fragment, culminating in membrane rupture and lactate dehydrogenase (LDH)/IL-1β release (Fig. 8E to H). Pharmacological rescue in vitro (MitoQ, MCC950) attenuated these effects (Fig. 8G and H), placing mitochondrial ROS upstream of NLRP3.

Three converging events primed the inflammasome in aged post-stenotic endothelium. First, endothelial senescence in the rarefied territory was supported by spatially colocalized induction of CDKN2A (p16, 3.8-fold) and CDKN1A (p21, 2.9-fold), loss of LMNB1 (to 0.4-fold), and a SASP dominated by IL6 (3.2-fold), MMP3 (2.6-fold), and CXCL10 (2.4-fold), with p16^+^ endothelial cells rising from 8.2% to 42.4% (*P* < 0.001; Fig. S14). Second, cell-free mitochondrial DNA was elevated in aged 2K1C kidneys with up-regulation of cGAS–STING and TLR9. Third, PIEZO1 was elevated in rarefied-territory endothelium, and Yoda1-mediated PIEZO1 activation lowered the adenosine triphosphate (ATP) threshold for NLRP3 oligomerization in human renal glomerular endothelial cells (HRGECs) (Figs. S13 and S15). In a 2 × 2 factorial design (young versus replicatively aged endothelium × normoxia versus hypoxia), both aging and hypoxia independently increased mitochondrial ROS, ASC-speck formation, and GSDMD-N, with a significant aging × hypoxia interaction (*P* < 0.001); variance partitioning attributed 47.3% of the variance in ASC-speck formation to aging, 22.6% to hypoxia, and 18.4% to their interaction (residual 11.7%), with concordant partitioning of the Visium dataset (Fig. S16). Thus, inflammaging is the dominant predisposing factor and hypoxia is the principal trigger, acting non-additively on a senescence-primed substrate.

### Pharmacological inhibition of NLRP3 rescues the Rarefied phenotype

**Fig. 9.**
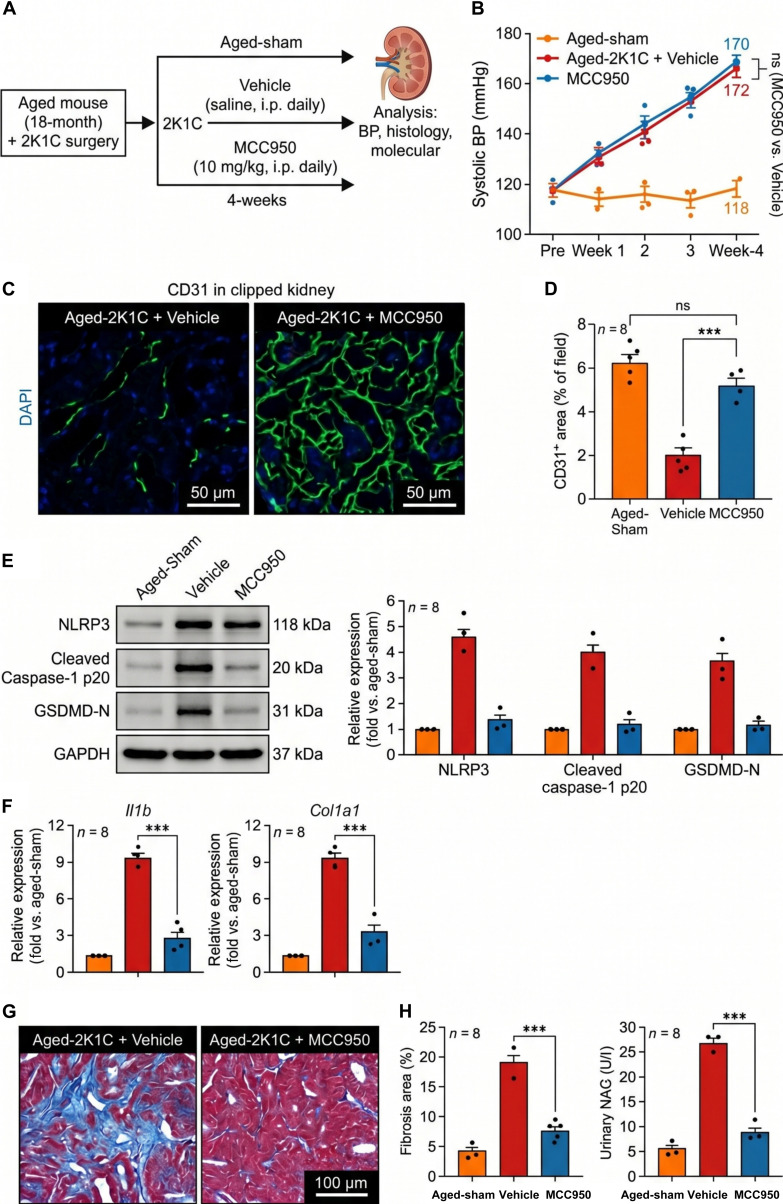
Pharmacological NLRP3 inhibition rescues microvascular rarefaction in vivo. (A) Design: Aged (18-month) 2K1C mice received vehicle or MCC950 (10 mg/kg, intraperitoneally, daily) for 4 weeks, with an aged-sham control. (B) Systolic blood pressure (MCC950 versus vehicle, not significant), indicating a blood pressure-independent effect. (C and D) CD31 immunofluorescence and cortical capillary density (aged-sham 6.1, vehicle 2.0, MCC950 5.0% of field; ***P < 0.001 MCC950 versus vehicle, ns MCC950 versus aged-sham; scale bar as marked). (E) Western blot with densitometry for NLRP3, cleaved caspase-1 (p20), and GSDMD-N. (F) Renal Il1b and Col1a1 mRNA. (G and H) Masson trichrome with interstitial fibrosis (aged-sham 4.4, vehicle 19.0, MCC950 7.8%) and urinary *N*-acetyl-β-d-glucosaminidase (NAG) (5.6, 26.5, 8.8 U/l). Data are mean ± SEM. ***P < 0.001.

To test therapeutic targeting of this axis, Aged-2K1C mice received the selective NLRP3 inhibitor MCC950 (10 mg/kg, intraperitoneally, daily for 4 weeks) with an aged-sham control (Fig. 9A). MCC950 did not lower blood pressure (Fig. 9B), indicating a blood pressure-independent effect, yet restored cortical perfusion toward young-control levels and preserved the CD31^+^ capillary network [cortical capillary density: aged-sham 6.1%, vehicle 2.0%, MCC950 5.0%; *P* < 0.001 versus vehicle, ns (not significant) versus aged-sham; Fig. 9C and D]. It blocked caspase-1 cleavage and GSDMD-N generation, reduced renal Il1b and Col1a1, and attenuated interstitial fibrosis and urinary *N*-acetyl-β-d-glucosaminidase (NAG) (Fig. 9E to H). MitoTEMPO produced a comparable rescue, confirming that mitochondrial ROS acts upstream of NLRP3 in vivo (Fig. S4). The structural benefit was durable: At 12 weeks (4-week treatment plus 8-week washout), CD31^+^ density and serum cystatin C remained improved relative to vehicle (*P* < 0.01), indicating a durable rather than transient effect. Multi-organ histology and serum chemistry [alanine aminotransferase (ALT), aspartate aminotransferase (AST), blood urea nitrogen (BUN), N-terminal pro-B-type natriuretic peptide (NT-proBNP)] revealed no MCC950-related injury to heart, liver, lung, or brain, with only mild on-target reductions in cardiac and hepatic NLRP3 expression (Fig. S12). Early (week 0) initiation prevented progression to the Rarefied phenotype, whereas late (week 4) initiation only attenuated it; peri-procedural MCC950 combined with micro-stenting produced an additive gain in microvascular density, providing a mechanistic rationale for the combination strategy proposed for the “cluster 2-stentable” window-of-opportunity trial.

## Discussion

In this study, we present a multi-modal “triangulation” framework that redefines the understanding of ARAS in the middle-aged and older population. By integrating self-supervised video-based deep learning (“Renal-Video-AI”), spatial transcriptomics, and mechanistic interrogation in senescent animal models, we identified a distinct, imaging-invisible entity: the “Rarefied” phenotype (cluster 3). This phenotype, characterized by cortical perfusion defects, represents a state of irreversible microvascular failure driven by an aging-dependent mitochondrial–pyroptosis axis. Our findings challenge the prevailing anatomy-centric paradigm of renovascular disease, demonstrating that the degree of large-vessel stenosis is a poor surrogate for tissue viability. Instead, we propose that the intersection of aging (the biological substrate) and ischemia (the hemodynamic trigger) aggravates renal injury.

For decades, the management of ARAS has been plagued by a paradox: While opening a stenotic vessel should logically restore perfusion and salvage function, large-scale randomized controlled trials (RCTs) such as CORAL and ASTRAL failed to show a survival benefit for renal artery stenting over medical therapy [3,4,16,17]. Our study provides a mechanistic explanation for these negative trials. We found that approximately one-fourth of patients with anatomically severe stenosis (>70%) exhibited the Preserved phenotype (cluster 1), likely reflecting robust collateralization that renders stenting unnecessary. Conversely, a similar proportion of patients with only moderate stenosis (50% to 70%) exhibited the Rarefied phenotype (cluster 3), in whom the downstream microvasculature was already obliterated by pyroptosis-driven fibrosis. In these patients, restoring macrovascular flow is futile because the capillary bed required to accept the blood flow no longer exists. This dissociation was quantitatively confirmed: The correlation between angiographic stenosis severity and AI-derived perfusion was strong in the Preserved phenotype (Spearman ρ = 0.61) but abolished in the Rarefied phenotype (ρ = 0.06; *P* = 0.27), establishing that once the microvascular bed is lost, luminal status no longer governs perfusion. Crucially, our interaction analysis identified the “Delayed” phenotype (cluster 2) as the specific subgroup that benefits from stenting. In absolute terms, stenting in the Delayed phenotype reduced the 3-year cumulative incidence of MARE from 25.4% to 13.2% (number needed to treat = 8), a benefit that persisted after within-phenotype propensity score matching (matched HR 0.51); a refined “cluster 2-stentable” subphenotype, defined by low proteinuria and a low perfusion heterogeneity index, was further enriched for stenting benefit (HR 0.31). This suggests that the failure of previous RCTs was not due to the inefficacy of stenting per se, but to the dilution of the treatment effect by including patients who were either too healthy (cluster 1) or too sick (cluster 3) to benefit.

Our work represents a departure from, and an advance over, existing approaches to renal assessment across 3 domains: clinical imaging, AI, and mechanistic pathophysiology. Traditionally, the functional assessment of ARAS has relied on Doppler ultrasonography (DUS) and the RI. While an RI > 0.80 has been associated with poor outcomes [18], DUS is highly operator-dependent, limited to large segmental arteries, and unable to resolve microvascular perfusion at the capillary level. More advanced modalities, such as blood oxygen level-dependent (BOLD) MRI and arterial spin labeling (ASL) MRI, have attempted to quantify renal hypoxia and perfusion [19]; for example, Textor and colleagues [20] demonstrated that cortical hypoxia precedes fibrosis in RAS. However, these MRI techniques are expensive, time-consuming, and often contraindicated in advanced renal dysfunction. In contrast, our Renal-Video-AI framework uses CEUS, a bedside modality safe in renal failure. Unlike conventional CEUS studies that rely on simple TIC parameters [21], which reduce the complex hemodynamics of the kidney to a single number, our Video Swin Transformer captures the full spatiotemporal heterogeneity of the wash-in phase. An interpretable SHAP decomposition further showed that the phenotypes are separated along distinct structural, spatial-architectural, and kinetic axes rather than by any single time–intensity parameter. The AI-derived phenotypes achieved higher prognostic accuracy (C-index 0.88) than traditional RI and TIC parameters, establishing Video-AI as a superior digital biomarker of microvascular health.

The application of AI in nephrology has largely been confined to static pathology images or simple 2-dimensional (2D) ultrasound classification (e.g., separating CKD from normal kidneys). Recent studies [22,23] used deep learning to predict eGFR from standard ultrasound images. However, these convolutional neural network (CNN) approaches rely on static morphological features (kidney length, echogenicity) that are late-stage markers of atrophy. Our study is, to our knowledge, the first to employ video-based self-supervised learning (VideoMAE) in renal imaging: By training the model to reconstruct masked video cubes, we force it to learn the dynamics of blood flow—continuity, velocity, and turbulence—rather than static anatomy, allowing detection of functional rarefaction (cluster 3) before anatomical atrophy becomes visible on B-mode ultrasound. Furthermore, unlike most medical-AI studies that lack biological interpretability, we validated the model using a “digital–biological twin” approach with spatial transcriptomics. While previous study pioneered the registration of histology to transcriptomics [24], our study is among the first to register in vivo macroscopic video to ex vivo microscopic gene expression, bridging the pixel-to-gene gap.

The pathophysiology of RAS has been studied extensively in the 2K1C model. Seminal work by Lerman and colleagues [25] established the roles of oxidative stress and endothelial dysfunction in RAS. However, most of these preclinical studies used young animals—a critical translational flaw, as clinical ARAS is predominantly a disease of middle-aged and older adults. Our study addresses this gap with aged (18-month-old) mice, in which we observed a marked phenotypic divergence: Young 2K1C mice maintained relative microvascular density (resembling cluster 1/2), whereas aged 2K1C mice developed severe rarefaction (resembling cluster 3). This aligns with the “inflammaging” theory of Franceschi et al. [26], in which aged tissues have a lower threshold for inflammatory injury. A 2 × 2 factorial dissection in endothelial cells quantitatively supported this premise, attributing 47.3% of the variance in inflammasome activation to aging versus 22.6% to hypoxia (interaction 18.4%), identifying inflammaging as the dominant predisposing factor and hypoxia as the principal trigger. Moreover, whereas previous studies emphasized apoptosis as the mode of cell death in RAS [27], our transcriptomic and Western blot data shift the focus to pyroptosis: Up-regulation of NLRP3, CASP1, and GSDMD was specific to the Rarefied phenotype. This distinction is important because pyroptosis is highly inflammatory (releasing IL-1β), unlike immunologically silent apoptosis, and it explains the marked interstitial fibrosis in cluster 3 as a reactive response to mediators released by pyroptotic endothelial cells—a mechanism underappreciated in apoptosis-centric models of RAS. Mitochondrial ROS was targeted with MitoQ in vitro and MitoTEMPO in vivo, reflecting established pharmacokinetic preferences for each system; both scavenge mitochondrial superoxide, and the concordant results across platforms reinforce the robustness of the mitochondrial–pyroptosis axis.

A key feature of this study is the biological validation of the AI phenotypes. By achieving spatial registration at the section level between the CEUS video planes and 10x Visium spatial transcriptomics, we showed that the “cold spots” identified by the AI are not imaging artifacts but areas of profound biological distinctness. The strong negative correlation between the AI ischemia probability and the endothelial integrity score provides strong evidence that the algorithm performs an in situ, noninvasive assessment of the microvasculature. Cell-type deconvolution further resolved a peritubular-capillary endothelial subpopulation enriched for senescence, SASP, and NLRP3-pathway programs—iPTC-EC—selectively localized to rarefied tissue, providing a candidate human cellular correlate of the murine senescent-pyroptotic endothelium. The transcriptomic signature of the Rarefied regions was characterized by a decoupling of hypoxia and angiogenesis. Hypoxia (high HIF1A) normally triggers angiogenesis (high VEGFA); however, in cluster 3 regions, we observed high HIF1A but paradoxically low vascular endothelial growth factor receptor 2 (VEGFR2) expression alongside high COL1A1. This indicates a state of “senescent angiostasis”, in which the aged endothelium loses its capacity to regenerate in response to ischemia. Instead of sprouting new vessels, these cells activate the NLRP3 inflammasome and undergo pyroptosis. This maladaptive-repair pathway [28] provides the molecular basis for the irreversibility of cluster 3 and explains why revascularization fails to restore function in these patients.

Our mechanistic data underscore the central role of the mitochondrion. In aged endothelial cells, chronic ischemia leads to mitochondrial vacuolization and substantial ROS leakage. Unlike young cells, which buffer this stress via mitophagy, aged cells appear to have defective mitophagy [29], accumulating damaged mitochondria whose ROS leak provides the “Signal 2” for NLRP3 inflammasome assembly. Beyond defective mitophagy, we found that 3 converging signals prime the inflammasome in aged post-stenotic endothelium: endothelial senescence with a SASP signature (p16, p21, IL6, MMP3, CXCL10), tubular cell-free mitochondrial DNA engaging the cGAS–STING and TLR9 sensors [30], and PIEZO1-mediated mechano-priming that lowers the ATP threshold for inflammasome oligomerization [31]. Together, these define the upstream architecture that engages the executive ROS–NLRP3–pyroptosis loop. The identification of this axis opens therapeutic horizons beyond angioplasty. Our intervention data with MCC950 (a selective NLRP3 inhibitor) are particularly promising: In aged 2K1C mice, MCC950 not only reduced inflammation but preserved the microvascular architecture (CD31 density) and improved the AI perfusion score. This benefit was durable—sustained 8 weeks after treatment cessation—and was achieved without detectable injury to heart, liver, lung, or brain, while early initiation was more effective than late initiation, defining a clinically relevant intervention window. These findings indicate that microvascular rarefaction is not an instantaneous event but a progressive, potentially halt-able process of cell death, and they advocate a shift from a “plumbing” approach (fixing the vessel) to a “cytoprotective” approach (preserving the endothelium). Clinical trials of NLRP3 inhibitors [32] should be extended to middle-aged and older ARAS patients, particularly those with the cluster 3 phenotype who are currently considered untreatable.

Four priorities define the path to clinical adoption. First, the AI “traffic-light” phenotyping should be tested in a prospective randomized comparative-effectiveness trial in which the phenotype—not anatomical stenosis—determines revascularization; powered for the Delayed–stratum interaction, this requires approximately 620 patients and 165 events. Second, the continuous AI perfusion risk score is a natural stratification biomarker for NLRP3 inhibitor trials. Third, robust measurement of NLRP3 activation in human biopsies is technically challenging because GSDMD-N is unstable in formalin-fixed tissue, so trial protocols should mandate fresh-frozen cores with orthogonal proximity-ligation validation. Fourth, we propose a window-of-opportunity phase II trial in the “cluster 2-stentable” subphenotype combining stenting with peri-procedural NLRP3 blockade, with change in the AI perfusion risk score at 12 months as the primary endpoint. Several assumptions of our model nonetheless require direct human validation: NLRP3 assembly and GSDMD-N pore formation in human rarefied endothelium are presently inferred from spatial-transcriptomic colocalization rather than measured at the protein level; mitochondrial ROS has not been directly quantified in human renal endothelium; and the responsiveness of the human Rarefied phenotype to NLRP3 blockade remains to be demonstrated.

### Strengths and limitations

The primary strength of this study is its triangulation design, which grounds the clinical phenotypes in molecular reality and verifies them with causal animal experiments. The large multi-center discovery cohort (*N* = 1,226) and the external validation cohort (*N* = 122) with diverse ultrasound systems support the robustness and generalizability of the Video-AI model, and the stabilization of respiratory motion addresses a major technical barrier in renal ultrasound quantification. Several limitations remain. First, the discovery cohort was retrospective, introducing potential selection bias; although the model was validated prospectively and externally, a randomized controlled trial using the AI-based traffic-light system to guide stenting is the necessary next step. Second, the CEUS–Visium integration was performed at the section level; a formal landmark-based, compartment-stratified target-registration-error analysis between CEUS planes and individual Visium spots was not undertaken. Third, droplet-based single-cell or single-nucleus RNA sequencing was not performed because of the limited tissue mass of the needle-biopsy cores; endothelial heterogeneity was resolved by reference-based deconvolution, which identified the iPTC-EC subpopulation, and single-cell profiling of an independent surgical cohort is planned to validate it at single-cell resolution. Fourth, our animal model used 2K1C, which mimics RAS but does not fully capture human atherosclerosis (e.g., cholesterol emboli) [33]; future studies should employ ApoE-deficient atherosclerotic mice. Fifth, the in vitro senescence model used replicative senescence, which may not fully recapitulate chronological aging, although the consistency between the in vitro (P15+ HRGECs) and in vivo (18-month-old mice) findings supports its translational relevance. Finally, the human Renal-Video-AI framework was not applied directly to the murine micro-CEUS data because of differences in imaging frequency and species-specific anatomy; the murine perfusion analysis relied on conventional TIC parameters, serving as independent histological validation rather than a direct cross-species AI transfer.

### Conclusion

In conclusion, this study establishes deep video-phenomapping as a powerful tool for the management of middle-aged and older renovascular hypertension. We identified a distinct Rarefied phenotype that signifies the limit of renal adaptability—a point of no return driven by mitochondrial dysfunction and endothelial pyroptosis. By looking beyond anatomical stenosis to the health of the downstream microcirculation, we can resolve the “stenting paradox”, sparing cluster 1 and cluster 3 patients from unnecessary procedures while offering effective intervention to cluster 2. Our definition of the mitochondrial–pyroptosis axis provides a roadmap for vascular-preserving therapies aimed at preserving renal function in a middle-aged and older population.

## Methods

### Study design and ethical oversight

This multi-center, translational investigation employed a “triangulation” study design to deconstruct the heterogeneity of ischemic renal disease in the middle-aged and older population. The study integrates 3 layers of evidence: (a) macroscale phenotyping via unsupervised deep learning in large retrospective and external-validation cohorts; (b) meso-scale validation via prospective spatial transcriptomics to establish the molecular ground truth of imaging signals; and (c) micro-scale mechanism via an aging murine model to elucidate the mitochondrial–pyroptosis axis.

The protocol was approved by the Institutional Review Boards of Beijing Hospital (approval no. 2025BJYYEC-KY371-02) and all participating satellite centers. Development and reporting of the deep learning model adhere to the STARD-AI and TRIPOD-AI guidelines (Table S10).

### Clinical cohorts and eligibility criteria

#### Discovery Cohort (retrospective, *N* = 1,226)

We curated a multi-center dataset of patients evaluated for renovascular hypertension between January 2015 and December 2024 across 7 tertiary centers. To isolate the phenotype of middle-aged and older ARAS, patients were required to have: (a) age ≥ 45 years; (b) anatomically severe RAS [≥50% luminal narrowing on computed tomography angiography (CTA) or DSA]; and (c) raw, uncompressed CEUS digital imaging and communications in medicine (DICOM) video loops (≥60 s) covering complete wash-in and wash-out. Exclusions: non-atherosclerotic etiologies (fibromuscular dysplasia, arteritis), biopsy-confirmed primary glomerulonephritis, complete (100%) occlusion, acute kidney injury or hemodynamic instability, and poor acoustic windows [e.g., body mass index (BMI) > 35 kg/m^2^]. The 45-year threshold demarcates the population at biological risk of atherosclerotic vascular aging rather than a geriatric definition, consistent with the lower age boundaries used in major renal artery stenosis trials (CORAL, ASTRAL); the cohort is accordingly described as “middle-aged and older”.

#### External Validation Cohort (independent, *N* = 122)

To assess generalizability and scanner independence, we recruited an independent cohort from 6 geographically distinct centers acquired on 4 scanner platforms from different manufacturers—GE Healthcare (Logiq E9), Canon/Toshiba (Aplio i800), Siemens (Acuson Sequoia), and Philips (EPIQ 7)—distinct from the Mindray systems used in the Discovery Cohort (GE 34, Canon/Toshiba 28, Siemens 32, Philips 28; Table 5). Inclusion and exclusion criteria were identical to the Discovery Cohort (Tables S1 to S3).

#### Multimodal Validation Cohort (prospective, *N* = 57)

From June 2023 to January 2025, we prospectively enrolled patients with confirmed ARAS (≥50%) scheduled for renal biopsy or revascularization at Beijing Hospital. CEUS and ultrasound-guided core-needle biopsy were performed within a <48-h window to minimize temporal confounding. Written informed consent was obtained for 10x Visium sequencing of tissue cores; the final analytic cohort comprised 57 patients (20 Preserved, 22 Delayed, 15 Rarefied).

### The “Renal-Video-AI” engineering framework

#### Spatiotemporal motion correction

Respiratory motion induces nonrigid renal deformation (>2 cm displacement) that confounds pixel-level perfusion analysis. We engineered a deformable B-spline registration network using a multi-resolution free-form deformation model. The frame of peak cortical enhancement served as the fixed reference; for every moving frame, a dense displacement mesh minimized the negative mutual-information cost, aligning the cortex pixel-by-pixel across the sequence to generate a stabilized “Video Cube” (*T* × *H* × *W*) in which temporal intensity changes reflect hemodynamics. Registration reduced mean interframe displacement from 12.4 ± 3.1 mm to 0.4 ± 0.2 mm (*P* < 0.001, paired Wilcoxon test); quality was monitored by an automated residual-displacement threshold, with 96.8% of acquisitions passing automated quality control and 3.2% undergoing manual reprocessing (Fig. S2).

#### Network architecture: Video Swin Transformer

We used the Video Swin Transformer (Swin-B) as the feature backbone. Its 3D shifted-window attention captures the high-frequency “flicker” of microbubbles entering the capillary bed (wash-in kinetics) and the cortico-medullary perfusion gradient required to identify cortical rarefaction while ignoring medullary sparing.

#### Self-supervised pretraining (VideoMAE)

A masked-autoencoder strategy with a 90% masking ratio trained the network to reconstruct missing spatiotemporal tubelets from the visible 10%, forcing it to learn high-level representations of hemodynamic continuity and to flag perfusion defects (rarefaction) as high-entropy anomalies that cannot be logically reconstructed.

#### Unsupervised phenotype discovery

We extracted 1,024D latent feature vectors from the encoder, reduced dimensionality by UMAP, and applied Leiden community detection. The optimal cluster number (*k* = 3) was determined by maximizing the silhouette coefficient and verified by consensus-clustering stability analysis (B = 1,000 bootstraps; Fig. S1).

#### Interpretable feature decomposition

To render the unsupervised phenotypes interpretable, 8 a priori CEUS time–intensity and spatial features were extracted for each patient—peak enhancement, the perfusion heterogeneity index, the wash-in rate, time-to-peak, the cortico-medullary gradient, the wash-out rate, microvascular density, and mean transit time—organized along 3 conceptual axes (structural rarefaction, spatial-architectural integrity, and kinetic disorganization). A multinomial gradient-boosted classifier (XGBoost, v1.7) was trained to predict cluster assignment from these features, and SHAP (v0.43) quantified the mean absolute contribution (|SHAP|) of each feature. Multicollinearity was assessed by variance inflation factors (all < 3.0), and classification accuracy and macro-averaged F1 were reported with bootstrap CIs (Fig. S9).

### Spatial transcriptomics (the “digital–biological twin”)

#### The “sonography-to-sequencing” registration pipeline

During biopsy, the needle trajectory was digitally recorded relative to the transducer, and the CEUS video plane corresponding to the biopsy track was extracted to generate an AI attention (class-activation) map. The hematoxylin and eosin (H&E) image of the biopsy core was spatially registered to the attention map using anatomical landmarks (renal capsule, arcuate arteries) via an affine transformation. This integration was performed at the section level by mapping spots to the registered cortical perfusion field; a formal landmark-based, compartment-stratified, target-registration-error analysis between CEUS planes and individual Visium spots was not undertaken and is acknowledged as a limitation.

#### Bioinformatics and deconvolution

Raw data were processed with Space Ranger (v2.1). Because each 55-μm Visium spot contains multiple cells, spatial deconvolution was performed with RCTD (robust cell-type decomposition) to resolve per-spot composition (endothelial, podocyte, tubular, fibroblast); this image-to-molecular mapping approach is consistent with recent spatial-transcriptomic deconvolution frameworks [34]. Module scores were computed for endothelial integrity (PECAM1, CDH5, KDR, vWF), mitochondrial dysfunction (PPARGC1A, NRF1, TFAM), and pyroptosis (NLRP3, CASP1, GSDMD, IL1B, IL18), and pixel-wise Pearson correlations were computed between the AI risk score and these scores across spatially matched spots.

Endothelial spots were further subclassified by module scoring for senescence (CDKN2A, CDKN1A, with loss of LMNB1), the senescence-associated secretory phenotype (IL6, MMP3, CXCL10), and the NLRP3 pathway (NLRP3, CASP1, GSDMD); a peritubular-capillary endothelial subpopulation coexpressing these programs was designated the iPTC-EC (Table S8). Because the tissue mass of the percutaneous needle-biopsy cores (median 6.4 mg) was insufficient for droplet-based single-cell or single-nucleus RNA sequencing, endothelial heterogeneity was resolved by reference-based deconvolution rather than by direct single-cell capture.

### Aging mechanism investigation (murine models)

All animal experiments were approved by the Institutional Animal Care and Use Committee (IACUC) of the Second Affiliated Hospital of Zhejiang University School of Medicine (approval no. ZJCLA-IACUC-20011469) and performed per the National Institutes of Health (NIH) Guide and the ARRIVE 2.0 guidelines.

#### Animal model (aged 2K1C)

Male C57BL/6 mice aged 18 months (senescent; equivalent to humans 56 to 69 years) and 3 months (young control) were used. The 2K1C model was induced with a U-shaped silver clip (0.12 mm internal diameter) on the right renal artery (60% to 70% stenosis) following an established murine renovascular-hypertension protocol [35]; sham-operated mice served as controls. Aged 2K1C mice were randomized to vehicle (saline), MitoTEMPO (0.7 mg/kg/day, intraperitoneally), or MCC950 (10 mg/kg, intraperitoneally, daily) using computer-generated random numbers, with investigators blinded to allocation.

One week before surgical randomization, all animals underwent baseline characterization of body weight, fasting blood glucose, hemoglobin A1c (HbA1c), tail-cuff systolic and diastolic blood pressure, plasma lipids (total cholesterol, triglycerides, high-density lipoprotein, low-density lipoprotein), serum creatinine, and urinary albumin/creatinine ratio (11 parameters; Table S15). Prespecified exclusion criteria (fasting glucose ≥ 11.1 mM; body weight beyond 2 SD of the strain-and-age mean) were applied a priori; within-age-stratum comparisons used 2-tailed *t* tests with Bonferroni correction.

#### In vivo micro-CEUS perfusion imaging

At 4 weeks post-surgery, renal perfusion was quantified on a high-frequency system (VisualSonics Vevo 3100, 40 MHz). Bolus MicroMarker injections were recorded, and wash-in/wash-out kinetics were analyzed to validate the rarefaction phenotype in vivo.

#### Mitochondrial and molecular analysis

Transmission electron microscopy quantified endothelial mitochondrial damage (swelling, cristolysis, vacuolization) at 15,000×. Immunofluorescence for CD31 quantified microvascular density, with terminal deoxynucleotidyl transferase–mediated deoxyuridine triphosphate nick end labeling (TUNEL)/caspase-1 costaining for pyroptotic endothelium. Cortical lysates were probed by Western blotting for cleaved caspase-1 (p20), NLRP3, and GSDMD-N; the NLRP3–caspase-1–GSDMD pyroptosis cascade was interrogated as a candidate driver of inflammatory microvascular loss [36].

#### Durability, multi-organ safety, timing, and combination studies of MCC950

Durability was assessed in a subset of aged 2K1C mice that received MCC950 for 4 weeks followed by an 8-week treatment-free interval, with CEUS perfusion, CD31 density, and serum cystatin C measured at 12 weeks. Multi-organ safety was evaluated by H&E with semiquantitative organ-injury scoring of heart, liver, lung, and cerebral cortex, CD68 and Ly6G immunostaining, serum ALT, AST, BUN, and NT-proBNP, and tissue NLRP3 expression (Fig. S12). Intervention timing was tested by comparing early (initiated at clipping) versus late (initiated at week 4) MCC950 administration. The interaction with revascularization was tested by combining peri-procedural MCC950 with micro-stenting of the clipped artery (*n* = 6 per group), with CD31 density as the endpoint.

#### Upstream priming analyses

Endothelial senescence was assessed by p16 (CDKN2A), p21 (CDKN1A), and LMNB1 immunostaining and senescence-associated β-galactosidase (SA-β-gal), and the SASP signature (IL6, MMP3, CXCL10) was quantified spatially and by enzyme-linked immunosorbent assay (Fig. S14). Cell-free mitochondrial DNA was measured by droplet-digital PCR in plasma and in laser-microdissected peritubular vascular bundles, with cGAS, STING (TMEM173), and TLR9 transcripts quantified in rarefied-territory spots. PIEZO1-mediated mechano-priming was characterized in rarefied-territory endothelium (Figs. S13 and S15).

### In vitro endothelial senescence and hypoxia/reoxygenation model

Primary HRGECs (ScienCell #4000) were used. Replicative senescence was modeled by serial passaging: Young (P3 to P5) and senescent (P15+, SA-β-gal > 70%) cells were subjected to hypoxia (1% O₂, 24 h) and reoxygenation (21% O₂, 6 h). Mitochondrial function was assessed by MitoSOX Red, JC-1, and cytosolic-mtDNA quantification; pyroptosis was assessed by Western blotting (NLRP3, cleaved caspase-1, GSDMD-N), GSDMD-N immunofluorescence, and LDH release. Pharmacological rescue used MitoQ (500 nM) or MCC950 (10 μM).

To weigh the relative contributions of inflammaging and hypoxia, experiments were organized as a 2 × 2 factorial design (young passage 4 versus replicatively aged passage 15 × normoxia 21% O₂ versus hypoxia 1% O₂, 48 h), analyzed by 2-way ANOVA with explicit testing of the aging × hypoxia interaction; the proportion of variance attributable to each factor and their interaction was quantified by variance partitioning (variancePartition), with ASC-speck formation as the primary readout (Fig. S16). PIEZO1-mediated mechano-priming was tested with the PIEZO1 agonist Yoda1 (0 to 10 μM), measuring ASC-speck formation and the extracellular-ATP threshold for NLRP3 oligomerization.

### Statistical analysis

The primary endpoint was MAREs: a composite of ESRD, sustained doubling of serum creatinine, or death from renal causes. Event-free survival was estimated by Kaplan–Meier curves and compared by the log-rank test. Given the substantial competing risk of cardiovascular death, the Fine–Gray subdistribution hazard model was used to estimate the cumulative incidence of renal events [37], and the cluster × treatment interaction was tested in Cox models. Discrimination was assessed by the time-dependent AUC, and the prognostic value of the AI phenotype over the standard-of-care model by the net reclassification improvement and integrated discrimination improvement [38].

Among stented patients, 24-month response was classified a priori as strong responder (no MARE and eGFR change ≥ −5% from baseline), modest responder (no MARE and eGFR decline 5% to 20%), nonresponder (no MARE and eGFR decline > 20%), or progressor (MARE within 24 months or eGFR decline > 50%); favorable response combined strong and modest responders. Distributions across phenotypes were compared by Pearson χ^2^ with Wilson CIs.

Absolute event rates were expressed as the 3-year cumulative incidence of MARE estimated by the Aalen–Johansen method with cardiovascular death as a competing risk (cmprsk, v2.2-12); the number needed to treat and its Newcombe–Wilson CI were derived from the between-arm difference in cumulative incidence, and the crude proportions were reconciled with the competing-risk estimates (Table 3E). Because stenting rates differed across phenotypes, an additional within-phenotype 1:1 nearest-neighbor propensity score-matched sensitivity analysis was performed (caliper 0.2 SD of the logit propensity score; MatchIt, v4.5) on 9 covariates, with balance assessed by standardized mean differences (<0.10 considered balanced; 354 matched pairs; Table S12).

The relationship between angiographic stenosis severity and AI perfusion metrics was quantified by Spearman rank correlation (metrics non-normal by Shapiro–Wilk), with 10,000-replicate bootstrap CIs and partial correlations adjusted for age and eGFR (ppcor, v1.1); differences in correlation strength across phenotypes were tested by the Fisher *r*-to-*z* transformation (Table S14). Predictors of 3-year event-free survival were identified by multivariable logistic regression in all 395 stented patients (candidate clinical, laboratory, imaging, and AI variables), with discrimination internally validated by 1,000-iteration bootstrap optimism correction, calibration assessed by the Hosmer–Lemeshow test and calibration slope, and the Brier score reported (rms, v6.7); the full models for all stented patients and for the Delayed phenotype are reported in Table S11A and B, respectively. Within the Delayed phenotype, favorable and unfavorable responders were compared by Student’s t test, Mann–Whitney *U* test, or χ^2^ with standardized mean differences.

For animal experiments, 2-group comparisons used the unpaired Student’s *t* test or Mann–Whitney *U* test and multiple-group comparisons used one-way ANOVA with Tukey post hoc; the age × surgery interaction was tested by 2-way ANOVA. In the External Validation Cohort, AI risk score distributions across ultrasound vendors were compared by the Kruskal–Wallis test. Multiple-comparison correction used the Benjamini–Hochberg false-discovery-rate procedure for the spatial-transcriptomic differential-expression analyses and the Bonferroni procedure for the prespecified murine baseline comparisons. All tests were 2-sided with *P* < 0.05 considered significant. Analyses used R (v4.3.0) and Python (v3.9), with the XGBoost (v1.7), SHAP (v0.43), MatchIt (v4.5), cmprsk (v2.2-12), ppcor (v1.1), rms (v6.7), car, boot, and variancePartition packages.

## Ethical Approval

This multi-center study was conducted in accordance with the principles of the Declaration of Helsinki. The clinical protocol was approved by the Institutional Review Board (Ethics Committee) of Beijing Hospital (approval no. 2025BJYYEC-KY371-02) and by the institutional review boards of all participating satellite centers. For the prospective Multimodal Validation Cohort, written informed consent—including consent for 10x Visium spatial-transcriptomic sequencing of biopsy tissue cores—was obtained from every participant prior to enrollment. For the retrospective Discovery Cohort and the External Validation Cohort, the institutional review boards approved a waiver of individual informed consent on the basis that the analysis used preexisting, deidentified CEUS and clinical data and posed no additional risk to patients.

All animal experiments were approved by the IACUC of the Second Affiliated Hospital of Zhejiang University School of Medicine (approval no. ZJCLA-IACUC-20011469) and were performed in accordance with the NIH Guide for the Care and Use of Laboratory Animals and reported in compliance with the ARRIVE 2.0 guidelines.

## Data Availability

All raw data underlying the findings of this study are available from the corresponding author, J.R. (rjh13910813603@163.com), upon reasonable request and subject to appropriate justification. Because the deidentified clinical data, the raw contrast-enhanced ultrasound (CEUS) DICOM video loops (which contain protected health information), the trained Renal-Video-AI model weights, and the spatial-transcriptomic data (10x Visium) are governed by patient-privacy obligations, institutional data-sharing agreements, the approvals of the participating centers’ institutional review boards, and intellectual-property review, access requires a formal data-use agreement and a description of the intended use that protects patient confidentiality. Requests will be evaluated by the corresponding author and the relevant institutions, with an expected response time of 30 business days. Representative deidentified CEUS video clips for the cases shown in the figures are provided in the Supplementary Materials. All other data needed to evaluate the conclusions of the paper are present in the paper and/or the Supplementary Materials.
