# Supplementary material for: Spatiotemporal Deep Video-Phenomapping Decodes Microvascular Rarefaction in Middle-Aged and Elder Renovascular Hypertension: A Multi-Modal Study Integrating Spatial Transcriptomics and Mitochondrial Pyroptosis
Source: Research (Wash D C). 2026 Jul 3;9:1339. doi: 10.34133/research.1339 (PMC13329644; doi:10.34133/research.1339)
Supplement: Supplementary 1 — Tables S1 to S16 Figs. S1 to S16 [file research.1339.f1.zip › Supplementary Figure S1-16.docx]

## Supplementary Materials

**Supplementary Figures**

Fig. S1. Determination and stability of the optimal number of phenotypes.

Fig. S2. Quality control of CEUS video motion registration.

Fig. S3. External validation and scanner independence.

Fig. S4. In vivo rescue by MitoTEMPO and MCC950.

Fig. S5. Uncropped full-length Western blots (source data).

Fig. S6. Participant-flow diagrams for the three cohorts.

Fig. S7. Extended spatial-transcriptomic deconvolution and network analysis.

Fig. S8. Extended renal-injury characterization of the 2K1C model.

Fig. S9. Interpretable AI feature importance and per-feature distributions.

Fig. S10. Microvascular density across the three phenotypes.

Fig. S11. Propensity-score-matched treatment-effect analysis.

Fig. S12. Multi-organ safety of MCC950.

Fig. S13. PIEZO1-mediated mechano-priming of the inflammasome.

Fig. S14. Endothelial senescence and the senescence-associated secretory phenotype.

Fig. S15. HRGEC validation of PIEZO1 mechano-priming.

Fig. S16. Relative contributions of inflammaging and hypoxia.
