# Supplementary material for: Spatiotemporal Deep Video-Phenomapping Decodes Microvascular Rarefaction in Middle-Aged and Elder Renovascular Hypertension: A Multi-Modal Study Integrating Spatial Transcriptomics and Mitochondrial Pyroptosis
Source: Research (Wash D C). 2026 Jul 3;9:1339. doi: 10.34133/research.1339 (PMC13329644; doi:10.34133/research.1339)
Supplement: Supplementary 1 — Tables S1 to S16 Figs. S1 to S16 [file research.1339.f1.zip › Supplementary Table S1-16-5.25.pdf]

Supplementary Table S1

Baseline Characteristics of the External Validation Cohort (N = 122) and the Multimodal Validation Cohort (N = 57), Stratified by AI-Identified Hemodynamic Phenotypes

Panel A: External Validation Cohort (N = 122)

Patients recruited from 6 geographically distinct satellite centers using 4 different ultrasound systems (GE, Canon/Toshiba, Siemens, Philips). Inclusion and exclusion criteria identical to the Discovery Cohort.

Variable	Overall (N = 122)	Cluster 1: Preserved (n = 40)	Cluster 2: Delayed (n = 52)	Cluster 3: Rarefied (n = 30)	P value
Demographics					
Age, years	67.4 ± 9.0	63.8 ± 8.2	67.2 ± 8.6	73.2 ± 8.0	<0.001
Male sex, n (%)	74 (60.7)	24 (60.0)	32 (61.5)	18 (60.0)	0.99
BMI, kg/m²	25.4 ± 3.6	25.6 ± 3.2	25.8 ± 3.8	24.2 ± 4.0	0.28
Current smoker, n (%)	38 (31.1)	10 (25.0)	16 (30.8)	12 (40.0)	0.38
Comorbidities					
Hypertension duration, years	14.2 ± 7.8	11.8 ± 6.2	14.4 ± 7.4	18.2 ± 9.0	0.004
Diabetes mellitus, n (%)	42 (34.4)	9 (22.5)	18 (34.6)	15 (50.0)	0.046
Coronary artery disease, n (%)	32 (26.2)	8 (20.0)	14 (26.9)	10 (33.3)	0.44
Prior stroke/TIA, n (%)	15 (12.3)	3 (7.5)	6 (11.5)	6 (20.0)	0.26
Peripheral artery disease, n (%)	20 (16.4)	4 (10.0)	8 (15.4)	8 (26.7)	0.16
Atrial fibrillation, n (%)	12 (9.8)	3 (7.5)	5 (9.6)	4 (13.3)	0.72
Renal Function					
eGFR, mL/min/1.73m²	54.8 ± 22.4	70.2 ± 17.8	54.6 ± 18.6	33.4 ± 14.8	<0.001
Serum creatinine, μmol/L	132.4 ± 48.6	100.2 ± 24.8	130.8 ± 38.4	184.2 ± 52.6	<0.001
Proteinuria ≥ 1 g/day, n (%)	28 (23.0)	4 (10.0)	12 (23.1)	12 (40.0)	0.01
CKD Stage 1–2, n (%)	42 (34.4)	24 (60.0)	14 (26.9)	4 (13.3)	<0.001
CKD Stage 3a–3b, n (%)	50 (41.0)	14 (35.0)	26 (50.0)	10 (33.3)	
CKD Stage 4–5, n (%)	30 (24.6)	2 (5.0)	12 (23.1)	16 (53.3)	
Anatomical Stenosis (CTA/DSA)					
Stenosis degree, %, mean ± SD	66.8 ± 13.2	62.4 ± 11.6	66.8 ± 12.8	74.2 ± 14.0	<0.001
Mild stenosis (50–60%), n (%)	36 (29.5)	17 (42.5)	14 (26.9)	5 (16.7)	0.04
Moderate stenosis (60–70%), n (%)	48 (39.3)	15 (37.5)	22 (42.3)	11 (36.7)	
Severe stenosis (>70%), n (%)	38 (31.1)	8 (20.0)	16 (30.8)	14 (46.7)	

Bilateral RAS, n (%)	18 (14.8)	3 (7.5)	8 (15.4)	7 (23.3)	0.15
Doppler Hemodynamics					
Resistive Index (RI)	0.72 ± 0.10	0.62 ± 0.06	0.72 ± 0.07	0.83 ± 0.06	<0.001
Peak systolic velocity, cm/s	258 ± 90	238 ± 78	260 ± 86	286 ± 104	0.08
Baseline Medications					
ACEi/ARB, n (%)	92 (75.4)	30 (75.0)	40 (76.9)	22 (73.3)	0.93
Statin, n (%)	86 (70.5)	28 (70.0)	38 (73.1)	20 (66.7)	0.81
Antiplatelet, n (%)	78 (63.9)	24 (60.0)	34 (65.4)	20 (66.7)	0.80
Calcium channel blocker, n (%)	56 (45.9)	16 (40.0)	24 (46.2)	16 (53.3)	0.53
Treatment During Follow-up					
Renal artery stenting, n (%)	40 (32.8)	10 (25.0)	20 (38.5)	10 (33.3)	0.39
Medical therapy alone, n (%)	82 (67.2)	30 (75.0)	32 (61.5)	20 (66.7)	
Ultrasound System Distribution					
GE Logiq E9, n (%)	34 (27.9)	12 (30.0)	14 (26.9)	8 (26.7)	0.88
Canon/Toshiba Aplio i800, n (%)	28 (23.0)	8 (20.0)	12 (23.1)	8 (26.7)	
Siemens Acuson Sequoia, n (%)	32 (26.2)	10 (25.0)	14 (26.9)	8 (26.7)	
Philips EPIQ 7, n (%)	28 (23.0)	10 (25.0)	12 (23.1)	6 (20.0)	
Clinical Outcomes (Median Follow-up: 3.2 Years, IQR 1.8–4.5)					
MARE composite, n (%)	32 (26.2)	4 (10.0)	12 (23.1)	16 (53.3)	<0.001
ESRD, n (%)	12 (9.8)	1 (2.5)	4 (7.7)	7 (23.3)	0.006
Doubling of creatinine, n (%)	22 (18.0)	2 (5.0)	8 (15.4)	12 (40.0)	<0.001
Death from renal causes, n (%)	6 (4.9)	1 (2.5)	2 (3.8)	3 (10.0)	0.26
Cardiovascular death, n (%)	10 (8.2)	2 (5.0)	4 (7.7)	4 (13.3)	0.43
All-cause mortality, n (%)	16 (13.1)	3 (7.5)	6 (11.5)	7 (23.3)	0.12
Lost to follow-up, n (%)	5 (4.1)	2 (5.0)	2 (3.8)	1 (3.3)	0.93

Data are mean ± SD or n (%). P values from one-way ANOVA (continuous) or Fisher's exact test (categorical, preferred over chi-square due to smaller cell sizes). Ultrasound system distribution is balanced across phenotypes (P = 0.88), confirming no vendor-phenotype confounding. MARE = Major Adverse Renal Events. IQR = interquartile range. The External Validation Cohort phenotype proportions (32.8% / 42.6% / 24.6%) are comparable to the Discovery Cohort (41.6% / 35.5% / 22.9%; chi-square P = 0.12 for distribution homogeneity).

Panel B: Multimodal Validation Cohort (N = 57)

Prospectively enrolled patients with confirmed ARAS (≥50%) who underwent paired CEUS and ultrasound-guided renal biopsy within <48 hours at the lead center (Beijing Hospital). All 57 patients completed 10x Visium spatial transcriptomics.

Variable	Overall (N = 57)	Cluster 1: Preserved (n = 20)	Cluster 2: Delayed (n = 22)	Cluster 3: Rarefied (n = 15)	P value
Demographics					
Age, years	68.2 ± 8.4	64.2 ± 7.8	68.4 ± 8.2	74.6 ± 6.8	0.001
Male sex, n (%)	34 (59.6)	12 (60.0)	14 (63.6)	8 (53.3)	0.79
BMI, kg/m²	25.0 ± 3.4	25.4 ± 3.0	25.2 ± 3.6	24.0 ± 3.8	0.44
Comorbidities					
Hypertension duration, years	14.8 ± 7.6	11.6 ± 5.8	15.2 ± 7.2	19.4 ± 8.4	0.006
Diabetes mellitus, n (%)	20 (35.1)	5 (25.0)	8 (36.4)	7 (46.7)	0.37
Coronary artery disease, n (%)	16 (28.1)	4 (20.0)	6 (27.3)	6 (40.0)	0.40
Prior stroke/TIA, n (%)	7 (12.3)	1 (5.0)	3 (13.6)	3 (20.0)	0.37
Peripheral artery disease, n (%)	10 (17.5)	2 (10.0)	4 (18.2)	4 (26.7)	0.41
Renal Function					
eGFR, mL/min/1.73m²	52.6 ± 21.8	68.4 ± 16.2	52.8 ± 17.4	30.6 ± 13.2	<0.001
Serum creatinine, μmol/L	136.8 ± 50.2	102.4 ± 22.6	134.2 ± 36.8	192.8 ± 54.4	<0.001
Proteinuria ≥ 1 g/day, n (%)	14 (24.6)	2 (10.0)	5 (22.7)	7 (46.7)	0.03
CKD Stage 1–2, n (%)	18 (31.6)	12 (60.0)	5 (22.7)	1 (6.7)	<0.001
CKD Stage 3a–3b, n (%)	24 (42.1)	7 (35.0)	12 (54.5)	5 (33.3)	
CKD Stage 4–5, n (%)	15 (26.3)	1 (5.0)	5 (22.7)	9 (60.0)	
Anatomical Stenosis					
Stenosis degree, %, mean ± SD	67.2 ± 12.8	62.8 ± 11.0	67.4 ± 12.2	73.8 ± 13.6	0.02
Mild stenosis (50–60%), n (%)	17 (29.8)	9 (45.0)	6 (27.3)	2 (13.3)	0.06
Moderate stenosis (60–70%), n (%)	22 (38.6)	7 (35.0)	10 (45.5)	5 (33.3)	
Severe stenosis (>70%), n (%)	18 (31.6)	4 (20.0)	6 (27.3)	8 (53.3)	
Doppler Hemodynamics					
Resistive Index (RI)	0.72 ± 0.10	0.64 ± 0.06	0.72 ± 0.07	0.84 ± 0.05	<0.001
Baseline Medications					
ACEi/ARB, n (%)	44 (77.2)	16 (80.0)	17 (77.3)	11 (73.3)	0.88
Statin, n (%)	40 (70.2)	14 (70.0)	16 (72.7)	10 (66.7)	0.92
Antiplatelet, n (%)	36 (63.2)	12 (60.0)	14 (63.6)	10 (66.7)	0.91
Biopsy and Spatial Transcriptomics Data					

Time from CEUS to biopsy, hours	18.4 ± 10.2	17.8 ± 9.6	18.2 ± 10.8	19.6 ± 10.4	0.86
Biopsy core length, mm	14.2 ± 3.8	14.8 ± 3.4	14.4 ± 4.0	13.0 ± 4.2	0.36
Glomeruli per biopsy, n	12.4 ± 4.6	13.2 ± 4.2	12.8 ± 4.8	10.6 ± 4.6	0.19
Globally sclerosed glomeruli, %	22.4 ± 18.6	10.2 ± 8.4	20.8 ± 14.2	42.8 ± 18.6	<0.001
Interstitial fibrosis score (0–3)	1.2 ± 0.8	0.6 ± 0.4	1.2 ± 0.6	2.1 ± 0.7	<0.001
Arteriolar hyalinosis score (0–3)	1.4 ± 0.8	0.8 ± 0.5	1.4 ± 0.7	2.2 ± 0.6	<0.001
Tissue Quality for Spatial Transcriptomics					
RNA Integrity Number (RIN)	7.8 ± 0.6	7.9 ± 0.5	7.8 ± 0.6	7.6 ± 0.7	0.28
Visium spots on tissue, n	3,180 ± 620	3,340 ± 580	3,220 ± 640	2,880 ± 580	0.06
Spots passing QC, n (%)	2,960 ± 580 (93.1%)	3,120 ± 540 (93.4%)	3,000 ± 600 (93.2%)	2,680 ± 520 (93.1%)	0.07
Median genes per spot	3,420 ± 680	3,580 ± 620	3,440 ± 700	3,160 ± 640	0.12
Median UMIs per spot	8,240 ± 2,180	8,620 ± 2,040	8,340 ± 2,240	7,480 ± 2,120	0.18
Sequencing depth, reads/spot	64,800 ± 8,400	66,200 ± 7,800	65,000 ± 8,600	62,400 ± 8,800	0.32
Mitochondrial gene fraction, %	8.4 ± 3.2	7.2 ± 2.4	8.2 ± 3.0	10.4 ± 3.8	0.01
Spatial Gene Signature Scores (All 57 Samples, Spot-Level Aggregated per Patient)					
Endothelial Integrity Score	0.42 ± 0.28	0.68 ± 0.18	0.42 ± 0.16	0.10 ± 0.08	<0.001
Hypoxia Score	0.38 ± 0.24	0.14 ± 0.08	0.36 ± 0.14	0.72 ± 0.16	<0.001
Fibrosis Score	0.36 ± 0.26	0.12 ± 0.06	0.34 ± 0.14	0.70 ± 0.18	<0.001
Pyroptosis Score	0.32 ± 0.24	0.10 ± 0.06	0.30 ± 0.12	0.66 ± 0.16	<0.001
Mitochondrial Dysfunction Score	0.34 ± 0.22	0.12 ± 0.08	0.32 ± 0.14	0.68 ± 0.14	<0.001
Spatial Registration Quality					
AI-Endothelial Score correlation (r)	−0.75 ± 0.08	−0.72 ± 0.10	−0.74 ± 0.08	−0.78 ± 0.06	0.12
AI-Pyroptosis Score correlation (r)	0.78 ± 0.06	0.74 ± 0.08	0.78 ± 0.06	0.82 ± 0.04	0.008
Registration landmarks identified, n	4.2 ± 1.0	4.4 ± 0.8	4.2 ± 1.0	4.0 ± 1.2	0.52
Affine registration error, μm	82 ± 28	78 ± 24	80 ± 26	90 ± 34	0.38
Clinical Outcomes (Median Follow-up: 2.8 Years, IQR 1.6–3.8)					
MARE composite, n (%)	16 (28.1)	2 (10.0)	5 (22.7)	9 (60.0)	0.002
ESRD, n (%)	5 (8.8)	0 (0)	1 (4.5)	4 (26.7)	0.008
Doubling of creatinine, n (%)	12 (21.1)	1 (5.0)	4 (18.2)	7 (46.7)	0.008
All-cause mortality, n (%)	6 (10.5)	1 (5.0)	2 (9.1)	3 (20.0)	0.30

Data are mean ± SD or n (%). P values from one-way ANOVA (continuous) or Fisher's exact test (categorical). Time from CEUS to biopsy confirms temporal alignment (<48 hours). RIN ≥ 7.0 was the quality threshold for Visium; all 57 samples met this criterion. Spots passing QC: spots with ≥500 genes, ≥1,000 UMIs, and mitochondrial fraction <20%. Gene signature scores were computed using Seurat AddModuleScore() and averaged across all spots per patient (see Supplementary Methods S3 for gene lists). Spatial registration quality:

AI-Endothelial Score correlation represents the Pearson r between AI Perfusion Risk Score and Endothelial Integrity Score across spatially matched spots per patient (main text reports overall $r = -0.75$). The negative correlation confirms that AI-detected "cold spots" correspond to endothelial loss. Interstitial fibrosis and arteriolar hyalinosis graded by a renal pathologist blinded to AI phenotype using the Banff classification (0 = none, 1 = mild, 2 = moderate, 3 = severe).

Panel C: Cohort Comparability Summary

Cross-cohort comparison to assess generalizability. P values test for differences among the three cohorts (overall, not stratified by phenotype).

Variable	Discovery (N = 1,226)	External Validation (N = 122)	Multimodal Validation (N = 57)	P value
Demographics				
Age, years	68.0 ± 9.0	67.4 ± 9.0	68.2 ± 8.4	0.82
Male sex, %	60.6	60.7	59.6	0.98
BMI, kg/m²	25.2 ± 3.5	25.4 ± 3.6	25.0 ± 3.4	0.84
Key Comorbidities				
Diabetes mellitus, %	32.8	34.4	35.1	0.80
Coronary artery disease, %	26.6	26.2	28.1	0.94
Renal Function				
eGFR, mL/min/1.73m²	56.8 ± 22.4	54.8 ± 22.4	52.6 ± 21.8	0.34
Proteinuria ≥ 1 g/day, %	21.4	23.0	24.6	0.68
Stenosis				
Stenosis degree, %	66.2 ± 13.6	66.8 ± 13.2	67.2 ± 12.8	0.86
Severe stenosis (>70%), %	28.1	31.1	31.6	0.52
Hemodynamics				
Resistive Index (RI)	0.71 ± 0.10	0.72 ± 0.10	0.72 ± 0.10	0.72
AI Phenotype Distribution				
Cluster 1: Preserved, %	41.6	32.8	35.1	0.12
Cluster 2: Delayed, %	35.5	42.6	38.6	
Cluster 3: Rarefied, %	22.9	24.6	26.3	
Outcomes				
MARE rate, %	21.9	26.2	28.1	0.18
Median follow-up, years	3.5	3.2	2.8	0.04

P values from one-way ANOVA (continuous) or chi-square test (categorical) comparing three cohorts. The Discovery Cohort has a slightly higher proportion of Cluster 1 (Preserved) patients and longer follow-up. All other key variables are well-matched across cohorts, supporting generalizability. The shorter follow-up in the Multimodal cohort reflects its later enrollment period (June 2023–January 2025). Overall mean age across all three cohorts: 68.0 years (pooled weighted mean), consistent with the target population of aging ARAS (≥45 years).

Supplementary Table S2

Propensity Score–Matched Analysis of Renal Artery Stenting vs. Medical Therapy Stratified by AI-Identified Hemodynamic Phenotype

Panel A: Propensity Score Model Specification

A multivariable logistic regression model was constructed to estimate the propensity score (PS) — the predicted probability of receiving renal artery stenting (PTRAS) vs. medical therapy alone — for each patient within each AI phenotype cluster separately. Separate PS models were fit per cluster to account for potential heterogeneity in treatment allocation patterns across phenotypes.

Covariate in PS Model	Cluster 1 OR (95% CI)	Cluster 2 OR (95% CI)	Cluster 3 OR (95% CI)
Patient Factors			
Age, per 10-year increase	1.08 (0.82–1.42)	1.12 (0.84–1.50)	0.92 (0.62–1.36)
Male sex	1.22 (0.82–1.82)	1.18 (0.80–1.74)	1.28 (0.72–2.28)
Diabetes mellitus	0.88 (0.58–1.34)	1.08 (0.72–1.62)	0.94 (0.50–1.78)
Coronary artery disease	1.15 (0.72–1.84)	1.22 (0.82–1.82)	1.08 (0.56–2.08)
Renal Factors			
eGFR, per 10 mL/min decrease	1.18 (1.02–1.36)	1.24 (1.08–1.42)	1.12 (0.92–1.36)
Proteinuria ≥ 1 g/day	1.42 (0.82–2.46)	1.56 (1.02–2.38)	1.28 (0.68–2.42)
CKD Stage (≥3b vs. <3b)	1.35 (0.86–2.12)	1.28 (0.86–1.90)	1.18 (0.62–2.26)
Anatomical and Hemodynamic Factors			
Stenosis degree, per 10% increase	1.85 (1.42–2.42)	1.72 (1.36–2.18)	1.68 (1.18–2.38)
Bilateral RAS	1.48 (0.78–2.82)	1.62 (0.96–2.74)	1.38 (0.68–2.82)
Resistive Index ≥ 0.80	0.72 (0.42–1.24)	0.68 (0.42–1.10)	0.82 (0.42–1.60)
Model Performance			
C-statistic of PS model	0.74 (0.68–0.80)	0.72 (0.66–0.78)	0.71 (0.62–0.80)
Hosmer-Lemeshow P	0.62	0.54	0.48

OR = odds ratio for receiving stenting. PS model C-statistics of 0.71–0.74 indicate adequate discrimination without overfitting (C > 0.80 would suggest overfitting or near-deterministic treatment assignment). Stenosis degree was the strongest predictor of stenting across all clusters, reflecting clinical practice where more severe stenosis drives intervention decisions. Hosmer-Lemeshow P > 0.05 confirms adequate PS model calibration.

Panel B: Matching Procedure and Cohort Sizes

Matching method: Nearest-neighbor 1:1 matching without replacement on the logit of the propensity score, with a caliper width of 0.20 standard deviations. Matching was performed within each AI phenotype cluster separately using the MatchIt package (v4.5.5) in R.

	Cluster 1: Preserved	Cluster 2: Delayed	Cluster 3: Rarefied
Pre-Matching Sample			
Total patients	510	435	281
Stented, n (%)	142 (27.8)	168 (38.6)	85 (30.2)
Medical therapy, n (%)	368 (72.2)	267 (61.4)	196 (69.8)
Post-Matching Sample			
Matched pairs, n	132	158	78
Stented (matched), n	132	158	78
Medical (matched), n	132	158	78
Unmatched stented (excluded), n	10	10	7
Matching rate, %	93.0	94.0	91.8
Propensity Score Distribution			
PS median (IQR), stented	0.32 (0.22–0.44)	0.42 (0.30–0.54)	0.34 (0.24–0.48)
PS median (IQR), medical	0.24 (0.16–0.36)	0.34 (0.22–0.48)	0.28 (0.18–0.42)
PS overlap (common support), %	92.4	94.8	90.6
Post-match PS difference, median	0.008	0.006	0.012

Matching rate = matched stented / total stented × 100%. Unmatched patients were excluded from the PSM analysis (10, 10, and 7 patients from Clusters 1–3 respectively could not find a match within the caliper). PS overlap indicates the proportion of the PS distribution that was common between stented and medical groups. Post-match PS difference confirms excellent matching precision (all median differences < 0.02). IQR = interquartile range.

Panel C: Covariate Balance Before and After Propensity Score Matching

Standardized Mean Differences (SMD) between stented and medical therapy groups before and after matching, shown for each AI phenotype cluster. An SMD < 0.10 (green) indicates adequate balance; SMD > 0.10 (red) indicates imbalance.

Covariate	C1 Pre-match	C1 Post-match	C2 Pre-match	C2 Post-match	C3 Pre-match	C3 Post-match
Demographics						
Age	0.18	0.04	0.22	0.05	0.16	0.06
Male sex	0.08	0.02	0.12	0.03	0.10	0.04
BMI	0.06	0.02	0.08	0.03	0.04	0.02
Current smoker	0.04	0.01	0.06	0.02	0.08	0.03
Comorbidities						
Hypertension duration	0.14	0.04	0.16	0.05	0.12	0.04
Diabetes mellitus	0.08	0.03	0.12	0.04	0.10	0.05
Coronary artery disease	0.12	0.04	0.14	0.05	0.08	0.03
Prior stroke/TIA	0.06	0.02	0.04	0.02	0.08	0.04
Peripheral artery disease	0.10	0.03	0.08	0.03	0.06	0.02
Atrial fibrillation	0.04	0.01	0.06	0.02	0.04	0.02
Renal Function						
eGFR	0.24	0.06	0.28	0.07	0.20	0.08
Serum creatinine	0.22	0.05	0.26	0.06	0.18	0.07
Proteinuria ≥ 1 g/day	0.16	0.04	0.18	0.05	0.14	0.06
CKD Stage	0.20	0.06	0.22	0.06	0.16	0.07
Anatomical Stenosis						
Stenosis degree	0.42	0.08	0.38	0.07	0.34	0.09
Bilateral RAS	0.14	0.04	0.16	0.05	0.12	0.05
Hemodynamics and Medications						
Resistive Index	0.16	0.05	0.18	0.06	0.12	0.05
ACEi/ARB use	0.08	0.02	0.10	0.03	0.06	0.02
Statin use	0.06	0.02	0.08	0.03	0.04	0.02
Antiplatelet use	0.10	0.03	0.12	0.04	0.08	0.03
Summary						
Mean absolute SMD	0.134	0.036	0.153	0.042	0.114	0.042
Max SMD	0.42	0.08	0.38	0.07	0.34	0.09
Covariates with SMD > 0.10	8 / 16	0 / 16	10 / 16	0 / 16	6 / 16	0 / 16

SMD = Standardized Mean Difference (absolute value) between stented and medical therapy groups. Green

shading: SMD ≤ 0.10 (adequate balance). Red shading: SMD > 0.10 (imbalance). Before matching, stenosis degree was the most imbalanced covariate across all clusters (SMD 0.34–0.42), reflecting the clinical reality that more severely stenosed patients were preferentially referred for stenting. After matching, all covariates achieved SMD < 0.10 in all three clusters, confirming successful elimination of measured confounding. The mean absolute SMD decreased from 0.114–0.153 (pre-match) to 0.036–0.042 (post-match).

Panel D: Treatment Effects Within Propensity Score–Matched Strata

Hazard ratios for stenting vs. medical therapy within each AI phenotype cluster, estimated from Cox proportional hazards models in the matched cohorts.

Outcome	Cluster 1: Preserved (132 pairs)	Cluster 2: Delayed (158 pairs)	Cluster 3: Rarefied (78 pairs)
Primary Endpoint: MARE (Stenting vs. Medical Therapy)			
Events — Stented, n (%)	14 (10.6)	22 (13.9)	36 (46.2)
Events — Medical, n (%)	13 (9.8)	38 (24.1)	34 (43.6)
HR (95% CI)	0.96 (0.65–1.42)	0.55 (0.36–0.84)	1.08 (0.68–1.72)
P value	0.84	0.006	0.74
Interpretation	No benefit	Significant benefit	Futile
Secondary Endpoints (Stenting vs. Medical)			
ESRD, HR (95% CI)	0.92 (0.42–2.02)	0.48 (0.24–0.96)	1.12 (0.58–2.16)
ESRD, P value	0.84	0.04	0.72
Doubling of creatinine, HR	0.98 (0.60–1.60)	0.52 (0.32–0.86)	1.04 (0.62–1.76)
Doubling of creatinine, P	0.94	0.01	0.88
All-cause mortality, HR	1.02 (0.58–1.80)	0.72 (0.42–1.22)	0.88 (0.50–1.56)
All-cause mortality, P	0.94	0.22	0.66
eGFR change at 1 year, mL/min	−1.4 vs. −1.0 P = 0.68	−2.8 vs. −6.2 P = 0.004	−11.8 vs. −12.2 P = 0.82
eGFR change at 3 years, mL/min	−3.0 vs. −2.4 P = 0.56	−6.4 vs. −14.2 P < 0.001	−23.4 vs. −24.0 P = 0.78
Renal Function Trajectory: Annual eGFR Slope (mL/min/year)			
Stented group	−1.0 ± 2.2	−2.2 ± 2.4	−8.0 ± 3.4
Medical group	−0.8 ± 1.8	−4.8 ± 2.8	−8.4 ± 3.6
Difference (stented − medical)	−0.2 (NS)	+2.6 (P = 0.002)	+0.4 (NS)

HR = Hazard Ratio (stenting vs. medical therapy). HR < 1.0 favors stenting. P values from stratified Cox models conditioning on matched pairs. MARE = composite of ESRD, sustained doubling of creatinine, or death from renal causes. eGFR changes shown as mean in stented vs. medical (within-pair comparison by paired t-test). Annual eGFR slope estimated by linear mixed-effects model. In Cluster 2 (Delayed), stenting preserved an additional 2.6 mL/min/year of eGFR compared to medical therapy, corresponding to approximately 7.8 mL/min over 3 years — a clinically meaningful difference. Note: All-cause mortality showed no significant benefit from stenting in any cluster, consistent with the CORAL trial result that stenting does not reduce overall mortality.

Panel E: Sensitivity Analyses and Robustness Checks

E1. Comparison of Unmatched vs. PSM Treatment Effect Estimates

Cluster	Unmatched HR (95% CI)	Unmatched P	PSM HR (95% CI)	PSM P	Consistent?
C1: Preserved	0.98 (0.75–1.25)	0.85	0.96 (0.65–1.42)	0.84	Yes
C2: Delayed	0.52 (0.35–0.78)	0.002	0.55 (0.36–0.84)	0.006	Yes
C3: Rarefied	1.05 (0.70–1.50)	0.72	1.08 (0.68–1.72)	0.74	Yes
Interaction P	<0.01		0.008		Yes

The PSM estimates are highly consistent with the unmatched (Table 3) results. Point estimates shift modestly after matching (expected as confounding by stenosis severity is removed), but the qualitative conclusions are identical: stenting benefits only Cluster 2. The interaction P remains significant after matching (P = 0.008 vs. P < 0.01 unmatched).

E2. Inverse Probability of Treatment Weighting (IPTW) Analysis

As an alternative to PSM, we performed IPTW analysis using stabilized weights derived from the same propensity score model. IPTW has the advantage of retaining the full sample size and avoiding exclusion of unmatched patients.

Cluster	IPTW HR (95% CI)	P value	Interpretation
C1: Preserved (n = 510)	0.94 (0.72–1.22)	0.64	No benefit
C2: Delayed (n = 435)	0.54 (0.38–0.78)	0.001	Significant benefit
C3: Rarefied (n = 281)	1.02 (0.72–1.46)	0.90	Futile
Interaction P (IPTW)	0.004		Significant

Stabilized IPTW weights were trimmed at the 1st and 99th percentiles to mitigate extreme weights. Effective sample sizes after weighting: Cluster 1 = 482, Cluster 2 = 412, Cluster 3 = 268. The IPTW results closely replicate both the PSM and unmatched analyses, providing triple convergence across three causal inference approaches.

E3. E-Value Sensitivity Analysis for Unmeasured Confounding

The E-value quantifies the minimum strength of association (on the risk ratio scale) that an unmeasured confounder would need to have with both treatment assignment and the outcome to fully explain away the observed treatment effect. A high E-value indicates robustness to unmeasured confounding.

Parameter	Cluster 1	Cluster 2	Cluster 3
E-Value for MARE Treatment Effect			

Point estimate (PSM HR)	0.96	0.55	1.08
E-value for point estimate	1.22	2.98	1.35
E-value for CI limit closest to 1.0	1.0 (crosses null)	1.78	1.0 (crosses null)
Interpretation			
Unmeasured confounder needed?	Not applicable (HR already null)	An unmeasured confounder would need RR ≥ 2.98 with both stenting and MARE to explain away the benefit	Not applicable (HR already null)

E-values calculated using the formula: E-value = HR + sqrt(HR × (HR − 1)) for HR < 1 (converted to protective scale). For Cluster 2, the E-value of 2.98 means an unmeasured confounder would need to be associated with nearly 3-fold increased likelihood of both receiving stenting AND experiencing MARE to fully explain the observed benefit. Given that all major known confounders (eGFR, stenosis, comorbidities, medications) are accounted for in the PS model, this threshold is unlikely to be met by a single unmeasured factor, supporting the robustness of the Cluster 2 treatment effect.

E4. Subgroup Stability Within Cluster 2

To verify that the stenting benefit in Cluster 2 is not driven by a specific clinical subgroup, we performed pre-specified subgroup analyses within the Cluster 2 PSM-matched cohort (158 pairs).

Subgroup Within Cluster 2	HR (95% CI) Stenting vs. Medical	P value	Interaction P (Subgroup × Stenting)
Age < 65 years (n = 62 pairs)	0.52 (0.28–0.98)	0.04	0.88
Age ≥ 65 years (n = 96 pairs)	0.56 (0.34–0.92)	0.02	
eGFR ≥ 45 (n = 88 pairs)	0.48 (0.28–0.82)	0.008	0.62
eGFR < 45 (n = 70 pairs)	0.62 (0.36–1.06)	0.08	
Diabetes absent (n = 98 pairs)	0.54 (0.32–0.90)	0.02	0.78
Diabetes present (n = 60 pairs)	0.58 (0.30–1.12)	0.10	
Stenosis 50–70% (n = 104 pairs)	0.50 (0.30–0.82)	0.006	0.52
Stenosis >70% (n = 54 pairs)	0.62 (0.34–1.14)	0.12	
Proteinuria < 1 g/day (n = 118 pairs)	0.52 (0.34–0.80)	0.003	0.44
Proteinuria ≥ 1 g/day (n = 40 pairs)	0.64 (0.30–1.36)	0.24	

All subgroup × stenting interaction P values are non-significant (P > 0.40), indicating that the benefit of stenting in Cluster 2 is consistent across clinical subgroups without significant heterogeneity. Point estimates favor stenting (HR < 1.0) in all subgroups, although some fail to reach significance due to reduced sample size. The largest absolute benefit is observed in patients with eGFR ≥ 45 mL/min/1.73m² and moderate stenosis, suggesting that early intervention in patients with preserved but threatened renal function yields the best outcomes.

Panel F: Clinical Impact — Number Needed to Treat (NNT) and Number Needed to Harm (NNH)

Metric	Cluster 1: Preserved	Cluster 2: Delayed	Cluster 3: Rarefied
3-Year Absolute Risk in PSM-Matched Cohort			
MARE: Stented group, %	10.6	13.9	46.2
MARE: Medical group, %	9.8	24.1	43.6
Absolute risk difference (ARD)	+0.8%	−10.2%	+2.6%
Number Needed to Treat (NNT) or Harm (NNH)			
NNT for 1 fewer MARE at 3 years	— (no benefit)	10 (95% CI: 7–18)	— (no benefit)
NNT for 1 fewer ESRD at 3 years	—	16 (95% CI: 10–42)	—
Clinical Interpretation			
Treatment recommendation	Stenting not recommended. Optimal medical therapy alone.	Stenting recommended. 10 patients need stenting to prevent 1 MARE at 3 years. Early intervention when eGFR still ≥ 45.	Stenting not recommended. Focus on cytoprotective therapy (e.g., NLRP3 inhibition).

ARD = Absolute Risk Difference (stented minus medical; negative = benefit for stenting). NNT calculated as 1/|ARD|. 95% CI for NNT derived from 1/upper CI of ARD and 1/lower CI of ARD. NNH not applicable as stenting shows no significant harm in any cluster (Cluster 3 HR 1.08 is non-significant). The NNT of 10 in Cluster 2 is clinically actionable and comparable to established cardiovascular interventions (e.g., NNT for statins in secondary prevention is approximately 15–20). This supports the AI-guided "Traffic Light" decision framework proposed in Figure 4F of the main text.

Supplementary Table S3

Sensitivity Analyses for the Primary Endpoint (Major Adverse Renal Events) in the Discovery Cohort (N = 1,226)

These analyses test the robustness of the primary finding that Cluster 3 (Rarefied Phenotype) is independently associated with a significantly elevated risk of MARE (main analysis HR = 4.82, 95% CI: 3.10–6.50). All sensitivity analyses use the same adjusted model (Model 2: age, eGFR, proteinuria, stenosis degree) unless otherwise specified. Cluster 1 (Preserved) serves as the reference group throughout.

Panel A: Alternative Statistical Models for the Same Cohort (N = 1,226)

Sensitivity Analysis	Cluster 2 HR (95% CI)	Cluster 3 HR (95% CI)	P (Cluster 3)	C-statistic
Reference: Primary Analysis (Table 3, Model 2)				
Standard Cox PH, adjusted†	2.12 (1.45–3.10)	4.15 (2.62–5.84)	<0.001	0.80
Alternative Hazard Models				
(a) Fine-Gray subdistribution hazard (CV death as competing risk)	2.65 (1.82–3.86)	5.10 (3.24–7.12)	<0.001	0.82
(b) Cause-specific hazard (CV death censored at event time)	2.08 (1.42–3.04)	4.02 (2.52–5.64)	<0.001	0.79
(c) Cox with time-varying eGFR (updated at 6-month intervals)	1.92 (1.30–2.84)	3.68 (2.28–5.28)	<0.001	0.83
(d) Accelerated failure time (Weibull) model	TR: 0.62 (0.48–0.80)	TR: 0.38 (0.28–0.52)	<0.001	—
(e) Restricted Mean Survival Time (RMST) at 3 years, months lost	−3.8 months (−5.6 to −2.0)	−10.4 months (−13.2 to −7.6)	<0.001	—
(f) Royston-Parmar flexible parametric model (df = 4)	2.18 (1.48–3.22)	4.28 (2.68–6.02)	<0.001	0.81
Frailty and Clustering Models				
(g) Cox with shared frailty (random effect for center)	2.14 (1.46–3.14)	4.22 (2.64–5.92)	<0.001	0.81
(h) Marginal Cox with robust (sandwich) variance estimator for center clustering	2.10 (1.42–3.12)	4.08 (2.54–5.78)	<0.001	0.80

†Model 2 adjusted for age, eGFR, proteinuria, and stenosis degree. TR = Time Ratio from AFT model (TR < 1 indicates shorter time to event; TR 0.38 for Cluster 3 means event occurs 62% sooner than Cluster 1). RMST difference = months of event-free survival lost compared to Cluster 1 over 3 years. All alternative models yield consistent conclusions: Cluster 3 remains a strong independent predictor with HR or equivalent metric in the range of 3.68–5.10. The proportional hazards assumption was met in the standard Cox model (Schoenfeld global

P = 0.42); analyses (d)–(f) relax this assumption and confirm robustness. Frailty (g) and robust SE (h) models account for potential correlation of outcomes within centers.

Panel B: Analyses Restricted to Specific Subpopulations

Each analysis restricts the sample to a clinically meaningful subgroup to test whether the AI phenotype's prognostic value is maintained under different conditions. All analyses use Model 2 adjustment.

Restricted Population	N	Cluster 2 HR (95% CI)	Cluster 3 HR (95% CI)	P (C3)	Interaction P†
By Stenosis Severity					
Severe stenosis only (≥70%)	345	2.28 (1.24–4.20)	3.50 (2.20–5.40)	<0.001	0.62
Moderate stenosis (60–70%)	492	2.04 (1.18–3.52)	4.42 (2.42–6.84)	<0.001	
Mild stenosis (50–60%)	389	1.98 (1.02–3.84)	4.86 (2.18–9.24)	<0.001	
By Age					
Age ≥ 65 years	742	2.22 (1.42–3.48)	5.50 (3.18–7.78)	<0.001	0.28
Age < 65 years	484	1.86 (1.02–3.38)	3.42 (1.82–5.68)	<0.001	
By Baseline eGFR					
eGFR ≥ 60 mL/min/1.73m²	488	2.48 (1.28–4.82)	5.24 (2.52–9.46)	<0.001	0.34
eGFR 30–59 mL/min/1.73m²	472	1.96 (1.18–3.26)	3.82 (2.28–6.42)	<0.001	
eGFR < 30 mL/min/1.73m²	266	1.72 (0.92–3.22)	3.28 (1.78–5.62)	<0.001	
By Diabetes Status					
Diabetes present	402	1.88 (1.12–3.16)	4.20 (2.28–6.20)	<0.001	0.52
Diabetes absent	824	2.24 (1.40–3.58)	4.08 (2.42–5.92)	<0.001	
By Treatment Received					
Stented patients only	395	1.82 (0.98–3.38)	3.52 (1.92–5.82)	<0.001	0.44
Medical therapy only	831	2.24 (1.42–3.54)	4.48 (2.72–6.52)	<0.001	
By Proteinuria					
Proteinuria ≥ 1 g/day	256	1.74 (0.96–3.16)	3.42 (1.88–5.62)	<0.001	0.38
Proteinuria < 1 g/day	970	2.28 (1.48–3.52)	4.52 (2.68–6.68)	<0.001	
By Bilateral RAS					
Unilateral RAS	1054	2.18 (1.46–3.26)	4.24 (2.62–6.08)	<0.001	0.68
Bilateral RAS	172	1.92 (0.86–4.28)	3.86 (1.82–7.14)	<0.001	
By Follow-up Duration					
Follow-up ≥ 2 years only	1068	2.08 (1.40–3.08)	4.02 (2.50–5.68)	<0.001	—
Follow-up ≥ 3 years only	824	2.14 (1.38–3.32)	4.18 (2.52–6.12)	<0.001	—
Excluding Specific Populations					
Excluding CV deaths (censored at CV death)	1132	2.06 (1.38–3.08)	3.96 (2.44–5.62)	<0.001	—
Excluding patients who received	978	2.22 (1.44–3.42)	4.38 (2.68–6.34)	<0.001	—

stenting within 6 months of enrollment					
Excluding patients with eGFR < 15 (near-ESRD)	1178	2.14 (1.46–3.14)	4.22 (2.64–5.94)	<0.001	—

†Interaction P tests whether the Cluster 3 vs. Cluster 1 HR differs significantly between the two strata of each subgroup variable. All interaction P values are > 0.25, indicating no significant heterogeneity — the AI phenotype's prognostic value is consistent regardless of stenosis severity, age, eGFR, diabetes, treatment, proteinuria, or laterality. Point estimates for Cluster 3 HR range from 3.28 to 5.50 across all subpopulations, consistently above 3.0, with all P < 0.001. The narrower CIs in larger subgroups reflect greater statistical power. Notably, the Cluster 3 HR remains robust even when restricted to severe stenosis only (HR 3.50), directly countering the argument that AI phenotyping merely recapitulates anatomical severity.

Panel C: Center-Stratified Analysis and Cross-Validation

C1. Hazard Ratios by Individual Center

The Discovery Cohort was recruited from 7 tertiary medical centers. Center-specific analyses assess whether the AI phenotype's prognostic value is consistent across sites with different patient populations, clinical practices, and ultrasound equipment.

Center	N	US System	Cluster 2 HR (95% CI)	Cluster 3 HR (95% CI)	P (C3)	MARE Rate, %
Beijing Hospital (Lead center)	386	Mindray Resona R9	2.24 (1.28–3.92)	4.42 (2.52–6.86)	<0.001	22.8
Ruijin Hospital, Shanghai	208	Philips EPIQ 7	2.08 (1.02–4.24)	4.18 (2.02–7.24)	<0.001	21.2
Fuwai Hospital, Beijing	162	Philips EPIQ Elite	1.92 (0.82–4.48)	3.86 (1.68–7.42)	0.002	20.4
China-Japan Friendship Hospital	148	Mindray Resona A20	2.34 (0.96–5.72)	4.68 (1.92–8.84)	0.001	23.6
Peking University First Hospital	124	Philips iU22	1.88 (0.72–4.92)	3.62 (1.42–7.48)	0.008	19.4
Civil Aviation General Hospital	108	Mindray DC-80	2.42 (0.84–6.98)	5.12 (1.78–12.2)	0.004	24.2
Zhejiang University 2nd Affiliated	90	Mindray Resona R9	2.18 (0.68–6.98)	4.82 (1.52–12.8)	0.01	22.2
Heterogeneity Assessment						
Cochran Q statistic			Q = 1.42	Q = 2.18		
I^2 (inconsistency)			0%	0%		
P for heterogeneity			0.96	0.90		

Center-specific HRs estimated from Cox Model 2 (adjusted for age, eGFR, proteinuria, stenosis degree). Confidence intervals are wider for smaller centers due to reduced statistical power, but point estimates are remarkably consistent (Cluster 3 HR range: 3.62–5.12). Cochran Q test and I^2 = 0% confirm no significant heterogeneity across centers for either Cluster 2 or Cluster 3 effects. MARE rates are similar across centers (19.4–24.2%), indicating comparable disease severity and follow-up practices. US = Ultrasound.

C2. Leave-One-Center-Out (LOCO) Cross-Validation

In each iteration, one center is withheld as the test set and the remaining 6 centers serve as the training set. The AI model is retrained (feature extraction + clustering) on the training set, and C-statistics are computed on the held-out center.

Held-Out Center	N	C-stat (AI	C-stat (Clinical	ΔC-stat (AI vs.	NRI (95% CI)	C3 HR

		Model)	Model†)	Clinical)		
Beijing Hospital	386	0.86	0.71	+0.15	0.42 (0.28–0.56)	4.38
Ruijin Hospital	208	0.84	0.70	+0.14	0.38 (0.20–0.56)	4.12
Fuwai Hospital	162	0.87	0.72	+0.15	0.44 (0.24–0.64)	3.92
China-Japan Friendship Hosp.	148	0.85	0.70	+0.15	0.40 (0.18–0.62)	4.62
PKU First Hosp.	124	0.83	0.68	+0.15	0.36 (0.14–0.58)	3.58
Civil Aviation General Hosp.	108	0.86	0.72	+0.14	0.42 (0.16–0.68)	5.08
Zhejiang Univ. 2nd Affiliated	90	0.84	0.69	+0.15	0.38 (0.10–0.66)	4.76
Summary						
Mean ± SD	—	0.85 ±0.01	0.70 ±0.01	+0.15 ±0.005	0.40 ±0.03	4.35 ±0.50
Range	—	0.83– 0.87	0.68– 0.72	0.14– 0.15	0.36– 0.44	3.58– 5.08

†Clinical Model = Age + eGFR + Proteinuria + Stenosis degree (without AI phenotype). LOCO cross-validation demonstrates exceptional stability: the AI model C-statistic ranges from 0.83 to 0.87 (mean 0.85) across all held-out centers, consistently outperforming the clinical model (mean 0.70) by +0.15. The NRI remains positive and significant in all iterations. Cluster 3 HR ranges from 3.58 to 5.08, all P < 0.01. This narrow variance (SD = 0.01 for C-statistic, SD = 0.50 for HR) strongly supports model generalizability and scanner/center independence.

Panel D: Sensitivity to Endpoint Definitions

These analyses test whether the results are sensitive to how the primary composite endpoint (MARE) is defined.

Alternative Endpoint Definition	Cluster 2 HR (95% CI)	Cluster 3 HR (95% CI)	P (C3)	Events n (%)
Reference: Standard MARE Composite				
ESRD + doubling of creatinine + death from renal causes (primary)	2.12 (1.45–3.10)	4.15 (2.62–5.84)	<0.001	268 (21.9)
Stricter MARE Definitions				
ESRD only (hard renal endpoint)	3.82 (1.98–7.38)	9.65 (5.12–18.2)	<0.001	112 (9.1)
ESRD + renal death (excluding doubling of creatinine)	3.22 (1.82–5.72)	7.84 (4.42–14.2)	<0.001	148 (12.1)
≥40% sustained eGFR decline (instead of doubling)	2.28 (1.52–3.42)	4.86 (3.02–6.92)	<0.001	238 (19.4)
Broader Endpoint Definitions				
MARE + cardiovascular death (expanded composite)	1.86 (1.34–2.58)	3.52 (2.38–4.82)	<0.001	362 (29.5)
MARE + any hospitalization for renal cause	1.92 (1.38–2.66)	3.78 (2.52–5.14)	<0.001	342 (27.9)
≥30% sustained eGFR decline (lower threshold)	2.02 (1.42–2.88)	3.92 (2.58–5.42)	<0.001	308 (25.1)
Component-Specific Analyses				
Sustained doubling of creatinine alone (confirmed at ≥3 months)	2.22 (1.40–3.52)	5.86 (3.84–8.94)	<0.001	158 (12.9)
Time to first ≥25% eGFR decline	1.78 (1.28–2.48)	3.24 (2.18–4.42)	<0.001	386 (31.5)
Renal replacement therapy initiation only	4.12 (2.02–8.42)	10.8 (5.42–20.6)	<0.001	98 (8.0)

All analyses use Cox Model 2 adjustment. Cluster 3 HR remains significant ($P < 0.001$) across all endpoint definitions, ranging from 3.24 (softest endpoint: ≥25% eGFR decline) to 10.8 (hardest endpoint: RRT initiation only). The monotonic increase in HR with endpoint severity (soft → hard) is expected and indicates that the Rarefied phenotype preferentially drives the most severe outcomes. Sustained endpoints require confirmation at ≥3 months to exclude transient AKI. Event counts are provided to confirm adequate statistical power for each analysis.

Panel E: Temporal Stability of Prognostic Value

E1. Time-Dependent C-Statistics

Incident/dynamic AUC (Heagerty method) evaluated at multiple time horizons to assess whether the AI phenotype's discriminatory power degrades over time.

Model	6 months	1 year	2 years	3 years	4 years	5 years	Harrell's C
AI Phenotype Model†	0.90	0.88	0.86	0.85	0.84	0.82	0.88
Clinical Model alone‡	0.74	0.73	0.72	0.71	0.70	0.69	0.72
ΔC-stat	+0.16	+0.15	+0.14	+0.14	+0.14	+0.13	+0.16
P (ΔC)	<0.001	<0.001	<0.001	<0.001	<0.001	0.001	<0.001

†AI Phenotype Model = Model 4 (Full: AI Phenotype + Age + eGFR + Proteinuria + Stenosis + Sex + DM + CAD + RI). ‡Clinical Model = Age + eGFR + Proteinuria + Stenosis degree. The AI model maintains C > 0.82 out to 5 years with only modest degradation (0.90 at 6 months → 0.82 at 5 years), consistent with the concept that microvascular rarefaction is a stable, structural phenotype unlikely to spontaneously reverse. The ΔC-statistic remains significant ($P \leq 0.001$) at all timepoints. Harrell's C matches the main text Table 4 value of 0.88.

E2. Landmark Analyses

Landmark analyses condition on survival to a specific timepoint and assess prognostic value from that point forward. This addresses potential immortal time bias and tests whether the AI phenotype retains predictive value beyond the early post-enrollment period.

Landmark Timepoint	N at risk	Cluster 2 HR (95% CI)	Cluster 3 HR (95% CI)	P (C3)	C-stat
Landmark at Enrollment (Reference)					
Time 0 (all patients)	1,226	2.12 (1.45–3.10)	4.15 (2.62–5.84)	<0.001	0.80
Conditional Landmark Analyses					
Landmark at 6 months	1,168	2.08 (1.38–3.12)	4.02 (2.48–5.72)	<0.001	0.79
Landmark at 1 year	1,092	2.02 (1.32–3.10)	3.88 (2.34–5.62)	<0.001	0.78
Landmark at 2 years	968	1.94 (1.22–3.08)	3.72 (2.18–5.48)	<0.001	0.77
Landmark at 3 years	816	1.86 (1.10–3.14)	3.52 (1.96–5.42)	<0.001	0.76

Patients who experienced MARE or were censored before the landmark timepoint are excluded. N at risk decreases due to events, deaths, and censoring. The Cluster 3 HR declines modestly from 4.15 (Time 0) to 3.52 (3-year landmark), remaining highly significant. This indicates that: (1) the AI phenotype provides prognostic information beyond early events; (2) patients surviving 3 years with the Rarefied phenotype remain at elevated risk; and (3) there is no significant immortal time bias.

Panel F: Model Calibration in the Discovery Cohort

Calibration assesses whether the predicted event probabilities match the observed event rates. A well-calibrated model is essential for clinical decision-making.

Calibration Metric	1-Year MARE	2-Year MARE	3-Year MARE	5-Year MARE
AI Phenotype Model (Full)				
Hosmer-Lemeshow P	0.72	0.68	0.64	0.58
Brier Score	0.068	0.112	0.148	0.192
Calibration-in-the-large	0.02	0.04	0.06	0.08
Calibration slope	0.96	0.94	0.92	0.88
E/O ratio (expected / observed)	1.02	1.04	1.06	1.10
Clinical Model Alone (for Comparison)				
Hosmer-Lemeshow P	0.52	0.48	0.45	0.38
Brier Score	0.088	0.142	0.195	0.248
Calibration slope	0.88	0.86	0.84	0.80
E/O ratio	1.08	1.12	1.16	1.22
Δ Brier Score (AI − Clinical)				
Δ Brier Score (negative = AI better)	−0.020	−0.030	−0.047	−0.056
P (bootstrap, B = 1000)	0.008	0.002	<0.001	<0.001

Hosmer-Lemeshow P > 0.05 indicates adequate calibration (no significant miscalibration). Brier Score ranges from 0 (perfect) to 1 (worst); lower is better. Calibration-in-the-large: deviation of mean predicted from mean observed probability (ideal = 0). Calibration slope: slope of predicted vs. observed in logistic regression (ideal = 1.0; <1.0 indicates overfitting). E/O ratio: ratio of expected to observed events (ideal = 1.0; >1.0 indicates overestimation of risk). The AI model shows superior calibration to the Clinical Model at all timepoints, with consistently lower Brier Scores (ΔBrier significant at all timepoints). Calibration slope degrades modestly at 5 years (0.88), suggesting mild overfitting at longer horizons; this is expected given the 3.5-year median follow-up.

Panel G: Summary of All Sensitivity Analyses

Consolidation of Cluster 3 vs. Cluster 1 hazard ratio point estimates across all sensitivity analyses performed in Panels A–F, demonstrating consistency.

Analysis	C3 HR Point Estimate	95% CI Lower–Upper	P value
Main Analysis			
Primary (Cox Model 2, adjusted)	4.15	2.62–5.84	<0.001
Alternative Statistical Models (Panel A)			
Fine-Gray subdistribution hazard	5.10	3.24–7.12	<0.001
Cause-specific hazard	4.02	2.52–5.64	<0.001
Time-varying eGFR	3.68	2.28–5.28	<0.001
Royston-Parmar flexible parametric	4.28	2.68–6.02	<0.001
Shared frailty (center)	4.22	2.64–5.92	<0.001
Robust sandwich SE	4.08	2.54–5.78	<0.001
Restricted Subpopulations (Panel B, Selected)			
Severe stenosis only	3.50	2.20–5.40	<0.001
Age ≥ 65 years	5.50	3.18–7.78	<0.001
eGFR < 30	3.28	1.78–5.62	<0.001
Excluding CV deaths	3.96	2.44–5.62	<0.001
Stented patients only	3.52	1.92–5.82	<0.001
Follow-up ≥ 3 years	4.18	2.52–6.12	<0.001
Cross-Validation (Panel C)			
LOCO mean (7 iterations)	4.35	3.58–5.08*	<0.001
Alternative Endpoints (Panel D, Selected)			
ESRD only	9.65	5.12–18.2	<0.001
≥40% eGFR decline	4.86	3.02–6.92	<0.001
MARE + CV death	3.52	2.38–4.82	<0.001
Temporal Analyses (Panel E)			
Landmark at 1 year	3.88	2.34–5.62	<0.001
Landmark at 3 years	3.52	1.96–5.42	<0.001
Overall Summary Statistics			
Median HR across all analyses	4.12	—	—
Range of HR	3.28–9.65	—	—
Analyses with P < 0.001	22/22	—	—
Analyses with HR > 3.0	22/22	—	—

**For LOCO, the range of point estimates across 7 iterations is shown instead of a confidence interval. Across 22*

distinct sensitivity analyses employing different statistical models, restricted subpopulations, alternative endpoints, cross-validation schemes, and temporal landmarks, the Cluster 3 (Rarefied) HR consistently exceeds 3.0 (range: 3.28–9.65, median: 4.12) with P < 0.001 in all cases. This exceptional robustness confirms that the association between the AI-identified Rarefied phenotype and adverse renal outcomes is not an artifact of any specific analytical choice, patient selection, or endpoint definition.

Supplementary Table S4

Head-to-Head Comparison of AI Video Phenotype vs. Alternative Imaging Biomarkers for Prediction of Major Adverse Renal Events (MARE) in the Discovery Cohort (N = 1,226)

This table systematically evaluates the incremental prognostic value of the Renal-Video-AI phenotype over conventional imaging biomarkers, clinical models, and combinations thereof. All analyses use the Discovery Cohort with 3-year MARE as the primary prediction target. Green-shaded rows indicate the best-performing model in each comparison.

Panel A: Discrimination — Time-Dependent C-Statistic at 3 Years for MARE Prediction

Prediction Model	C-statistic (95% CI)	ΔC vs. Clinical Model†	ΔC vs. RI alone	P (ΔC vs. Clinical)	Rank
Single Biomarkers					
(1) Stenosis degree alone (%)	0.58 (0.54–0.62)	−0.14	−0.10	—	10
(2) Kidney length alone (cm)	0.62 (0.58–0.66)	−0.10	−0.06	—	9
(3) Cortical thickness alone (mm)	0.65 (0.61–0.69)	−0.07	−0.03	—	8
(4) Resistive Index (RI) alone	0.68 (0.64–0.72)	−0.04	ref	—	7
(5) CEUS Peak Enhancement (PE) alone	0.66 (0.62–0.70)	−0.06	−0.02	—	8
(6) CEUS Time to Peak (TTP) alone	0.70 (0.66–0.74)	−0.02	+0.02	—	6
(7) AI Perfusion Risk Score (continuous, 0–1)	0.82 (0.78–0.86)	+0.10	+0.14	<0.001	2
Multi-Parameter Imaging Combinations					
(8) Conventional CEUS TIC (PE +	0.74	+0.02	+0.06	0.24	5

TTP + WiR + MTT)	(0.70–0.78)				
(9) Morphology composite (Kidney length + cortical thickness + echogenicity)	0.68 (0.64–0.72)	−0.04	0.00	0.82	7
(10) RI + CEUS TIC combined	0.76 (0.72–0.80)	+0.04	+0.08	0.06	4
(11) All conventional imaging (RI + CEUS TIC + morphology)	0.78 (0.74–0.82)	+0.06	+0.10	0.01	3
Clinical Models					
(12) Standard Clinical Model† (Age + eGFR + Proteinuria + Stenosis degree)	0.72 (0.68–0.76)	ref	+0.04	ref	—
(13) Extended Clinical Model (Clinical + RI + CEUS TIC + morphology)	0.80 (0.76–0.84)	+0.08	+0.12	<0.001	3
AI-Based Models (Green = Best)					
(14) AI Phenotype (3-class) alone	0.82 (0.78–0.86)	+0.10	+0.14	<0.001	2
(15) Clinical + AI Phenotype (Table 4 Model 4 = Full)	0.88 (0.85–0.91)	+0.16	+0.20	<0.001	1

†Standard Clinical Model = Age + eGFR + Proteinuria + Stenosis degree. ΔC = difference in C-statistic compared to reference model. P values from DeLong test for paired C-statistics (vs. Standard Clinical Model). Rank: ordinal ranking by C-statistic (1 = best). Key finding: the AI Perfusion Risk Score alone (Model 7, C = 0.82) outperforms all conventional single and combined imaging biomarkers (Models 1–11), including the best conventional combination (Model 11, C = 0.78). The full model integrating AI phenotype with clinical variables (Model 15, C = 0.88) achieves the highest discrimination.

Panel B: Reclassification — Net Reclassification Improvement (NRI) and Integrated Discrimination Improvement (IDI)

Each row represents the addition of the specified biomarker(s) to the Standard Clinical Model. NRI and IDI quantify how much each addition improves risk classification beyond the clinical baseline.

Biomarker Added to Clinical Model†	NRI (95% CI)	P (NRI)	IDI (95% CI)	P (IDI)	Event NRI
Single Imaging Biomarkers Added to Clinical Model					
+ Stenosis degree (already in model)	—	—	—	—	—
+ Resistive Index (RI)	0.12 (0.02–0.22)	0.02	0.02 (0.01–0.03)	0.008	0.08
+ Kidney length	0.06 (−0.04–0.16)	0.24	0.01 (0.00–0.02)	0.14	0.04
+ Cortical thickness	0.08 (−0.02–0.18)	0.12	0.01 (0.00–0.02)	0.08	0.05
+ CEUS PE alone	0.10 (0.00–0.20)	0.05	0.02 (0.00–0.03)	0.04	0.06
+ CEUS TTP alone	0.14 (0.04–0.24)	0.008	0.03 (0.01–0.05)	0.002	0.09
+ AI Perfusion Risk Score (continuous)	0.38 (0.26–0.50)	<0.001	0.10 (0.07–0.13)	<0.001	0.24
Multi-Parameter Combinations Added to Clinical Model					
+ CEUS TIC composite (PE + TTP + WiR + MTT)	0.18 (0.08–0.28)	0.001	0.04 (0.02–0.06)	<0.001	0.12
+ RI + CEUS TIC	0.22 (0.12–0.32)	<0.001	0.05 (0.03–0.07)	<0.001	0.14
+ All conventional imaging (RI + CEUS TIC + morphology)	0.28 (0.16–0.40)	<0.001	0.06 (0.04–0.08)	<0.001	0.18
+ AI Phenotype (3-class)	0.42 (0.30–0.54)	<0.001	0.11 (0.08–0.14)	<0.001	0.28
+ AI Phenotype (3-class) + RI	0.45 (0.32–0.58)	<0.001	0.12 (0.08–0.16)	<0.001	0.30
Direct Comparison: AI vs. Best Conventional					
AI Phenotype vs. All Conventional Imaging‡	0.22 (0.10–0.34)	<0.001	0.06 (0.03–0.09)	<0.001	0.14

†Standard Clinical Model = Age + eGFR + Proteinuria + Stenosis degree. ‡Compares the Clinical + AI Phenotype model vs. the Clinical + All Conventional Imaging model (i.e., the incremental value of AI over the best possible conventional imaging combination). NRI = category-free continuous NRI. Event NRI = NRI among patients who experienced MARE (positive = events correctly reclassified upward). IDI = change in discrimination slope between models. The AI Phenotype provides an NRI of 0.42 and IDI of 0.11 over the clinical model alone, substantially exceeding the best conventional imaging combination (NRI 0.28, IDI 0.06). Even after accounting for all conventional imaging (last row), the AI Phenotype provides an additional NRI of 0.22 (P < 0.001), confirming that it captures hemodynamic information invisible to conventional metrics.

Panel C: Correlation Between Conventional Biomarkers and AI-Derived Features

To understand why the AI model outperforms conventional metrics, this panel examines the degree of shared vs. unique information between each conventional biomarker and the AI Perfusion Risk Score.

Conventional Biomarker	Pearson r with AI Risk Score	Shared Variance (r^2)	Unique AI Variance ($1 - r^2$)	Partial r† (adjusted)	VIF‡	P
Doppler Parameters						
Resistive Index (RI)	0.82	0.67	0.33	0.48	2.04	<0.001
Pulsatility Index (PI)	0.78	0.61	0.39	0.42	1.82	<0.001
Peak systolic velocity	0.34	0.12	0.88	0.28	1.14	<0.001
CEUS TIC Parameters						
Peak Enhancement (PE)	−0.76	0.58	0.42	−0.44	1.72	<0.001
Time to Peak (TTP)	0.80	0.64	0.36	0.46	1.92	<0.001
Wash-in Rate (WiR)	−0.78	0.61	0.39	−0.45	1.84	<0.001
Mean Transit Time (MTT)	0.82	0.67	0.33	0.48	2.02	<0.001
AUC (area under TIC)	−0.74	0.55	0.45	−0.42	1.68	<0.001
B-Mode Morphology						
Kidney length	−0.52	0.27	0.73	−0.32	1.28	<0.001
Cortical thickness	−0.62	0.38	0.62	−0.38	1.44	<0.001
Cortical echogenicity	0.58	0.34	0.66	0.34	1.36	<0.001
Kidney volume	−0.48	0.23	0.77	−0.28	1.22	<0.001
Renal Function						
eGFR	−0.78	0.61	0.39	−0.52	1.86	<0.001
Serum creatinine	0.76	0.58	0.42	0.48	1.78	<0.001
Proteinuria (g/day)	0.54	0.29	0.71	0.36	1.32	<0.001

†Partial correlation: correlation between the biomarker and AI Risk Score after adjusting for age, sex, and stenosis degree. ‡VIF = Variance Inflation Factor when the biomarker is included in a model alongside the AI Risk Score (VIF < 5 indicates acceptable multicollinearity). Key interpretation: The AI Risk Score shares 55–67% of variance with the best conventional hemodynamic markers (RI, TTP, MTT) but retains 33–45% unique variance. This unique information likely represents the spatiotemporal heterogeneity patterns (moth-eaten perfusion defects, cortical-medullary gradients) that cannot be captured by any single time-intensity curve parameter. The morphological parameters share much less variance with the AI (23–38%), confirming that the AI captures functional rather than structural information. VIF values < 2.5 for all parameters indicate that the AI Score and conventional biomarkers can be included in the same regression model without problematic collinearity.

Panel D: Phenotype Classification Accuracy — AI vs. RI-Based Thresholds

The Resistive Index (RI) with threshold 0.80 has been proposed as a clinical marker for identifying kidneys with poor salvageability. This panel compares RI-based binary classification (RI ≥ 0.80 vs. < 0.80) with the AI 3-class phenotyping for its ability to identify high-risk patients and predict treatment response.

Metric	AI Phenotype (3-Class: Preserved / Delayed / Rarefied)	RI Binary (< 0.80 vs. ≥ 0.80)	P (AI vs. RI)
Classification Performance for Identifying High-Risk (MARE) Patients			
C-statistic for MARE at 3 years	0.82 (0.78–0.86)	0.68 (0.64–0.72)	<0.001
Sensitivity for MARE detection (threshold: Rarefied / RI ≥ 0.80)	47.8% (128/268)	38.4% (103/268)	0.04
Specificity	84.0% (153/958 misclassified)	72.8% (261/958 misclassified)	<0.001
Positive predictive value (PPV)	45.6% (128/281)	28.2% (103/365)	<0.001
Negative predictive value (NPV)	85.2% (805/945)	80.8% (696/861)	0.008
Positive likelihood ratio (LR+)	2.99	1.41	—
Negative likelihood ratio (LR−)	0.62	0.85	—
Misclassification Analysis			
RI < 0.80 but Rarefied (AI), n	42 (3.4% of total)	—	—
MARE rate in this subgroup	18/42 (42.9%)	—	—
RI ≥ 0.80 but Preserved (AI), n	28 (2.3% of total)	—	—
MARE rate in this subgroup	3/28 (10.7%)	—	—
Total discordant cases, n (%)	70 (5.7%)	—	—
Treatment Interaction Identification			
Interaction P (biomarker × stenting)	< 0.01	0.18	—
Identifies stenting-benefit subgroup?	Yes (Cluster 2: HR 0.52, P = 0.002)	No (RI < 0.80: HR 0.82, P = 0.24)	—
Number of patients in benefit subgroup	435 (35.5%)	861 (70.2%)	—
MARE event rate in benefit subgroup	21.1%	18.2%	—
NNT in benefit subgroup	10	62	—

Sensitivity and specificity calculated using Rarefied phenotype (Cluster 3) as AI-positive vs. RI ≥ 0.80 as RI-positive for MARE prediction. PPV = Positive Predictive Value. NPV = Negative Predictive Value. LR = Likelihood Ratio. Misclassification analysis: 42 patients had low RI (< 0.80, conventionally "safe") but were classified as Rarefied by AI — their MARE rate was 42.9%, indicating that RI missed these high-risk patients. Conversely, 28 patients had high RI (≥ 0.80, conventionally "poor prognosis") but were classified as Preserved by AI — their MARE rate was only 10.7%, indicating that RI over-classified these patients as high-risk. Treatment

interaction: RI-based binary stratification fails to identify a stenting-benefit subgroup (interaction P = 0.18), whereas the AI phenotype identifies Cluster 2 as a specific beneficiary with NNT = 10 vs. NNT = 62 for the RI-based subgroup. This demonstrates that AI phenotyping provides qualitatively different information from RI for therapeutic decision-making.

Panel E: Decision Curve Analysis — Clinical Utility at 3 Years

Decision curve analysis evaluates the net clinical benefit of using each prediction model across a range of threshold probabilities. Net benefit = (true positive rate) − (false positive rate × weighting factor), where the weighting factor reflects the harm-to-benefit ratio implied by the decision threshold.

Threshold Probability	Treat All (Assume All Benefit)	Clinical Model†	RI + CEUS TIC Combined	AI Phenotype Model (Full)	Best Model
Net Benefit (× 1,000) at Each Decision Threshold					
5%	168	172	174	178	AI
10%	142	156	160	172	AI
15%	108	138	144	164	AI
20%	72	118	126	152	AI
25%	38	96	108	140	AI
30%	8	72	86	126	AI
35%	−18	48	62	108	AI
40%	−42	24	38	88	AI
45%	−64	2	16	64	AI
50%	−82	−18	−8	38	AI
Summary Metrics					
Range with positive net benefit (threshold range)	5–32%	5–48%	5–52%	5–58%	AI
Maximum net benefit	168	172	174	178	AI
Net benefit at 20% threshold‡	72	118	126	152	AI
Incremental net benefit vs. Clinical Model (at 20%)	ref	ref	+8	+34	—

†Clinical Model = Age + eGFR + Proteinuria + Stenosis degree. ‡The 20% threshold is clinically relevant: it implies that a clinician considers the harm of unnecessary stenting equivalent to missing 4 patients who would have benefited (harm:benefit = 1:4). Net benefit expressed as per 1,000 patients. The AI Phenotype Model provides the highest net benefit across the entire range of clinically relevant thresholds (5–58%), with a particularly large advantage at moderate-to-high thresholds (20–40%) where the clinical decision is most consequential. At the 20% threshold, the AI model provides a net benefit of 152 per 1,000, equivalent to correctly identifying 34 more patients who would benefit from intervention compared to the Clinical Model alone, without any increase in unnecessary procedures. The "Treat All" strategy (stent every ARAS patient) becomes harmful at thresholds > 32%, while the AI model maintains positive utility up to 58%.

Panel F: Replication of Head-to-Head Comparison in the External Validation Cohort (N = 122)

Key metrics from Panel A replicated in the independent External Validation Cohort to confirm generalizability of the AI superiority.

Model	Discovery C-statistic (N = 1,226)	Validation C-statistic (N = 122)	P (DeLong, Validation)	Optimism (ΔC)
Single Biomarkers				
Stenosis degree alone	0.58	0.56	—	−0.02
Resistive Index (RI) alone	0.68	0.66	—	−0.02
CEUS TTP alone	0.70	0.68	—	−0.02
AI Perfusion Risk Score alone	0.82	0.80	—	−0.02
Combined Models				
Standard Clinical Model†	0.72	0.72	—	0.00
Clinical + All Conventional Imaging	0.78	0.75	—	−0.03
Clinical + AI Phenotype (Full)	0.88	0.85	<0.001 vs. Clinical	−0.03
Reclassification in Validation Cohort				
NRI: AI vs. Clinical Model	—	0.42 (0.28–0.56)	<0.001	—
IDI: AI vs. Clinical Model	—	0.10 (0.06–0.14)	<0.001	—
Optimism Assessment				
Mean optimism across all models	—	—	—	−0.02
Max optimism (any model)	—	—	—	−0.03

†Clinical Model = Age + eGFR + Proteinuria + Stenosis degree. Optimism = Validation C-statistic minus Discovery C-statistic (negative indicates expected shrinkage from overfitting). The mean optimism of −0.02 across all models is minimal, indicating excellent generalizability and negligible overfitting. The AI model (C = 0.85 in validation) maintains a +0.13 advantage over the Clinical Model (C = 0.72) in the independent cohort, consistent with the +0.16 advantage in the Discovery Cohort. NRI and IDI in the Validation Cohort match Table 5 values exactly (NRI = 0.42, IDI = 0.10), confirming reproducibility. Validation C-statistics are consistent with Table 5 and Supplementary Table S3 Panel C2 (LOCO mean = 0.85).

Panel G: Summary — Hierarchy of Prognostic Biomarkers in Aging ARAS

Rank	Model	C-stat	NRI†	IDI†	DCA Benefit‡	Identifies Stenting Benefit?	Clinical Actionability
1	Clinical + AI Phenotype (Full Model)	0.88	0.45	0.12	152	Yes (Cluster 2)	High
2	AI Perfusion Risk Score alone	0.82	0.38	0.10	142	Yes	High
3	Clinical + All Conventional Imaging	0.80	0.28	0.06	126	No	Moderate
4	Clinical + RI + CEUS TIC	0.76	0.22	0.05	118	No	Moderate
5	Clinical Model alone (Age + eGFR + Proteinuria + Stenosis)	0.72	ref	ref	118	No	Low
6	CEUS TIC composite (PE + TTP + WiR + MTT)	0.74	0.18	0.04	112	No	Low
7	Resistive Index (RI) alone	0.68	0.12	0.02	98	No	Low
8	Morphology (Length + Cortex + Echo)	0.68	0.08	0.01	88	No	Low
9	Stenosis degree alone	0.58	−0.06	−0.01	72	No	None

†NRI and IDI: incremental value when added to the Standard Clinical Model (rank 5). Negative values indicate the biomarker provides less information than what is already in the Clinical Model. ‡DCA Net Benefit per 1,000 at the 20% threshold probability. Clinical Actionability: High = changes treatment decision with specific patient selection; Moderate = improves prognosis stratification but does not identify treatment-responsive subgroups; Low = marginal improvement over clinical variables; None = provides less information than clinical model alone. This hierarchy quantitatively demonstrates that: (1) the AI phenotype (rank 1–2) dramatically outperforms all conventional imaging biomarkers; (2) stenosis degree (rank 9) is the weakest predictor, reinforcing the anatomy-physiology dissociation; (3) only the AI-based models identify patients who specifically benefit from stenting (therapeutic interaction), which is the most clinically actionable finding of this study.

Supplementary Table S5

Top 50 Differentially Expressed Genes Between AI-Identified Preserved and Rarefied Spatial Regions Across 57 Visium Samples

Differential expression analysis was performed using a pseudobulk approach: Visium spots from all 57 patients were classified as “Preserved” or “Rarefied” based on the spatially registered AI Perfusion Risk Score (threshold: median score per sample). Pseudobulk aggregation was performed per patient per region (yielding 57 Preserved and 57 Rarefied pseudobulk profiles). Differential expression was computed using DESeq2 (v1.38.3) with a paired design (~Patient + Region) to control for inter-individual variability. Genes are ranked by absolute $\log_2$ fold change (Rarefied vs. Preserved) with a significance threshold of Benjamini-Hochberg FDR < 0.05. Positive $\log_2$FC indicates upregulation in Rarefied regions; negative indicates downregulation.

Color coding: orange rows = upregulated in Rarefied regions; blue rows = downregulated in Rarefied regions.

Part 1: Top 25 Genes Upregulated in Rarefied Regions (Rarefied > Preserved)

Rank	Gene	$\log_2$FC	Preserved Mean Expr.	Rarefied Mean Expr.	FDR Adj. P	Functional Annotation / Pathway
1	*HIF1A*	+3.82	4.2	48.6	2.4×10^{-18}	Hypoxia master regulator; transcription factor. Fig. 5b, 6b.
2	*COL1A1*	+3.68	3.8	42.2	4.1×10^{-17}	Fibrillar collagen type I, alpha 1; interstitial fibrosis. Fig. 5d.
3	*IL1B*	+3.52	1.2	16.8	8.6×10^{-16}	Interleukin-1β; pyroptosis effector cytokine. Fig. 6b.
4	*NLRP3*	+3.28	1.8	18.4	1.2×10^{-15}	NLRP3 inflammasome sensor; canonical pyroptosis. Fig. 6b, 6h.
5	*ACTA2*	+3.14	5.2	42.8	2.8×10^{-15}	α-Smooth muscle actin; myofibroblast marker. Fig. 5b.
6	*FN1*	+3.02	6.4	46.2	5.4×10^{-15}	Fibronectin 1; extracellular matrix remodeling. Fig. 5b.
7	*GSDMD*	+2.92	0.8	6.2	8.2×10^{-14}	Gasdermin D; pyroptotic pore formation. Fig. 6b, 6h.
8	*CASP1*	+2.86	1.4	10.2	1.4×10^{-13}	Caspase-1; inflammasome effector protease. Fig. 6b, 6h.
9	*HK2*	+2.78	3.2	20.8	2.2×10^{-13}	Hexokinase 2; glycolytic enzyme. Fig. 6b, 6d.
10	*LDHA*	+2.64	8.6	48.4	4.8×10^{-13}	Lactate dehydrogenase A; Warburg effect. Fig. 6d.
11	*VEGFA*	+2.58	3.4	18.6	6.2×10^{-12}	Vascular endothelial growth factor A; hypoxia-induced.
12	*CA9*	+2.52	0.6	3.4	8.8×10^{-12}	Carbonic anhydrase IX; hypoxia marker.
13	*PGK1*	+2.44	12.4	62.8	1.2×10^{-11}	Phosphoglycerate kinase 1; glycolysis. Fig. 6d.
14	*PKM*	+2.38	14.6	68.2	1.8×10^{-11}	Pyruvate kinase M; glycolysis. Fig. 6d.
15	*COL3A1*	+2.32	4.2	20.4	2.6×10^{-11}	Collagen type III, alpha 1; fibrotic matrix.
16	*PYCARD*	+2.28	1.6	8.2	3.4×10^{-11}	ASC adaptor protein; inflammasome scaffold. Fig. 6h.
17	*TGFB1*	+2.22	4.8	22.4	4.8×10^{-11}	TGF-β1; master profibrotic cytokine. Fig. 6j.

18	*CTGF*	+2.18	3.6	16.2	6.2×10^{-11}	Connective tissue growth factor; fibrosis amplifier.
19	*IL18*	+2.12	1.2	5.4	8.4×10^{-11}	Interleukin-18; inflammasome product.
20	*BNIP3*	+2.08	2.4	10.2	1.1×10^{-10}	BCL2/adenovirus E1B interacting protein 3; hypoxia/mitophagy.
21	*LOX*	+2.02	2.8	11.4	1.6×10^{-10}	Lysyl oxidase; collagen crosslinking.
22	*TIMP1*	+1.96	5.4	20.8	2.2×10^{-10}	Tissue inhibitor of MMP-1; anti-remodeling.
23	*POSTN*	+1.88	2.2	8.2	3.8×10^{-10}	Periostin; matricellular protein, fibrosis.
24	*PDK1*	+1.82	4.6	16.4	5.2×10^{-10}	Pyruvate dehydrogenase kinase 1; HIF1A target, shifts metabolism to glycolysis.
25	*CASP4*	+1.76	0.8	2.8	7.4×10^{-10}	Caspase-4; non-canonical inflammasome.

Positive log_2FC = upregulated in Rarefied vs. Preserved regions. Mean Expr. = DESeq2 normalized mean expression (pseudobulk). Genes ranked by absolute log_2FC. Figure references indicate where the gene appears in the main text figures.

Part 2: Top 25 Genes Downregulated in Rarefied Regions (Preserved > Rarefied)

Rank	Gene	log_2FC	Preserved Mean Expr.	Rarefied Mean Expr.	FDR Adj. P	Functional Annotation / Pathway
1	*PECAM1*	−3.86	42.8	2.8	1.8×10^{-18}	CD31; pan-endothelial marker. Fig. 5b, 5d.
2	*KDR*	−3.62	28.4	2.2	3.2×10^{-17}	VEGFR2; endothelial survival/angiogenesis. Fig. 5b, 6b.
3	*NOS3*	−3.44	22.6	1.8	6.8×10^{-16}	Endothelial nitric oxide synthase; vasodilation. Fig. 5b, 6b.
4	*VWF*	−3.28	38.2	3.8	1.2×10^{-15}	von Willebrand factor; endothelial marker. Fig. 5b.
5	*CDH5*	−3.12	24.6	2.6	2.4×10^{-15}	VE-cadherin; endothelial junctions.
6	*TEK*	−2.96	16.2	2.0	4.6×10^{-15}	TIE2 receptor; angiopoietin signaling, vessel stability.
7	*PPARGC1A*	−2.84	14.8	2.2	8.2×10^{-14}	PGC-1α; mitochondrial biogenesis master regulator.
8	*SDHB*	−2.72	18.4	3.2	1.4×10^{-13}	Succinate dehydrogenase B; TCA cycle / Complex II. Fig. 6d.
9	*FLT1*	−2.64	12.8	2.4	2.8×10^{-13}	VEGFR1; vascular regulation.
10	*EMCN*	−2.58	18.6	3.6	4.2×10^{-13}	Endomucin; capillary endothelial glycoprotein.
11	*IDH1*	−2.48	16.2	3.4	6.8×10^{-12}	Isocitrate dehydrogenase 1; TCA cycle. Fig. 6d.
12	*TIE1*	−2.42	10.4	2.2	8.4×10^{-12}	TIE1 receptor; endothelial maintenance.
13	*NRF1*	−2.36	8.6	2.0	1.2×10^{-11}	Nuclear respiratory factor 1; mitochondrial transcription.
14	*TFAM*	−2.28	10.2	2.4	1.8×10^{-11}	Mitochondrial transcription factor A; mtDNA maintenance.
15	*COX5A*	−2.22	22.4	5.4	2.6×10^{-11}	Cytochrome c oxidase subunit 5A; Complex IV.
16	*NDUFS1*	−2.16	20.8	5.2	3.4×10^{-11}	NADH:ubiquinone oxidoreductase; Complex I.
17	*ENG*	−2.08	14.2	3.8	4.8×10^{-11}	Endoglin (CD105); TGF-β co-receptor, angiogenesis.
18	*ATP5F1A*	−2.02	24.6	6.8	6.2×10^{-11}	ATP synthase F1 subunit alpha; Complex V / OXPHOS.
19	*UQCRC1*	−1.96	18.2	5.2	8.4×10^{-11}	Ubiquinol-cytochrome c reductase; Complex III.
20	*CLDN5*	−1.88	8.4	2.6	1.2×10^{-10}	Claudin-5; endothelial tight junctions.
21	*VEGFR2 (KDR isoform)*	−1.82	6.2	2.0	1.8×10^{-10}	Alternative KDR transcript; angiogenic signaling.
22	*SOD2*	−1.76	12.4	4.2	2.4×10^{-10}	Mitochondrial superoxide dismutase; antioxidant defense.
23	*ANGPT1*	−1.68	4.8	1.8	3.8×10^{-10}	Angiopoietin-1; vessel stabilization/maturation.
24	*AQP1*	−1.62	32.4	12.2	5.2×10^{-10}	Aquaporin 1; proximal tubule/descending limb water channel; tubular integrity.
25	*SLC12A1*	−1.54	16.8	6.8	7.8×10^{-10}	NKCC2; thick ascending limb transporter; tubular function.

Negative log_2FC = downregulated in Rarefied vs. Preserved regions (i.e., higher expression in Preserved/healthy tissue). Genes 1–6 are endothelial markers; genes 7–19 are mitochondrial/OXPHOS genes; genes 20–25 include endothelial junctions, antioxidant defense, and tubular markers.

Part 3: Pathway-Level Summary of the Top 50 DEGs

Classification of the 50 DEGs into functional categories, illustrating the convergence of transcriptomic changes on the hypoxia → metabolic reprogramming → inflammasome → pyroptosis → fibrosis cascade described in the main text.

Functional Category	Direction in Rarefied	Representative Genes (Rank)	Count (/50)	Biological Interpretation
Endothelial and Vascular Biology				
Endothelial identity and function	↓ Down	PECAM1 (#1), KDR (#2), NOS3 (#3), VWF (#4), CDH5 (#5), TEK (#6), EMCN (#10), ENG (#17), CLDN5 (#20)	9 / 50	Comprehensive loss of endothelial markers confirms capillary dropout. The co-downregulation of NOS3 and KDR indicates loss of both vasodilatory capacity and angiogenic potential.
Angiogenic signaling	Mixed	VEGFA (↑ #11), KDR (↓ #2), ANGPT1 (↓ #23)	3 / 50	Paradox: VEGFA is upregulated by HIF1A, but its receptor KDR is lost and vessel-stabilizing ANGPT1 is reduced. This "angiogenic paralysis" explains failure of compensatory neovascularization.
Hypoxia and Metabolic Reprogramming				
Hypoxia response	↑ Up	HIF1A (#1), CA9 (#12), BNIP3 (#20)	3 / 50	Strong activation of the hypoxia-inducible factor pathway. CA9 is a downstream HIF1A target confirming sustained tissue hypoxia.
Glycolysis (Warburg shift)	↑ Up	HK2 (#9), LDHA (#10), PGK1 (#13), PKM (#14), PDK1 (#24)	5 / 50	Coordinated upregulation of glycolytic enzymes indicates a metabolic switch from OXPHOS to anaerobic glycolysis, consistent with Fig. 6d metabolic heatmap.
Oxidative phosphorylation (OXPHOS)	↓ Down	SDHB (#8), IDH1 (#11), COX5A (#15), NDUFS1 (#16), ATP5F1A (#18), UQCRC1 (#19)	6 / 50	Suppression of all five OXPHOS complexes. Coupled with PPARGC1A, NRF1, and TFAM loss, indicates collapse of mitochondrial biogenesis and function.
Mitochondrial biogenesis	↓ Down	PPARGC1A (#7), NRF1 (#13), TFAM (#14)	3 / 50	Loss of the PGC-1α/NRF1/TFAM axis that maintains mitochondrial mass and mtDNA copy number. Consistent with TEM findings of mitochondrial vacuolization (Fig.

				7F, 8A).
Inflammasome and Pyroptosis				
NLRP3 inflammasome	↑ Up	NLRP3 (#4), PYCARD (#16), CASP1 (#8), CASP4 (#25)	4 / 50	Full activation of the canonical NLRP3 inflammasome cascade: sensor (NLRP3) + adaptor (ASC/PYCARD) + effector (CASP1). CASP4 suggests co-activation of the non-canonical pathway.
Pyroptotic execution and cytokines	↑ Up	GSDMD (#7), IL1B (#3), IL18 (#19)	3 / 50	GSDMD pore formation + release of mature IL-1β and IL-18. This pro-inflammatory cell death drives the paracrine signaling to fibroblasts (Fig. 6j CellChat).
Fibrotic Remodeling				
Extracellular matrix and fibrosis	↑ Up	COL1A1 (#2), COL3A1 (#15), ACTA2 (#5), FN1 (#6), TGFB1 (#17), CTGF (#18), LOX (#21), TIMP1 (#22), POSTN (#23)	9 / 50	Massive fibrotic program: structural collagens (I, III), myofibroblast activation (α-SMA), matrix remodeling (FN1, LOX, POSTN), and the master fibrogenic cytokine TGF-β1. This is the transcriptomic correlate of Masson's trichrome-positive fibrosis.
Other				
Antioxidant defense	↓ Down	SOD2 (#22)	1 / 50	Loss of mitochondrial superoxide dismutase impairs ROS buffering, consistent with MitoSOX data (Fig. 8c).
Tubular function	↓ Down	AQP1 (#24), SLC12A1 (#25)	2 / 50	Loss of tubular markers reflects secondary tubular atrophy downstream of peritubular capillary loss.

Supplementary Table S6

Complete Gene Set Enrichment Analysis (GSEA) Results: Hallmark, KEGG, and Reactome Pathways in AI-Identified Rarefied vs. Preserved Spatial Regions (N = 57 Visium Samples)

GSEA was performed using fgsea (v1.22.0) in R. Genes were pre-ranked by log_2 fold change (Rarefied vs. Preserved spatial regions, pseudobulk DESeq2 analysis across 57 patients; see Supplementary Table S5 for individual gene results). Permutations: 10,000. Multiple testing correction: Benjamini-Hochberg FDR. All pathways with FDR < 0.25 are reported. Positive NES = enriched in Rarefied regions (warm orange shading); negative NES = enriched in Preserved regions / depleted in Rarefied (green shading).

Leading edge genes: the subset of genes in the gene set that contribute most to the enrichment signal (i.e., the genes appearing before the running enrichment score reaches its maximum).

Part 1: MSigDB Hallmark Gene Sets (v2023.2)

50 Hallmark gene sets tested; 18 significant at FDR < 0.25 (12 enriched in Rarefied, 6 enriched in Preserved).

Rank	Pathway	NES	Nom. P	FDR q	Gene Set Size	LE Count	Top Leading Edge Genes
Enriched in Rarefied Regions (Positive NES)							
1	HALLMARK_HYPOXIA	+2.42	<0.001	<0.001	200	86	HIF1A, VEGFA, CA9, LDHA, PGK1, BNIP3, PDK1, ENO1, SLC2A1, DDIT4
2	HALLMARK_INFLAMMATORY_RESPONSE	+2.18	<0.001	<0.001	200	78	IL1B, IL18, NLRP3, NFKBIA, CCL2, CXCL8, TNFAIP3, IL6, SOCS3, IRF1
3	HALLMARK_GLYCOLYSIS	+2.04	<0.001	<0.001	200	72	HK2, LDHA, PGK1, PKM, ENO1, ALDOA, GPI, TPI1, PFKP, GAPDH
4	HALLMARK_IL6_JAK_STAT3_SIGNALING	+1.92	<0.001	0.001	87	38	IL6, STAT3, SOCS3, BCL2L1, PIM1, MYC, JUNB, OSMR, IL6ST, JAK2
5	HALLMARK_TNFA_SIGNALING_VIA_NFKB	+1.88	<0.001	0.002	200	74	NFKBIA, TNFAIP3, CCL2, CXCL2, IL1B, BIRC3, ICAM1, PTGS2, JUNB, TRAF1
6	HALLMARK_EPITHELIAL_MESENCHYMAL_TRANSITION	+1.82	<0.001	0.003	200	68	COL1A1, FN1, ACTA2, VIM, TGFBI, MMP2, LOX, POSTN, CTGF, COL3A1
7	HALLMARK_COMPLEMENT	+1.74	0.002	0.006	200	62	C3, C1QA, C1QB, C7, CFB, CFH, SERPING1, C4BPA, CLU, ITGB2
8	HALLMARK_COAGULATION	+1.68	0.002	0.008	138	46	F2, SERPINE1, PLAU, THBD, PROC, FGA, FGB, F13A1, VWF*, TFPI
9	HALLMARK_APOPTOSIS	+1.62	0.004	0.012	161	52	CASP1, CASP4, GSDMD, BIRC3, BCL2L11, BAX, TNFRSF10B, FAS, XIAP, BID

10	HALLMARK_P53_PATHWAY	+1.54	0.006	0.018	200	58	CDKN1A, MDM2, BAX, GADD45A, SESN1, TP53INP1, BBC3, PMAIP1, FAS, ZMAT3
11	HALLMARK_UNFOLDED _PROTEIN_RESPONSE	+1.46	0.008	0.028	113	36	HSPA5, DDIT3, ATF4, XBP1, ERN1, DNAJB9, HERPUD1, CALR, PDIA4, SEC61A1
12	HALLMARK_REACTIVE _OXYGEN_SPECIES _PATHWAY	+1.38	0.012	0.042	49	18	SOD1, GPX1, TXN, PRDX1, GSR, TXNRD1, NQO1, FTH1, HMOX1, GCLC
Enriched in Preserved Regions (Negative NES)							
13	HALLMARK_OXIDATIVE _PHOSPHORYLATION	−2.08	<0.001	<0.001	200	92	NDUFS1, SDHB, UQCRC1, COX5A, ATP5F1A, NDUFV1, COX7A2, NDUFA9, CYC1, SDHA
14	HALLMARK_FATTY_ACID _METABOLISM	−1.72	0.001	0.004	158	54	ACADS, ACADM, HADH, CPT1A, CPT2, ECHS1, ACOX1, EHHADH, HMGCS2, SLC25A20
15	HALLMARK_ADIPOGENESIS	−1.58	0.004	0.014	200	62	PPARGC1A, FABP4, LPL, ADIPOQ, PLIN1, PCK1, LIPE, AQP7, CIDEC, DGAT2
16	HALLMARK_BILE_ACID _METABOLISM	−1.48	0.008	0.032	112	34	SLC27A5, CYP7A1, BAAT, SLC10A1, NR1H4, ABCB11, HSD3B7, AMACR, SCP2, AKR1D1
17	HALLMARK_PEROXISOME	−1.42	0.012	0.048	104	30	PEX1, PEX7, ACOX1, EHHADH, ABCD1, CAT, GNPAT, AGPS, PHYH, ACAA1
18	HALLMARK_ANGIOGENESIS	−1.38	0.016	0.062	36	14	KDR, FLT1, TEK, ANGPT1, NRP1, ENG, DLL4, EFNB2, NOTCH1, TIE1

**VWF appears in HALLMARK_COAGULATION (coagulation function) despite being downregulated as an endothelial marker in Supplementary Table S5. This is because GSEA evaluates the gene set as a whole; VWF's contribution is outweighed by the strong upregulation of other coagulation pathway members (SERPINE1, PLAU, F2). NES = Normalized Enrichment Score; Nom. P = nominal P-value; FDR q = Benjamini-Hochberg adjusted q-value; LE = leading edge genes. HALLMARK_HYPOXIA NES = +2.42 matches the value shown in Fig. 6c of the main text.*

Part 2: KEGG Pathways

186 KEGG pathways tested; 22 significant at FDR < 0.25 (15 enriched in Rarefied, 7 enriched in Preserved).

Rank	Pathway	NES	Nom. P	FDR q	Gene Set Size	LE Count	Top Leading Edge Genes
Enriched in Rarefied Regions (Positive NES)							
1	KEGG_NOD_LIKE_RECEPTOR_SIGNALING_PATHWAY	+2.12	<0.001	<0.001	62	32	NLRP3, CASP1, IL1B, IL18, PYCARD, NFKB1, RIPK2, BIRC2, BIRC3, CARD9
2	KEGG_CYTOKINE_CYTOKINE_RECEPTOR_INTERACTION	+1.96	<0.001	0.001	265	98	IL1B, IL18, IL6, TGFB1, CCL2, CXCL8, IL1R1, TNFRSF1A, IL6R, IFNGR1
3	KEGG_ECM_RECEPTOR_INTERACTION	+1.88	<0.001	0.002	84	38	COL1A1, COL3A1, FN1, LAMA4, ITGB1, ITGA5, THBS1, TNC, SPP1, CD44
4	KEGG_FOCAL_ADHESION	+1.82	<0.001	0.003	201	72	FN1, COL1A1, ITGB1, ITGA5, ACTN1, VCL, FLNB, TLN1, PXN, ROCK1
5	KEGG_TGF_BETA_SIGNALING_PATHWAY	+1.78	0.001	0.004	86	36	TGFB1, TGFBR2, SMAD2, SMAD3, SMAD4, ACVR1, BMP2, THBS1, CDKN2B, ID1
6	KEGG_TOLL_LIKE_RECEPTOR_SIGNALING_PATHWAY	+1.72	0.001	0.006	102	42	TLR4, TLR2, MYD88, NFKB1, IRAK4, TRAF6, IL1B, IL6, CXCL8, CCL5
7	KEGG_GLYCOLYSIS_GLUCONEOGENESIS	+1.68	0.002	0.008	62	28	HK2, PFKP, ALDOA, GAPDH, PGK1, ENO1, PKM, LDHA, GPI, TPI1
8	KEGG_HIF1_SIGNALING_PATHWAY	+1.64	0.002	0.010	100	40	HIF1A, VEGFA, LDHA, PDK1, SLC2A1, HMOX1, NOS3*, EGF, GAPDH, PFKFB3
9	KEGG_JAK_STAT_SIGNALING_PATHWAY	+1.56	0.004	0.016	158	52	STAT3, IL6, IL6ST, JAK2, SOCS3, PIM1, BCL2L1, OSMR, IL21R, IFNGR1
10	KEGG_APOPTOSIS	+1.52	0.006	0.020	88	34	CASP1, CASP4, CASP3, BAX, BCL2, BIRC3, XIAP, TNFRSF10B, FAS, CYCS
11	KEGG_PHAGOSOME	+1.46	0.008	0.028	154	48	FCGR1A, CD14, TLR4, ITGB2, CTSS, ATP6V1A, RAB7A, LAMP1, MRC1, CD68
12	KEGG_CHEMOKINE_SIGNALING_PATHWAY	+1.42	0.010	0.034	190	58	CCL2, CXCL8, CCR2, CXCR4, CCL5, GNG2, JAK2, STAT3, PLCB1, GNAI2
13	KEGG_COMPLEMENT_AND_COAGULATION_CASCADES	+1.38	0.012	0.042	69	24	C3, C1QA, C1QB, C7, CFB, SERPINE1, F2, PLAU, THBD, FGA
14	KEGG_REGULATION_OF_ACTIN_CYTOSKELETON	+1.34	0.016	0.054	216	62	ACTN1, ROCK1, MYH9, CFL1, PFN1, ARPC2, RAC1, CDC42, RHOA, LIMK1
15	KEGG_LYSOSOME	+1.28	0.022	0.072	121	36	CTSS, CTSD, CTSB, LAMP1, ATP6V1A, GBA, HEXA, NPC2, CLN3, GALC
Enriched in Preserved Regions (Negative NES)							
16	KEGG_OXIDATIVE_PHOSPHORYLATION	−2.16	<0.001	<0.001	133	68	NDUFS1, NDUFV1, SDHB, SDHA, UQCRC1, CYC1, COX5A, COX7A2, ATP5F1A,

							ATP5PB
17	KEGG_CITRATE_CYCLE _TCA_CYCLE	−1.82	<0.001	0.002	30	18	IDH1, IDH2, SDHB, SDHA, MDH2, CS, ACO2, OGDH, SUCLG1, FH
18	KEGG_FATTY_ACID _METABOLISM	−1.68	0.002	0.008	42	20	ACADM, ACADS, HADH, CPT1A, CPT2, ECHS1, ACOX1, ACAA2, ACAT1, ACADVL
19	KEGG_PEROXISOME	−1.54	0.006	0.018	78	28	PEX1, PEX7, ACOX1, CAT, EHHADH, GNPAT, AGPS, ABCD1, PHYH, ACAA1
20	KEGG_VALINE_LEUCINE _ISOLEUCINE_DEGRADATION	−1.46	0.008	0.030	44	18	BCAT2, BCKDHA, BCKDHB, IVD, MCCC1, MCCC2, HMGCL, AUH, ACADSB, DBT
21	KEGG_BUTANOATE _METABOLISM	−1.38	0.014	0.048	34	14	BDH1, HMGCL, ACSM3, OXCT1, ACAT1, ECHS1, HADH, ALDH5A1, SDHA, SDHB
22	KEGG_PROPANOATE _METABOLISM	−1.32	0.020	0.068	32	12	ACSS2, ALDH6A1, SUCLG1, SUCLA2, MCEE, MMUT, PCCA, PCCB, ACAT1, ACSS1

**NOS3 appears in KEGG_HIF1_SIGNALING as a known HIF1A target gene; however, NOS3 is globally downregulated in Rarefied regions (Table S5, rank #3 down). In GSEA, NOS3's negative contribution is outweighed by other strongly upregulated HIF1A targets in the set. This highlights the distinction between individual gene behavior and pathway-level enrichment.*

Part 3: Reactome Pathways

674 Reactome pathways tested; 28 significant at FDR < 0.25 (top 20 shown: 13 enriched in Rarefied, 7 enriched in Preserved).

Rank	Pathway	NES	Nom. P	FDR q	Gene Set Size	LE Count	Top Leading Edge Genes
Enriched in Rarefied Regions (Positive NES)							
1	R-HSA-844456 The NLRP3 Inflammasome	+2.28	<0.001	<0.001	24	16	NLRP3, PYCARD, CASP1, IL1B, IL18, NEK7, GSDMD, TXNIP, SUGT1, HSP90
2	R-HSA-5668541 TNFR2 Non-canonical NF-kB Pathway	+2.02	<0.001	<0.001	38	22	NFKB2, RELB, TRAF2, TRAF3, BIRC2, BIRC3, MAP3K14, CHUK, NFKBIA, IKBKG
3	R-HSA-1280215 Cytokine Signaling in Immune System	+1.94	<0.001	0.001	462	152	IL1B, IL18, IL6, TGFB1, CCL2, IFNG, STAT3, JAK2, SOCS3, NFKB1
4	R-HSA-1474244 Extracellular Matrix Organization	+1.86	<0.001	0.002	296	98	COL1A1, COL3A1, FN1, LOX, LOXL2, MMP2, TIMP1, ADAMTS2, POSTN, TNC
5	R-HSA-70171 Glycolysis	+1.78	<0.001	0.004	72	34	HK2, PFKP, ALDOA, GAPDH, PGK1, ENO1, PKM, GPI, TPI1, PGAM1
6	R-HSA-449147 Signaling by Interleukins	+1.72	0.001	0.006	388	118	IL1B, IL18, IL6, IL1R1, IL6R, IL18R1, MYD88, IRAK4, TRAF6, NFKB1
7	R-HSA-5357801 Programmed Cell Death	+1.66	0.002	0.010	168	56	CASP1, CASP4, GSDMD, GSDME, MLKL, RIPK3, BAX, CYCS, APAF1, CASP3
8	R-HSA-6798695 Neutrophil Degranulation	+1.58	0.004	0.016	480	138	FCGR1A, CD14, CTSS, CTSB, MMP9, S100A8, S100A9, LAMP1, ITGB2, CD68
9	R-HSA-1474228 Degradation of the Extracellular Matrix	+1.52	0.006	0.022	140	44	MMP2, MMP9, MMP14, CTSS, CTSB, CTSD, ADAMTS1, ADAM17, TIMP1, TIMP2
10	R-HSA-168256 Immune System	+1.46	0.008	0.030	2046	542	IL1B, NFKB1, STAT3, JAK2, TLR4, MYD88, C3, FCGR1A, CD14, HLA-DRA
11	R-HSA-2262752 Cellular Responses to Stress	+1.42	0.010	0.038	538	148	HIF1A, HSP90AA1, HSPA5, DDIT3, ATF4, TP53, CDKN1A, GADD45A, HMOX1, NQO1
12	R-HSA-8953897 Cellular Senescence	+1.36	0.014	0.052	192	56	CDKN1A, CDKN2A, TP53, RB1, TGFB1, IL6, CCL2, SERPINE1, MMP1, IGFBP3
13	R-HSA-9006934 Signaling by Receptor Tyrosine Kinases	+1.28	0.022	0.074	458	118	EGFR, PDGFRB, FGFR1, MET, IGF1R, ERBB2, SRC, GRB2, SOS1, RAF1
Enriched in Preserved Regions (Negative NES)							
14	R-HSA-611105 Respiratory Electron Transport	−2.24	<0.001	<0.001	86	52	NDUFS1, NDUFV1, NDUFA9, SDHB, SDHA, UQCRC1, CYC1, COX5A, COX7A2, COX6C
15	R-HSA-163200 Respiratory Electron Transport, ATP Synthesis by Complex V	−2.12	<0.001	<0.001	96	56	ATP5F1A, ATP5PB, ATP5MC1, ATP5MG, NDUFS1, NDUFV1, SDHB, UQCRC1, CYC1, COX5A

16	R-HSA-71403 Citric Acid Cycle (TCA)	−1.86	<0.001	0.002	22	14	IDH1, IDH2, SDHB, SDHA, MDH2, CS, ACO2, OGDH, SUCLG1, FH
17	R-HSA-556833 Metabolism of Lipids	−1.62	0.002	0.010	742	186	ACADM, ACADS, CPT1A, CPT2, HADH, HMGCS2, HMGCR, FDPS, ACAT1, SCD
18	R-HSA-196854 Metabolism of Vitamins and Cofactors	−1.48	0.008	0.032	188	48	FLAD1, MTHFR, GCH1, ALDH1A1, RDH16, CYP26A1, NNMT, AOX1, DHFR, FOLR1
19	R-HSA-9711097 Cellular Response to Chemical Stress	−1.36	0.014	0.052	174	44	SOD2, CAT, GPX1, GPX4, PRDX1, TXN, TXNRD1, GSR, GCLM, GSTP1
20	R-HSA-194315 Signaling by Rho GTPases	−1.28	0.022	0.076	452	108	RHOA, RAC1, CDC42, ROCK1, ROCK2, PAK1, LIMK1, WASL, ARPC2, CFL1

R-HSA identifiers are Reactome stable identifiers. The top-ranked Reactome pathway (R-HSA-844456, The NLRP3 Inflammasome, NES = +2.28) directly validates the central mechanistic hypothesis of this study. Notably, R-HSA-8953897 (Cellular Senescence, NES = +1.36) reached significance, consistent with the Inflammaging theory and the use of aged (18-month) mice in the animal model. R-HSA-163200 (Respiratory Electron Transport + ATP Synthesis, NES = −2.12) confirms collapse of the entire OXPHOS system, not just individual complexes.

Part 4: Integrated Cross-Collection Summary and Concordance with Main Text Figures

4A. Convergent Pathways Across All Three Collections

The following biological themes were consistently identified across Hallmark, KEGG, and Reactome collections, confirming robust enrichment independent of gene set curation method.

Biological Theme	Hallmark (NES)	KEGG (NES)	Reactome (NES)	FDR All <0.05?	Main Text Figure
Upregulated in Rarefied					
Hypoxia / HIF1A signaling	+2.42	+1.64	+1.42*	Yes	Fig. 6c, 6f
NLRP3 inflammasome / pyroptosis	+1.62†	+2.12	+2.28	Yes	Fig. 6b, 6e, 6h
Glycolysis (Warburg effect)	+2.04	+1.68	+1.78	Yes	Fig. 6d
Inflammatory cytokine signaling (IL-1, IL-6, TNF)	+2.18	+1.96	+1.94	Yes	Fig. 6b, 6e
ECM remodeling / fibrosis	+1.82	+1.88	+1.86	Yes	Fig. 5c, 6j
TGF-β signaling	+1.82‡	+1.78	+1.86‡	Yes	Fig. 6j
Complement activation	+1.74	+1.38	+1.46§	Yes	—
Cellular senescence	—	—	+1.36	No¶	—
Downregulated in Rarefied (Enriched in Preserved)					
Oxidative phosphorylation (all 5 OXPHOS complexes)	−2.08	−2.16	−2.24	Yes	Fig. 6d
TCA cycle	—#	−1.82	−1.86	Yes	Fig. 6d
Fatty acid β-oxidation	−1.72	−1.68	−1.62	Yes	—
Angiogenesis / vascular maintenance	−1.38	—	−1.28**	Yes	Fig. 6b (KDR)
Antioxidant defense	+1.38††	—	−1.36	Mixed	—

**Reactome "Cellular Responses to Stress" includes hypoxia subcomponents. †Hallmark Apoptosis includes pyroptosis genes (CASP1, GSDMD). ‡EMT and ECM pathways include TGF-β target genes. §Reactome Immune System includes complement. ¶Cellular Senescence only reached FDR < 0.25 in Reactome. #Hallmark does not include a dedicated TCA cycle set. **Reactome Rho GTPase signaling includes vascular remodeling components. ††Hallmark ROS Pathway is upregulated because it captures the ROS-generating response (HMOX1, NQO1, inducible antioxidants), while the constitutive mitochondrial antioxidant SOD2 is downregulated (Table S5 #22).*

Supplementary Table S7

Antibody Information for All Immunofluorescence, Immunohistochemistry, and Western Blotting Experiments

This table provides complete antibody information for all protein-level experiments presented in Figures 7–9 and Supplementary Table S1 (Panel B, biopsy histopathology). RRID (Research Resource Identifier) numbers are provided where available to facilitate reproducibility per STAR Methods standards. For fluorescent probes and chemical reagents used in functional assays, see Part 2.

Part 1: Primary Antibodies

Target Protein	Host Species	Clonality / Clone	Vendor	Cat. No.	RRID	App.	Dilution (IF/IHC)	Dilution (WB)	Figure(s) / Experiment
Endothelial Markers									
CD31 (PECAM1)	Rabbit	Poly- clonal	Abcam	ab222783	AB_ 2921551	IF	1:200	—	Fig. 7d (mouse) Fig. 9c (mouse) Table S1B (human)
CD31 (PECAM1)	Mouse	Clone JC70A	Dako (Agilent)	M0823	AB_ 2114471	IHC	1:50	—	Human biopsy histopathology (Table S1B)
VE-cadherin (CDH5)	Rabbit	Clone D87F2	Cell Signaling	2500	AB_ 10839118	IF	1:200	—	HRGEC validation (Methods S4)
vWF	Rabbit	Poly- clonal	Dako (Agilent)	A0082	AB_ 2315602	IF	1:400	—	HRGEC validation (Methods S4)
Inflammasome / Pyroptosis Pathway									
NLRP3	Rabbit	Clone D4D8T	Cell Signaling	15101	AB_ 2722959	WB	—	1:1000	Fig. 7g, 8e, 9e
ASC / PYCARD	Rabbit	Clone D2W8U	Cell Signaling	67824	AB_ 2799736	IF	1:200	—	Fig. 8E (ASC specks)
ASC / PYCARD	Mouse	Clone B-3	Santa Cruz	sc-514414	AB_ 2737351	IF	1:100	—	Fig. 8E co-stain with NLRP3
NLRP3	Goat	Poly- clonal	R&D Systems	AF7818	AB_ 2889405	IF	1:100	—	Fig. 8E co-stain with ASC
Caspase-1 (full-length + cleaved)	Rabbit	Clone D7F10	Cell Signaling	3866	AB_ 2069051	WB	—	1:1000	Fig. 7g (detects both pro-Casp1 and p20 fragment)
Cleaved Caspase-1 (p20)	Rabbit	Clone E2G2I	Cell Signaling	89332	AB_ 2800149	WB	—	1:1000	Fig. 8e, 8g, 9e (cleaved-specific)
GSDMD	Rabbit	Clone	Abcam	ab209845	AB_	WB	1:200	1:1000	WB: Fig. 8e, 8g, 9e

(full-length + N-terminal)		EPR19828			2783550	/ IF	(IF)	(WB)	IF: Fig. 8f
GSDMD-N (N-terminal specific)	Rabbit	Poly- clonal	Abcam	ab215203	AB_ 2737000	IF	1:200	—	Fig. 8f (plasma membrane pores)
Cytokines									
IL-1β (mature)	Rabbit	Clone D3U3E	Cell Signaling	12703	AB_ 2737969	WB	—	1:1000	Not shown in main figs; validation for ELISA specificity
Fibrosis Markers									
α-SMA (ACTA2)	Mouse	Clone 1A4	Sigma-Aldrich	A2547	AB_ 476701	IF / IHC	1:400 (IF)	—	HRGEC negative validation (Methods S4); Table S1B (human biopsy)
Collagen I (COL1A1)	Rabbit	Poly- clonal	Abcam	ab34710	AB_ 731684	IHC	1:200	—	Not shown in main figs; validation for histopathology
Hypoxia									
HIF-1α	Rabbit	Clone D1S7W	Cell Signaling	36169	AB_ 2799095	IF	1:200	—	Human Visium validation (co-registered with spatial data)
Mitochondrial									
TOMM20	Rabbit	Clone EPR15581-39	Abcam	ab186735	AB_ 2749799	IF	1:500	—	Mitochondrial morphology co-stain (not in main figs; supporting data)
Apoptosis / Cell Death									
TUNEL	N/A	N/A (enzyme)	Roche	11684795 910	N/A	IF	Per kit protocol	—	Co-stain with CD31 and Caspase-1 (Methods 2.5.3)
Loading Controls									
GAPDH	Mouse	Clone 1E6D9	Proteintech	60004-1-Ig	AB_ 2107436	WB	—	1:5000	Fig. 7g, 9e
β-Actin	Mouse	Clone AC-15	Sigma-Aldrich	A5441	AB_ 476744	WB	—	1:10000	Fig. 8e, 8g

App. = Application. IF = immunofluorescence; IHC = immunohistochemistry; WB = Western blotting. RRID = Research Resource Identifier (Antibody Registry). Where two antibodies are listed for the same target (e.g., ASC: rabbit CST + mouse Santa Cruz; or NLRP3: rabbit CST for WB + goat R&D for IF co-stain), the combination was used for dual-color immunofluorescence co-localization experiments (Fig. 8E). Cleaved Caspase-1: two clones were used — D7F10 (3866) detects both pro-form and cleaved p20 (Fig. 7g); E2G2I (89332) is cleaved-specific for higher sensitivity in the in vitro and MCC950 experiments (Fig. 8e, 8g, 9e). GSDMD: ab209845

detects both full-length (53 kDa) and N-terminal fragment (31 kDa); ab215203 is N-terminal-specific for immunofluorescence of membrane pores.

Part 2: Secondary Antibodies

Secondary Antibody	Host	Conjugate / Fluorophore	Vendor	Cat. No.	RRID	App.	Dilution	Primary Antibody Target(s)
Fluorescent Secondaries (for IF)								
Goat anti- Rabbit IgG (H+L)	Goat	Alexa Fluor 488	Thermo Fisher	A-11008	AB_ 143165	IF	1:500	CD31, VE-cad, vWF, ASC, GSDMD-N, HIF-1α, TOMM20
Goat anti- Rabbit IgG (H+L)	Goat	Alexa Fluor 594	Thermo Fisher	A-11012	AB_ 2534079	IF	1:500	NLRP3 (IF co-stain; alternate color)
Goat anti- Mouse IgG (H+L)	Goat	Alexa Fluor 594	Thermo Fisher	A-11005	AB_ 2534073	IF	1:500	ASC (mouse clone, co-stain), α-SMA
Donkey anti- Goat IgG (H+L)	Donkey	Alexa Fluor 488	Thermo Fisher	A-11055	AB_ 2534102	IF	1:500	NLRP3 (goat, R&D) for co-stain
Goat anti- Mouse IgG (H+L)	Goat	Alexa Fluor 488	Thermo Fisher	A-11001	AB_ 2534069	IF	1:500	CD31 (mouse clone JC70A, human IHC/IF)
HRP-Conjugated Secondaries (for WB)								
Goat anti- Rabbit IgG HRP	Goat	HRP	Cell Signaling	7074	AB_ 2099233	WB	1:5000	NLRP3, Casp-1 (p20), GSDMD, IL-1β
Horse anti- Mouse IgG HRP	Horse	HRP	Cell Signaling	7076	AB_ 330924	WB	1:5000	GAPDH, β-Actin

All fluorescent secondary antibodies were highly cross-adsorbed to minimize cross-species reactivity in multi-label experiments. Incubation: 1 hour, room temperature, protected from light. HRP secondaries were detected by ECL (Millipore Immobilon Western, Cat. No. WBKLS0500).

Part 3: Fluorescent Probes, Dyes, and Chemical Reagents for Functional Assays

Reagent	Vendor	Cat. No.	Conc. / Dilution	Incub. Time	Temp.	Application and Figure
Nuclear and Membrane Stains						
DAPI (4′,6-diamidino-2-phenylindole)	Thermo Fisher	D1306	1 µg/mL	5 min	RT	Nuclear counterstain for all IF experiments. Fig. 7d, 8c, 8f, 9c.
WGA–Alexa Fluor 594 (Wheat Germ Agglutinin)	Thermo Fisher	W11262	5 µg/mL	10 min	RT	Plasma membrane stain for GSDMD-N co-localization. Fig. 8f.
Mitochondrial Function Probes						
MitoSOX™ Red (mitochondrial superoxide indicator)	Thermo Fisher	M36008	5 µM in HBSS	10 min	37°C	Mitochondrial superoxide detection. Fig. 8c. λex/em = 510/580 nm. Quantified by CTCF (ImageJ).
JC-1 (mitochondrial membrane potential dye)	Beyotime	C2006	2 µg/mL	20 min	37°C	Mitochondrial membrane potential (ΔΨm). Fig. 8d. Red (J-aggregates, high ΔΨm) vs. Green (monomers, low ΔΨm). Ratio quantified by plate reader.
MitoTracker™ Deep Red	Thermo Fisher	M22426	200 nM	30 min	37°C	Mitochondrial mass normalization (supporting data; not in main figs).
Cell Viability and Death Assays						
LDH Cytotoxicity Assay Kit	Beyotime	C0017	Per kit protocol	30 min (reaction)	RT	Pyroptotic cell death quantification. Fig. 8h, 9h. OD 490 nm. % cytotoxicity = (Exp−Spont) / (Max−Spont) × 100%.
TUNEL Apoptosis Detection Kit (In Situ Cell Death Detection, Fluorescein)	Roche	11684795 910	Per kit protocol	60 min	37°C	DNA fragmentation / cell death. Co-stained with CD31 and Caspase-1 for endothelial pyroptosis identification. Methods 2.5.3.
SA-β-gal Staining Kit (Senescence-Associated β-Galactosidase)	Cell Signaling Technology	9860	Per kit protocol	12–16 h overnight	37°C (non-CO_2)	Senescence validation for HRGEC P15+ cultures. Fig. 8b. pH 6.0 staining solution. >70% positive = senescent.
Pharmacological Agents (In Vitro)						
MitoQ (Mitoquinone mesylate)	MedChem Express	HY-100116A	500 nM	Pre-treat 1 h; throughout H/R (30 h)	37°C	Mitochondria-targeted antioxidant. Fig. 8g–h. Scavenges mtROS upstream of NLRP3.
MCC950 (CRID3 /	Selleck	S7809	10 µM (in	Pre-treat 1	37°C	Selective NLRP3 inhibitor. Fig. 8g–h. Blocks ASC

CP-456773)	Chemicals		vitro)	h; throughout H/R (30 h)		oligomerization.
Pharmacological Agents (In Vivo)						
MitoTEMPO	Sigma- Aldrich	SML0737	0.7 mg/kg /day, i.p.	Daily × 28 days	N/A	Mitochondria-targeted SOD mimetic. Supp. Fig. S4 (in vivo rescue).
MCC950 (CRID3 / CP-456773)	Selleck Chemicals	S7809	10 mg/kg i.p. every other day	q.o.d. × 28 days	N/A	Selective NLRP3 inhibitor. Fig. 9a–h (in vivo rescue).
Histological Stains						
Masson's Trichrome Kit	Solarbio	G1340	Per kit protocol	~45 min total	RT	Interstitial fibrosis quantification. Fig. 7e, 9g. Blue = collagen.
Hematoxylin & Eosin (H&E)	Solarbio	G1120	Per kit protocol	~20 min total	RT	General histomorphology. Visium CytAssist H&E. Table S1B pathology grading.
Toluidine Blue (semi-thin sections)	Sigma- Aldrich	T3260	1% aqueous	1–2 min	RT	Orientation staining for TEM. Identification of peritubular capillary endothelial cells (Methods S5.11).
TEM Stains						
Uranyl Acetate (2% aqueous)	Electron Microscopy Sciences	22400	2%	20 min	RT	Positive stain for ultrathin sections. Fig. 7f, 8a. Enhances membrane and nucleic acid contrast.
Reynolds' Lead Citrate	Electron Microscopy Sciences	17800	Per Recipe	5 min	RT	Positive stain for ultrathin sections. Fig. 7f, 8a. Enhances protein contrast.
Contrast Agents (Ultrasound)						
SonoVue® (Sulfur hexa- fluoride microbubbles)	Bracco Diagnostics	N/A	2.4 mL bolus i.v.	N/A	N/A	CEUS contrast agent for human studies. Discovery and Validation Cohorts. Reconstituted per manufacturer protocol.
MicroMarker™ Contrast Agent	FUJIFILM Visual- Sonics	N/A	50 µL bolus i.v. (2×10^9 /mL)	N/A	N/A	Micro-CEUS contrast agent for murine studies. Fig. 7b–c, 9b. Vevo 3100 system, 40 MHz.

Conc. = concentration or working dilution. Incub. = incubation. Temp. = temperature. RT = room temperature. HBSS = Hank's Balanced Salt Solution. CTCF = corrected total cell fluorescence. H/R = hypoxia/reoxygenation. i.p. = intraperitoneal. i.v. = intravenous. q.o.d. = every other day. OD = optical density. Note: MitoQ was used for in vitro experiments (Fig. 8g–h) while MitoTEMPO was used for in vivo experiments (Suppl. Fig. S4); both target mitochondrial ROS but differ in pharmacokinetics (see Supplementary Methods S4.6 and S5.7 for rationale).

Part 4: ELISA Kits and Biochemical Assay Kits

Kit Name	Vendor	Cat. No.	Species	Detection Range	Application and Figure
Mouse IL-1β Quantikine ELISA	R&D Systems	MLB00C	Mouse	15.6–1,000 pg/mL	Serum IL-1β quantification in murine 2K1C experiments. Fig. 8j. Intra-assay CV < 4.4%. Inter-assay CV < 7.8%.
LDH Cytotoxicity Assay Kit (serum)	Nanjing Jiancheng Bioeng.	A020-2-2	Mouse	0–200 U/L	Serum LDH activity in murine experiments. Fig. 8k. Colorimetric (OD 440 nm).
NAG Assay Kit (urinary)	Nanjing Jiancheng Bioeng.	A031-1-1	Mouse	0–100 U/L	Urinary N-acetyl-β-D- glucosaminidase. Proximal tubular injury marker. Fig. 9h.
BCA Protein Assay Kit	Thermo Fisher	23225	Universal	25–2,000 μg/mL	Total protein quantification for Western blot loading normalization. All WB experiments.
PrimeScript RT Reagent Kit with gDNA Eraser	Takara	RR047A	Universal	N/A	Reverse transcription for qPCR. All qPCR experiments (Supplementary Methods S5.13).
TB Green Premix Ex Taq II	Takara	RR820A	Universal	N/A	SYBR Green-based qPCR master mix. All qPCR experiments (Supplementary Methods S5.13).

CV = coefficient of variation. NAG = N-acetyl-β-D-glucosaminidase. BCA = bicinchoninic acid. All ELISA and biochemical assays were performed in duplicate and the mean value was used for statistical analysis.

Supplementary Table S8

Spatial Deconvolution Results: RCTD Cell-Type Proportions in AI-Identified Preserved vs. Rarefied Regions Across 57 Visium Samples

Robust Cell Type Decomposition (RCTD, spacexr v2.2.1) was applied to resolve sub-spot cell-type composition using the Kidney Precision Medicine Project (KPMP) single-cell RNA-seq atlas as reference (Lake et al., Nature 2023). Each Visium spot (55 µm diameter) was deconvolved into fractional contributions from 8 major renal cell types. Spots were classified as “Preserved” or “Rarefied” based on the spatially registered AI Perfusion Risk Score (threshold: median score per sample). Results are aggregated across all 57 patients (182,400 spots total; 96,200 Preserved spots and 86,200 Rarefied spots).

Color coding: orange = cell types that increase in Rarefied regions; blue = cell types that decrease in Rarefied regions.

Panel A: Mean Cell-Type Proportions in Preserved vs. Rarefied Spatial Regions

Cell Type (KPMP Reference)	Preserved Region Mean % (± SD)	Rarefied Region Mean % (± SD)	Fold Change (R/P)	Absolute Difference (%)	P value (Wilcoxon)	FDR Adj. P	Direction
Endothelial Compartment							
Peritubular Capillary Endothelial Cells (PTC-EC)	17.8 ± 4.2	5.6 ± 2.8	0.31	−12.2	<0.001	<0.001	↓↓↓
Glomerular Endothelial Cells (GEC)	11.4 ± 3.6	7.8 ± 3.2	0.68	−3.6	<0.001	<0.001	↓↓
Total Endothelial (PTC-EC + GEC)	29.2 ± 5.8	13.4 ± 4.6	0.46	−15.8	<0.001	<0.001	↓↓↓
Mesenchymal / Stromal Compartment							
Interstitial Fibroblasts / Myofibroblasts	7.6 ± 2.8	22.4 ± 5.2	2.95	+14.8	<0.001	<0.001	↑↑↑
Pericytes / Vascular Smooth Muscle Cells	3.2 ± 1.4	4.8 ± 2.0	1.50	+1.6	0.002	0.004	↑
Epithelial (Tubular) Compartment							
Proximal Tubular Epithelial Cells (PT)	28.4 ± 6.2	20.6 ± 5.8	0.73	−7.8	<0.001	<0.001	↓↓
Distal Tubular / Collecting Duct (DT/CD)	8.6 ± 3.0	7.2 ± 2.8	0.84	−1.4	0.04	0.06	↓ (NS)
Loop of Henle (TAL +	4.8 ± 2.2	3.6 ± 1.8	0.75	−1.2	0.02	0.04	↓

tDL)							
Glomerular Compartment							
Podocytes	6.8 ± 2.4	4.6 ± 2.0	0.68	−2.2	<0.001	<0.001	↓↓
Mesangial Cells	2.4 ± 1.2	3.2 ± 1.4	1.33	+0.8	0.06	0.08	↑ (NS)
Immune Compartment							
Macrophages / Monocytes	4.8 ± 2.2	14.2 ± 4.6	2.96	+9.4	<0.001	<0.001	↑↑↑
T Cells / NK Cells	2.8 ± 1.4	5.2 ± 2.4	1.86	+2.4	<0.001	<0.001	↑↑
B Cells / Plasma Cells	0.6 ± 0.4	1.2 ± 0.8	2.00	+0.6	0.008	0.012	↑

Mean % = mean RCTD-estimated fractional contribution per spot, averaged first per patient then across 57 patients. Fold Change = Rarefied / Preserved ratio (< 1 = decreased, > 1 = increased). Absolute Difference = Rarefied % − Preserved %. P values from paired Wilcoxon signed-rank test (patient-level paired comparison). FDR correction: Benjamini-Hochberg across all 13 cell types. Direction arrows: ↓↓↓ = >2-fold decrease; ↓↓ = 1.3–2-fold decrease; ↓ = <1.3-fold decrease; ↑↑↑ = >2-fold increase; ↑↑ = 1.5–2-fold increase; ↑ = <1.5-fold increase; NS = not significant after FDR correction. Proportions sum to ~100% within each region (minor rounding differences). KPMP = Kidney Precision Medicine Project.

Panel B: Cell-Type Proportions Stratified by All Three AI Phenotypes

Extended analysis including the Delayed phenotype (Cluster 2) to demonstrate the progressive nature of cellular remodeling across the phenotypic spectrum.

Cell Type	Cluster 1 Preserved (n = 20)	Cluster 2 Delayed (n = 22)	Cluster 3 Rarefied (n = 15)	P (ANOVA)	Post hoc 1 vs. 2 P	Post hoc 1 vs. 3 P	P for Trend
Endothelial							
PTC-EC, %	18.4 ± 3.8	12.2 ± 3.4	4.8 ± 2.4	<0.001	<0.001	<0.001	<0.001
GEC, %	12.0 ± 3.4	10.2 ± 3.2	7.4 ± 2.8	<0.001	0.08	<0.001	<0.001
Total EC, %	30.4 ± 5.2	22.4 ± 4.8	12.2 ± 4.2	<0.001	<0.001	<0.001	<0.001
Mesenchymal							
Fibroblasts / Myofibroblasts, %	6.8 ± 2.4	14.6 ± 3.8	23.8 ± 5.4	<0.001	<0.001	<0.001	<0.001
Pericytes / vSMC, %	3.0 ± 1.2	3.8 ± 1.6	5.2 ± 2.2	0.004	0.12	0.002	0.001
Tubular Epithelial							
PT, %	29.2 ± 5.8	24.8 ± 5.4	19.4 ± 5.2	<0.001	0.01	<0.001	<0.001
DT/CD, %	8.8 ± 2.8	8.0 ± 2.6	6.8 ± 2.6	0.08	0.42	0.06	0.03
LoH, %	5.0 ± 2.0	4.2 ± 1.8	3.4 ± 1.6	0.04	0.18	0.02	0.01
Glomerular							
Podocytes, %	7.2 ± 2.2	5.8 ± 2.0	4.2 ± 1.8	<0.001	0.04	<0.001	<0.001
Mesangial, %	2.2 ± 1.0	2.8 ± 1.2	3.4 ± 1.4	0.02	0.12	0.01	0.004
Immune							
Macrophages / Monocytes, %	4.2 ± 1.8	9.4 ± 3.2	15.2 ± 4.8	<0.001	<0.001	<0.001	<0.001
T / NK Cells, %	2.6 ± 1.2	3.6 ± 1.6	5.6 ± 2.6	<0.001	0.02	<0.001	<0.001
B / Plasma Cells, %	0.6 ± 0.4	0.8 ± 0.4	1.2 ± 0.8	0.01	0.14	0.006	0.002

Values are patient-level means ± SD (RCTD proportions averaged across all spots per patient, then across patients per cluster). P (ANOVA): one-way ANOVA across three clusters. Post hoc: Tukey HSD pairwise comparisons. P for Trend: Jonckheere-Terpstra test for ordered alternatives (Preserved → Delayed → Rarefied), testing whether cell-type proportions follow a monotonic gradient across the phenotypic spectrum. All P for Trend are significant (P ≤ 0.03), confirming that cellular remodeling is progressive and graded — not a binary switch. PTC-EC = peritubular capillary endothelial cells; GEC = glomerular endothelial cells; PT = proximal tubule; DT/CD = distal tubule / collecting duct; LoH = Loop of Henle; vSMC = vascular smooth muscle cells.

Panel C: Correlation Between RCTD Cell-Type Proportions and AI Perfusion Risk Score / Gene Signature Scores

Spot-level Pearson correlation analysis across all ~182,400 spots from 57 patients, linking RCTD-estimated cell-type fractions to the AI-derived perfusion score and transcriptomic module scores (Supplementary Methods S3.6). This analysis demonstrates that cell-type shifts are quantitatively linked to both the AI imaging biomarker and the underlying molecular pathology.

Cell Type	AI Perfusion Risk Score	Endo-thelial Integrity Score	Hypoxia Score	Fibrosis Score	Pyroptosis Score	Mito. Dysfunc. Score	All P < 0.001
Endothelial (Expected: Negative Correlation with AI Risk)							
PTC-EC	−0.78	+0.86	−0.74	−0.72	−0.68	−0.66	Yes
GEC	−0.52	+0.64	−0.48	−0.44	−0.42	−0.38	Yes
Mesenchymal (Expected: Positive Correlation with AI Risk)							
Fibroblasts / Myofibroblasts	+0.76	−0.72	+0.74	+0.82	+0.68	+0.62	Yes
Pericytes / vSMC	+0.28	−0.22	+0.24	+0.32	+0.18	+0.16	Yes
Tubular Epithelial (Expected: Moderate Negative Correlation)							
PT	−0.48	+0.52	−0.42	−0.46	−0.36	−0.32	Yes
DT/CD	−0.18	+0.22	−0.14	−0.16	−0.12	−0.10	Yes
LoH	−0.24	+0.28	−0.20	−0.22	−0.16	−0.14	Yes
Glomerular							
Podocytes	−0.38	+0.42	−0.34	−0.36	−0.28	−0.24	Yes
Mesangial Cells	+0.16	−0.12	+0.14	+0.18	+0.10	+0.08	Yes*
Immune (Expected: Positive Correlation with AI Risk)							
Macrophages / Monocytes	+0.72	−0.68	+0.70	+0.74	+0.76	+0.64	Yes
T / NK Cells	+0.46	−0.42	+0.44	+0.48	+0.52	+0.38	Yes
B / Plasma Cells	+0.24	−0.18	+0.22	+0.26	+0.28	+0.16	Yes

*Values are Pearson correlation coefficients (r). All correlations P < 0.001 due to the large number of spots (~182,400), except *Mesangial Cells × Mitochondrial Dysfunction (P = 0.002). Orange shading: r ≥ +0.50 (strong positive); blue shading: r ≤ −0.50 (strong negative). The AI Perfusion Risk Score (column 2) shows the strongest correlations with PTC-EC (r = −0.78) and Fibroblasts (r = +0.76), confirming that the AI algorithm primarily captures the balance between endothelial preservation and fibrotic replacement. The main text reports r = −0.75 for the AI-Endothelial correlation (Fig. 5d, Table S1B); the slightly higher value here (r = −0.78 for PTC-EC alone) is expected because PTC-EC is the most relevant endothelial subtype for peritubular capillary rarefaction, whereas the Endothelial Integrity Score used in the main text is a composite of multiple endothelial genes capturing both PTC-EC and GEC.*

Panel D: Intercellular Communication Axes Between Deconvolved Cell Types (CellChat Analysis)

CellChat (v1.6.1) ligand-receptor interaction analysis was performed on spatially-defined Preserved and Rarefied regions to identify pathogenic intercellular signaling. This panel provides the quantitative data underlying the CellChat Circle Plot in Fig. 6j of the main text.

Signaling Pathway	Ligand (Source Cell Type)	Receptor (Target Cell Type)	Comm. Prob. Preserved	Comm. Prob. Rarefied	Fold Change (R/P)	P value	FDR
Profibrotic Signaling (Endothelial → Fibroblast, Upregulated in Rarefied)							
TGF-β	TGFB1 (Rarefied Endothelial)	TGFBR2 (Fibroblast / Myofibroblast)	0.08	0.42	5.25	<0.001	<0.001
TGF-β	TGFB1 (Macrophage)	TGFBR2 (Fibroblast / Myofibroblast)	0.06	0.38	6.33	<0.001	<0.001
PDGF	PDGFB (Rarefied Endothelial)	PDGFRB (Pericyte / vSMC)	0.04	0.22	5.50	<0.001	<0.001
CTGF / CCN2	CTGF (Fibroblast)	ITGB1/ITGA5 (Fibroblast, autocrine)	0.02	0.28	14.0	<0.001	<0.001
Proinflammatory Signaling (Pyroptotic Endothelial → Immune / Fibroblast, Upregulated in Rarefied)							
IL-1	IL1B (Pyroptotic Endothelial)	IL1R1 (Fibroblast / Myofibroblast)	0.02	0.36	18.0	<0.001	<0.001
IL-1	IL1B (Pyroptotic Endothelial)	IL1R1 (Macrophage)	0.02	0.32	16.0	<0.001	<0.001
IL-18	IL18 (Pyroptotic Endothelial)	IL18R1 (T / NK Cell)	0.01	0.18	18.0	<0.001	<0.001
CCL	CCL2 (Macrophage)	CCR2 (Monocyte, recruited)	0.04	0.34	8.50	<0.001	<0.001
CXCL	CXCL8 (Macrophage)	CXCR1/2 (Neutrophil, recruited)	0.02	0.16	8.00	<0.001	<0.001
Vascular Maintenance Signaling (Downregulated in Rarefied)							
ANGPT	ANGPT1 (Pericyte)	TEK/TIE2 (Endothelial)	0.38	0.06	0.16	<0.001	<0.001
VEGF	VEGFA (Hypoxic Tubular)	KDR/FLT1 (Endothelial)	0.42	0.08	0.19	<0.001	<0.001
NOTCH	DLL4 (Endothelial)	NOTCH1 (Endothelial, autocrine)	0.28	0.04	0.14	<0.001	<0.001

SEMA	SEMA3A (Podocyte)	NRP1 (Endothelial)	0.18	0.04	0.22	<0.001	<0.001
Senescence-Associated Secretory Phenotype (SASP, Upregulated in Rarefied)							
SERPINE1 (PAI-1)	SERPINE1 (Senescent Endothelial)	LRP1 (Fibroblast)	0.04	0.24	6.00	<0.001	<0.001
MMP	MMP2 (Fibroblast)	ITGB1 (Tubular Epithelial)	0.06	0.22	3.67	<0.001	<0.001
SPP1	SPP1 (Macrophage)	CD44 (Fibroblast)	0.04	0.26	6.50	<0.001	<0.001

Comm. Prob. = CellChat communication probability (0–1 scale; higher = stronger signaling). Fold Change = Rarefied / Preserved communication probability. Source and target cell types were identified by RCTD deconvolution; ligand and receptor expression was derived from the Visium gene expression data. The IL1B–IL1R1 axis (fold change 18.0) represents the strongest signaling increase, directly connecting endothelial pyroptosis (IL-1β release through GSDMD pores) to fibroblast activation. The VEGFA–KDR axis paradox (fold change 0.19) exemplifies "angiogenic paralysis": despite VEGFA upregulation by hypoxia (Table S5, rank #11 up), KDR receptor loss on surviving endothelial cells (Table S5, rank #2 down) effectively silences the pro-angiogenic signal. Fig. 6j in the main text shows the CellChat circle plot for the IL-1β/IL1R1 and TGFβ1/TGFBR2 axes specifically.

Supplementary Table S9

Complete Quantitative Data for Murine 2-Kidney-1-Clip (2K1C) Experiments Including MitoTEMPO Group

All data are presented as mean ± SEM (n = 8 per group unless otherwise noted). Young mice: 3 months old; Aged mice: 18 months old (C57BL/6J). Experiment 1 assessed the Age × Ischemia interaction (Figures 7–8). Experiment 2 assessed pharmacological rescue in Aged-2K1C mice (Figure 9 and this table). The MitoTEMPO group data, which are not presented in the main text figures, are shown here (orange shading) to complete the mechanistic chain: mitochondrial ROS → NLRP3 inflammasome → pyroptosis → microvascular rarefaction. MCC950 group data (green shading) correspond to Figure 9.

Panel A: Experiment 1 — Age × Ischemia Interaction (Figure 7)

Parameter	Young Sham (YS) n = 8	Young 2K1C (Y-2K1C) n = 8	Aged Sham (AS) n = 8	Aged 2K1C (A-2K1C) n = 8	Two-way ANOVA Interaction P (Age × Surgery)
Physiological Parameters					
Body weight at sacrifice, g	26.4 ± 0.8	25.8 ± 0.6	33.2 ± 1.4	31.6 ± 1.2	0.42
Right kidney weight, mg	182 ± 12	168 ± 14	196 ± 16	152 ± 18	0.03
Right kidney/body weight, mg/g	6.9 ± 0.4	6.5 ± 0.5	5.9 ± 0.4	4.8 ± 0.5	0.008
Systolic Blood Pressure (mmHg, Tail-Cuff) — Fig. 7b					
Baseline	108 ± 4	110 ± 5	118 ± 6	120 ± 5	0.82
Week 1	110 ± 5	132 ± 6	120 ± 6	148 ± 8	0.04
Week 2	112 ± 4	142 ± 8	122 ± 5	162 ± 8	0.02
Week 3	110 ± 5	148 ± 7	118 ± 6	168 ± 10	0.01
Week 4	108 ± 4	152 ± 8	120 ± 5	174 ± 10	0.006
CEUS Perfusion Parameters (Right Kidney)					
Peak Enhancement (PE), dB	22.4 ± 2.8	18.6 ± 2.4	20.2 ± 3.0	8.4 ± 2.2	<0.001
Time to Peak (TTP), s	4.2 ± 0.8	5.8 ± 1.0	4.8 ± 0.8	12.4 ± 2.6	<0.001
Wash-in Rate (WiR), dB/s	8.6 ± 1.4	5.8 ± 1.2	7.2 ± 1.6	2.2 ± 0.8	<0.001
Mean Transit Time (MTT), s	8.4 ± 1.6	12.2 ± 2.0	9.8 ± 1.8	22.6 ± 4.2	<0.001
Histology: Microvascular Density — Fig. 7d					
CD31+ area (% of cortical field)	7.2 ± 0.8	5.4 ± 0.6	6.4 ± 0.6	2.2 ± 0.4	<0.001
Histology: Fibrosis — Fig. 7e–f					
Fibrosis area (% Masson's trichrome)	2.4 ± 0.6	5.8 ± 1.2	4.2 ± 0.8	18.6 ± 2.8	<0.001
TEM: Mitochondrial Damage — Fig. 7f					

Mitochondrial damage score (0–3 scale)	0.4 ± 0.2	1.0 ± 0.3	0.8 ± 0.2	2.6 ± 0.3	<0.001
% endothelial cells with vacuolized mitochondria	4.2 ± 1.8	18.4 ± 4.2	12.6 ± 3.4	68.2 ± 8.4	<0.001
Western Blot Densitometry (Normalized to GAPDH, Fold vs. YS) — Fig. 7g					
NLRP3	1.0 ± 0.2	1.8 ± 0.4	1.4 ± 0.3	4.8 ± 0.6	<0.001
Cleaved Caspase-1 (p20)	1.0 ± 0.2	1.6 ± 0.3	1.2 ± 0.3	4.2 ± 0.5	<0.001
GSDMD-N	1.0 ± 0.1	1.4 ± 0.3	1.2 ± 0.2	3.8 ± 0.5	<0.001
qPCR (Fold Change vs. YS) — Fig. 7h					
Il1b mRNA	1.0 ± 0.2	1.5 ± 0.3	1.2 ± 0.2	4.0 ± 0.6	<0.001
Col1a1 mRNA	1.0 ± 0.2	1.2 ± 0.3	1.0 ± 0.2	5.0 ± 0.8	<0.001
Nlrp3 mRNA	1.0 ± 0.2	1.6 ± 0.3	1.2 ± 0.3	3.8 ± 0.5	<0.001
Gsdmd mRNA	1.0 ± 0.1	1.4 ± 0.2	1.2 ± 0.2	3.4 ± 0.4	<0.001
Casp1 mRNA	1.0 ± 0.2	1.4 ± 0.3	1.2 ± 0.2	3.6 ± 0.5	<0.001
Il18 mRNA	1.0 ± 0.2	1.2 ± 0.2	1.0 ± 0.2	2.8 ± 0.4	<0.001
Tgfb1 mRNA	1.0 ± 0.1	1.4 ± 0.3	1.2 ± 0.2	3.2 ± 0.4	<0.001
Acta2 mRNA	1.0 ± 0.2	1.2 ± 0.2	1.0 ± 0.2	3.6 ± 0.6	<0.001
Fn1 mRNA	1.0 ± 0.2	1.4 ± 0.3	1.2 ± 0.2	3.8 ± 0.5	<0.001
Serum Biomarkers					
Serum IL-1β, pg/mL	12.4 ± 3.2	28.6 ± 6.4	18.2 ± 4.8	86.4 ± 14.2	<0.001
Serum LDH, U/L	142 ± 18	186 ± 24	168 ± 22	324 ± 42	<0.001
Serum creatinine, μmol/L	14.2 ± 2.0	22.8 ± 3.4	16.4 ± 2.4	48.6 ± 8.2	<0.001
BUN, mmol/L	6.8 ± 1.0	10.2 ± 1.6	7.8 ± 1.2	18.4 ± 3.2	<0.001
Urinary NAG, U/L	4.2 ± 1.0	8.6 ± 2.0	5.4 ± 1.2	28.4 ± 5.6	<0.001

Data are mean ± SEM, n = 8 per group. Two-way ANOVA interaction P tests whether the effect of 2K1C surgery differs between Young and Aged mice. A significant interaction indicates that aging exacerbates ischemic injury beyond the additive effects of age and ischemia alone. All interaction P values for pyroptosis/fibrosis/microvascular endpoints are < 0.001, confirming the synergistic Age × Ischemia effect. Pairwise comparisons (Tukey HSD): YS vs. A-2K1C P < 0.001 for all parameters; Y-2K1C vs. A-2K1C P < 0.01 for CD31, fibrosis, all WB and qPCR markers. Blood pressure: A-2K1C achieves higher SBP than Y-2K1C (174 vs. 152 mmHg at week 4), consistent with more severe renovascular hypertension in aged mice. Mitochondrial damage score: 0 = normal cristae, 1 = mild swelling, 2 = moderate cristolysis, 3 = severe vacuolization/rupture (see Supplementary Methods S5.11).

Panel B: Experiment 2 — Pharmacological Intervention in Aged-2K1C Mice (Figure 9 + MitoTEMPO)

Orange shading: MitoTEMPO data (not shown in main text figures; presented here as Supplementary Figure S4 data). Green shading: MCC950 data (shown in Figure 9). Vehicle serves as the reference group for all comparisons.

Parameter	Aged Sham (AS) n = 8	Aged-2K1C + Vehicle n = 8	Aged-2K1C + MitoTEMPO n = 8	Aged-2K1C + MCC950 n = 8	P (one-way ANOVA)
Systolic Blood Pressure (mmHg) — Fig. 9b					
Baseline (pre-surgery)	118 ± 5	120 ± 6	118 ± 5	122 ± 6	0.82
Week 1	120 ± 6	146 ± 8	142 ± 7	144 ± 8	0.86 †
Week 2	118 ± 5	160 ± 8	154 ± 8	156 ± 8	0.72 †
Week 3	120 ± 6	168 ± 10	162 ± 9	164 ± 9	0.78 †
Week 4	118 ± 5	172 ± 10	168 ± 9	170 ± 10	0.92 †
CEUS Perfusion (Right Kidney, Week 4)					
Peak Enhancement (PE), dB	20.4 ± 2.8	8.2 ± 2.0	12.8 ± 2.4 *	16.2 ± 2.6 **	<0.001
Time to Peak (TTP), s	4.6 ± 0.8	12.8 ± 2.8	9.4 ± 2.0 *	6.8 ± 1.4 **	<0.001
Wash-in Rate (WiR), dB/s	7.4 ± 1.4	2.0 ± 0.6	3.8 ± 1.0 *	5.4 ± 1.2 **	<0.001
Mean Transit Time (MTT), s	10.2 ± 1.8	22.8 ± 4.0	16.4 ± 3.2 *	12.6 ± 2.4 **	<0.001
Histology: Microvascular Density — Fig. 9c–d					
CD31+ area (% of cortical field)	6.2 ± 0.6	2.0 ± 0.4	3.6 ± 0.5 **	5.2 ± 0.6 ***	<0.001
CD31+ capillaries per HPF	32.4 ± 4.2	10.8 ± 2.4	18.6 ± 3.2 **	26.8 ± 3.8 ***	<0.001
Histology: Fibrosis — Fig. 9g–h					
Fibrosis area (%)	4.4 ± 0.8	19.2 ± 2.6	12.4 ± 2.0 **	7.8 ± 1.4 ***	<0.001
TEM: Mitochondrial Ultrastructure					
Mitochondrial damage score (0–3)	0.8 ± 0.2	2.6 ± 0.3	1.2 ± 0.3 ***	2.2 ± 0.3 (NS)	<0.001
% EC with vacuolized mito.	12.8 ± 3.2	66.4 ± 8.2	24.6 ± 5.8 ***	52.8 ± 7.4 (NS)	<0.001
MitoSOX (Mitochondrial Superoxide, Fold vs. AS)					
MitoSOX intensity (cortex IF)	1.0 ± 0.2	5.8 ± 0.8	2.2 ± 0.4 ***	4.8 ± 0.6 (NS)	<0.001
Western Blot Densitometry (Fold vs. AS) — Fig. 9e					
NLRP3	1.0 ± 0.2	4.6 ± 0.6	2.8 ± 0.4 **	1.4 ± 0.3 ***	<0.001
Cleaved Caspase-1 (p20)	1.0 ± 0.2	4.0 ± 0.5	2.4 ± 0.4 **	1.2 ± 0.2 ***	<0.001
GSDMD-N	1.0 ± 0.1	3.6 ± 0.4	2.2 ± 0.3 **	1.2 ± 0.2 ***	<0.001
qPCR (Fold vs. AS) — Fig. 9f					
Il1b mRNA	1.0 ± 0.2	10.2 ± 1.4	5.4 ± 0.8 **	2.8 ± 0.4 ***	<0.001
Col1a1 mRNA	1.0 ± 0.2	10.4 ± 1.6	6.2 ± 1.0 **	3.2 ± 0.6 ***	<0.001
Nlrp3 mRNA	1.0 ± 0.2	3.6 ± 0.5	2.2 ± 0.3 **	1.4 ± 0.2 ***	<0.001

Tgfb1 mRNA	1.0 ± 0.1	3.4 ± 0.4	2.2 ± 0.3 **	1.4 ± 0.2 ***	<0.001
Acta2 mRNA	1.0 ± 0.2	3.8 ± 0.6	2.4 ± 0.4 **	1.6 ± 0.3 ***	<0.001
Serum and Urinary Biomarkers — Fig. 9h					
Serum IL-1β, pg/mL	18.4 ± 4.6	88.2 ± 12.8	48.4 ± 8.2 **	24.6 ± 5.2 ***	<0.001
Serum LDH, U/L	172 ± 22	328 ± 38	224 ± 28 **	192 ± 24 ***	<0.001
Serum creatinine, μmol/L	16.8 ± 2.6	46.2 ± 7.8	32.4 ± 5.4 *	22.8 ± 3.8 **	<0.001
BUN, mmol/L	8.0 ± 1.2	18.8 ± 3.0	13.2 ± 2.4 *	10.4 ± 1.8 **	<0.001
Urinary NAG, U/L	5.6 ± 1.4	26.8 ± 4.8	16.2 ± 3.2 *	8.8 ± 2.0 ***	<0.001
Urinary albumin/creatinine ratio, μg/mg	42 ± 12	286 ± 48	168 ± 32 **	82 ± 18 ***	<0.001

*Data are mean ± SEM. * P < 0.05, ** P < 0.01, *** P < 0.001 vs. Vehicle (Tukey HSD post hoc). (NS) = not significant vs. Vehicle. †Blood pressure comparisons among the three 2K1C groups only (excluding Aged Sham); P values confirm that neither MitoTEMPO nor MCC950 significantly lowered blood pressure, indicating that their renoprotective effects are independent of hemodynamic changes. Key mechanistic dissociation: MitoTEMPO significantly reduces MitoSOX intensity (5.8 → 2.2, P < 0.001) and mitochondrial damage score (2.6 → 1.2, P < 0.001) but only partially reduces NLRP3/Casp-1/GSDMD-N (40–50% reduction). MCC950 does NOT reduce MitoSOX intensity (5.8 → 4.8, NS) or mitochondrial damage (2.6 → 2.2, NS) but effectively blocks the downstream pyroptosis cascade (NLRP3/Casp-1/GSDMD-N reduced by 65–75%). This pharmacological dissection confirms the causal hierarchy: mtROS → NLRP3 → pyroptosis.*

Panel C: Mechanistic Dissection — MitoTEMPO vs. MCC950 Effect Profiles

Side-by-side comparison of the two pharmacological agents to demonstrate their distinct mechanisms of action and confirm the causal directionality of the mitochondrial ROS → NLRP3 inflammasome → pyroptosis axis.

Endpoint	Vehicle (ref)	Mito-TEMPO	MCC950	% Rescue Mito-TEMPO	% Rescue MCC950	P (MT vs. MCC)	Interpretation
Upstream: Mitochondrial Function							
MitoSOX intensity	5.8	2.2	4.8	75%	17% (NS)	<0.001	MitoTEMPO scavenges mtROS; MCC950 does not act at mito. level
Mito. damage score	2.6	1.2	2.2	78%	22% (NS)	<0.001	MitoTEMPO preserves mito. ultrastructure; MCC950 does not
Midstream: NLRP3 Inflammasome							
NLRP3 protein	4.6	2.8	1.4	50%	89%	0.008	Both reduce; MCC950 more effective at NLRP3 level
Cleaved Casp-1 (p20)	4.0	2.4	1.2	53%	93%	0.004	MCC950 blocks Casp-1 cleavage more completely
Downstream: Pyroptotic Execution							
GSDMD-N	3.6	2.2	1.2	54%	92%	0.006	MCC950 blocks pore formation nearly completely
Serum IL-1β	88.2	48.4	24.6	57%	91%	0.002	MCC950 blocks IL-1β release more effectively
LDH release	328	224	192	67%	87%	0.04	Both reduce cell death; MCC950 slightly better
Functional Outcomes							
CD31+ area	2.0	3.6	5.2	38%	76%	0.01	MCC950 preserves more microvasculature
Fibrosis area	19.2	12.4	7.8	46%	77%	0.008	MCC950 more anti-fibrotic

Serum Cr.	46.2	32.4	22.8	47%	80%	0.02	MCC950 better renal function preservation
Urinary NAG	26.8	16.2	8.8	50%	85%	0.006	MCC950 more tubulo- protective

% Rescue = [(Vehicle − Treatment) / (Vehicle − Aged Sham)] × 100%, representing the proportion of disease rescued toward the Aged Sham baseline. P (MT vs. MCC): direct comparison between MitoTEMPO and MCC950 groups (unpaired t-test). The data reveal a clear mechanistic hierarchy: MitoTEMPO is superior for upstream endpoints (mtROS, mitochondrial ultrastructure: 75–78% rescue) but only partially effective at downstream endpoints (NLRP3/Casp-1/GSDMD-N: 50–54% rescue). Conversely, MCC950 is ineffective upstream (17–22% rescue of mtROS/mito damage, NS) but highly effective downstream (89–93% rescue of inflammasome/pyroptosis). Both converge on functional outcomes (CD31, fibrosis, creatinine), but MCC950 is significantly more effective (76–85% vs. 38–50% rescue). This demonstrates that: (1) mtROS is the Signal 2 that triggers NLRP3; (2) blocking NLRP3 downstream is more effective than scavenging ROS upstream, because additional NLRP3-activating signals (e.g., DAMPs, K+ efflux) exist beyond mtROS; (3) NLRP3 is the critical bottleneck and optimal therapeutic target.

Panel D: In Vitro Data — HRGEC Hypoxia/Reoxygenation Model (Figure 8)

Parameter	Young Ctrl (Y-Ctrl) n = 6	Young H/R (Y-H/R) n = 6	Aged Ctrl (A-Ctrl) n = 6	Aged H/R (A-H/R) n = 6	P (two-way ANOVA interaction)	Figure
Senescence Validation — Fig. 8b						
SA-β-gal+ cells, %	3.2 ± 1.4	—	72.4 ± 6.8	—	N/A	8b
Population doubling time, days	2.8 ± 0.4	—	>14	—	N/A	8b
Mitochondrial Function — Fig. 8c–d						
MitoSOX intensity (fold vs. Y-Ctrl)	1.0 ± 0.2	2.2 ± 0.4	1.8 ± 0.3	6.4 ± 0.8	<0.001	8c
JC-1 red/green ratio (fold vs. Y-Ctrl)	1.0 ± 0.1	0.72 ± 0.08	0.82 ± 0.10	0.28 ± 0.06	<0.001	8d
Cytosolic mtDNA (fold vs. Y-Ctrl)	1.0 ± 0.2	2.8 ± 0.6	2.2 ± 0.4	12.4 ± 1.8	<0.001	8d
Western Blot (Fold vs. Y-Ctrl) — Fig. 8e						
NLRP3	1.0 ± 0.2	2.0 ± 0.4	1.6 ± 0.3	5.2 ± 0.6	<0.001	8e
Pro-Caspase-1	1.0 ± 0.1	1.2 ± 0.2	1.0 ± 0.2	1.4 ± 0.2	0.42	8e
Cleaved Caspase-1 (p20)	1.0 ± 0.2	1.8 ± 0.3	1.4 ± 0.3	4.8 ± 0.6	<0.001	8e
GSDMD-FL	1.0 ± 0.1	1.0 ± 0.2	1.0 ± 0.1	0.6 ± 0.1	0.02	8e
GSDMD-N	1.0 ± 0.1	1.6 ± 0.3	1.4 ± 0.2	4.2 ± 0.5	<0.001	8e
LDH Release (%) — Fig. 8h						
LDH release, %	4.2 ± 1.2	12.8 ± 2.4	8.4 ± 1.8	42.6 ± 5.8	<0.001	8h

In Vitro Pharmacological Rescue (A-H/R Group) — Fig. 8g–h

Parameter	A-H/R + Vehicle n = 6	A-H/R + MitoQ (500 nM) n = 6	A-H/R + MCC950 (10 μM) n = 6	P (ANOVA)	Figure
Mitochondrial ROS					
MitoSOX intensity (fold vs. Y-Ctrl)	6.4 ± 0.8	2.4 ± 0.4 ***	5.6 ± 0.6 (NS)	<0.001	Suppl.
Western Blot (Fold vs. Y-Ctrl) — Fig. 8g					
Cleaved Casp-1 (p20)	4.8 ± 0.6	2.6 ± 0.4 **	1.4 ± 0.3 ***	<0.001	8g
GSDMD-N	4.2 ± 0.5	2.4 ± 0.4 **	1.2 ± 0.2 ***	<0.001	8g
Cell Death — Fig. 8h					
LDH release, %	42.6 ± 5.8	22.4 ± 3.6 **	16.8 ± 2.8 ***	<0.001	8h

*Data are mean ± SEM from n = 6 independent biological replicates. * P < 0.05, ** P < 0.01, *** P < 0.001 vs. Vehicle (Tukey HSD).*

(NS) = not significant vs. Vehicle. Y-Ctrl = Young Control (normoxia); A-H/R = Aged Hypoxia/Reoxygenation (1% O_2 24h / 21% O_2 6h). MitoQ (500 nM) = mitochondria-targeted antioxidant; MCC950 (10 μM) = selective NLRP3 inhibitor. Pro-Caspase-1 levels do not change significantly (interaction P = 0.42), indicating that pyroptosis activation occurs at the post-translational cleavage step, not transcriptional upregulation of the pro-form. GSDMD-FL decreases in A-H/R (0.6-fold), consistent with its cleavage into the active GSDMD-N fragment (which increases 4.2-fold). The in vitro MitoQ/MCC950 dissection pattern mirrors the in vivo MitoTEMPO/MCC950 results (Panel C), providing cross-platform validation of the mechanistic hierarchy.

Panel E: Cross-Reference with Main Text Figures

Figure	Panel Description	Table S9 Panel	Key Data Point (Table S9 → Figure Concordance)
Figure 7: Age × Ischemia			
Fig. 7b	Systolic BP time course	A	Week 4: A-2K1C 174 mmHg vs. Y-2K1C 152 mmHg. Interaction P = 0.006.
Fig. 7d	CD31 immunofluorescence	A	CD31+ area: YS 7.2% → A-2K1C 2.2%. Interaction P < 0.001.
Fig. 7e–f	Trichrome + TEM	A	Fibrosis: YS 2.4% → A-2K1C 18.6%. Mito damage: YS 0.4 → A-2K1C 2.6.
Fig. 7g	Western blot NLRP3/Casp1	A	NLRP3: 4.8-fold; p20: 4.2-fold; GSDMD-N: 3.8-fold (all vs. YS).
Fig. 7h	qPCR Il1b / Col1a1	A	Il1b: 4.0-fold; Col1a1: 5.0-fold (A-2K1C vs. YS).
Figure 8: Mitochondrial-Pyroptosis Axis			
Fig. 8b	SA-β-gal	D	Young 3.2% → Aged 72.4% positive. Confirms senescence.
Fig. 8c	MitoSOX	D	Y-Ctrl 1.0 → A-H/R 6.4-fold. Interaction P < 0.001.
Fig. 8d	JC-1 + cytosolic mtDNA	D	JC-1 ratio: 1.0 → 0.28; cytosolic mtDNA: 1.0 → 12.4-fold.
Fig. 8e	Full WB panel	D	NLRP3 5.2×; Casp-1 p20 4.8×; GSDMD-N 4.2× (A-H/R vs. Y-Ctrl).
Fig. 8g	Rescue WB (MitoQ/MCC950)	D	MitoQ: Casp-1 p20 2.6×; MCC950: 1.4× (both vs. Vehicle 4.8×).
Fig. 8h	LDH rescue	D	Vehicle 42.6% → MitoQ 22.4% → MCC950 16.8%.
Figure 9: MCC950 In Vivo Rescue			
Fig. 9b	BP time course	B	MCC950 does not lower BP (170 vs. Vehicle 172 mmHg, NS).
Fig. 9c–d	CD31 + quantification	B	CD31+ area: Vehicle 2.0% → MCC950 5.2% (*** P < 0.001).
Fig. 9e	Western blot	B	NLRP3: Vehicle 4.6 → MCC950 1.4; GSDMD-N: 3.6 → 1.2.
Fig. 9f	qPCR Il1b / Col1a1	B	Il1b: Vehicle 10.2 → MCC950 2.8; Col1a1: 10.4 → 3.2.
Fig. 9g–h	Trichrome + NAG	B	Fibrosis: Vehicle 19.2% → MCC950 7.8%; NAG: 26.8 → 8.8 U/L.
Supplementary Figure S4: MitoTEMPO In Vivo (NEW DATA)			
Suppl. Fig. S4a	CEUS perfusion	B	PE: Vehicle 8.2 → MitoTEMPO 12.8 dB (*); TTP: 12.8 → 9.4 s (*).
Suppl. Fig. S4b	CD31 IF	B	CD31+ area: Vehicle 2.0% → MitoTEMPO 3.6% (**).
Suppl. Fig. S4c	MitoSOX cortex	B	MitoSOX: Vehicle 5.8 → MitoTEMPO 2.2-fold (***).
Suppl. Fig. S4d	Western blot	B	NLRP3: Vehicle 4.6 → MitoTEMPO 2.8 (**); GSDMD-N: 3.6 → 2.2 (**).
Suppl. Fig. S4e	Fibrosis + biomarkers	B	Fibrosis: 19.2% → 12.4% (**); NAG: 26.8 → 16.2 U/L (*).

This cross-reference matrix enables reviewers to trace every quantitative claim in the main text figures to its source data in this table. The last section (Supplementary Figure S4) provides the figure panel mapping for the MitoTEMPO in vivo data that are new to this supplementary material. All data points in Panels A–D are consistent with the main text figure annotations within rounding precision.

Supplementary Table S10

STARD-AI, TRIPOD+AI, and ARRIVE 2.0 Reporting Checklists

Completed checklists for the three reporting guidelines relevant to this study. Green = fully reported. Yellow = partially reported. Section references point to main text or supplementary materials. References: STARD-AI (Sounderajah et al., Nature Medicine 2025); TRIPOD+AI (Collins et al., BMJ 2024; 385: e078378); ARRIVE 2.0 (Percie du Sert et al., PLoS Biology 2020; 18: e3000410).

Checklist 1: STARD-AI (Standards for Reporting of Diagnostic Accuracy Studies — Artificial Intelligence)

Applicable to the Renal-Video-AI phenotype classification and its diagnostic/prognostic accuracy evaluation.

Item No.	STARD-AI Checklist Item	Reported?	Section / Location	Comment
TITLE AND ABSTRACT				
1	Identify as a study of diagnostic accuracy using AI	Yes	Title, Abstract	Title includes "Deep Video-Phenomapping"; Abstract describes AI diagnostic framework
2	Provide structured summary including index test, reference standard, findings	Yes	Abstract	Structured abstract with Background/Methods/Results/Conclusions
INTRODUCTION				
3	Scientific and clinical background, including intended use and clinical role of AI	Yes	Introduction §1–4	Clinical gap (ARAS stenting paradox), intended use (phenotype-guided revascularization)
4	Study objectives and hypotheses	Yes	Introduction §5	Three-layer triangulation hypothesis clearly stated
METHODS — Study Design				
5	Whether data collection was planned before or after AI development (prospective vs. retrospective)	Yes	Methods 2.2	Discovery = retrospective; Validation = independent; Multimodal = prospective
6	Eligibility criteria	Yes	Methods 2.2.1–2.2.3	Age ≥45, ARAS ≥50%, CEUS available; exclusion criteria detailed
7	On what basis potentially eligible participants were identified	Yes	Methods 2.2.1	Multi-center registry, Jan 2015–Dec 2024, 7 tertiary centers
METHODS — AI System (Index Test)				
8	Technology description including architecture, inputs, outputs	Yes	Methods 2.3; Supp. S1–S2	Video Swin Transformer + VideoMAE; input = CEUS video; output = 1024-d features
9	Pre-processing of data before input to AI	Yes	Methods 2.3.1; Supp. S1	B-Spline motion correction; 224×224 resize; min-max normalization

10	Training procedure including SSL, hyperparameters	Yes	Supp. S2.3–S2.4	VideoMAE 90% masking, 800 epochs, AdamW, cosine schedule
11	Whether AI output was used as sole test or combined with clinical information	Yes	Methods 2.6; Results 3.3; Table 4	AI phenotype tested alone (Model 1) and combined with clinical variables (Model 4)
12	Training/validation/test data split strategy	Yes	Methods 2.2.1–2.2.2	Discovery (N=1226) / External Validation (N=122) / Multimodal (N=57)
METHODS — Reference Standard				
13	Reference standard and its rationale	Yes	Methods 2.4; Methods 2.6	Spatial transcriptomics (biological ground truth); MARE composite endpoint (clinical)
14	How reference standard was applied	Yes	Methods 2.4; Supp. S3	10x Visium within <48h of CEUS; MARE adjudicated from clinical records
METHODS — Analysis				
15	Handling of missing data, indeterminate results	Partial	Methods 2.3.1	Registration failures excluded (3.2%); missing data handling not detailed for clinical variables
16	How AI failure/errors were handled	Yes	Supp. S1.7	Registration failure rate 3.2% (39/1226); visual QC by two sonographers
17	Measures of diagnostic accuracy and precision estimates	Yes	Results 3.3; Table 4–5	C-statistic, NRI, IDI, HR with 95% CI; time-dependent AUC
18	Subgroup analyses	Yes	Results 3.3; Table 3; Supp. S3	Subgroup forest plot (Fig 4C); 20+ sensitivity analyses in Table S3
METHODS — AI-Specific Items				
19	Data augmentation strategy	Yes	Supp. S2.1	Temporal jitter, spatial crop, horizontal flip, intensity scaling
20	Overfitting mitigation strategy	Yes	Methods 2.3.3; Supp. S2.3–S2.4	SSL pre-training (no labels); external validation; LOCO cross-validation (Table S3)
21	Explainability / interpretability methods	Yes	Methods 2.4.1; Results 3.4	Grad-CAM++ attention maps; spatial registration with transcriptomics
22	Software, hardware, and computational requirements	Yes	Supp. S1.7, S2.4	4×A100 GPUs; PyTorch 2.0; ~72h pre-training; SimpleITK; R/Python
RESULTS				
23	Flow of participants (flow diagram)	Partial	Results 3.1; Fig. 1	Study design overview in Fig 1; formal STARD flow diagram recommended
24	Baseline demographics of study participants	Yes	Table 1; Table S1	Full baseline by phenotype (Table 1); validation cohorts (Table S1)
25	Distribution of AI outputs	Yes	Results 3.2; Fig. 3A	UMAP distribution; cluster sizes 510/435/281
26	Cross-tabulation or ROC curves	Yes	Table 4–5; Fig. 4D; Table S3	C-statistics at multiple timepoints; ROC in Suppl.; calibration in Table S3F
27	Estimates of accuracy with precision (CI)	Yes	Table 3–5; Table S3	All HRs with 95% CI; C-statistic with CI; NRI/IDI with CI
28	Adverse events from AI use	Yes	N/A	Not applicable (diagnostic, non-interventional AI

				system)
DISCUSSION				
29	Study limitations including applicability concerns	Yes	Discussion Limitations	Retrospective design, Visium resolution, 2K1C model limitations
30	Implications for practice including deployment pathway	Yes	Discussion §1–4; Fig. 4F	Traffic Light decision model; precision medicine tool for stenting guidance

Green shading = fully reported. Yellow = partially reported (action needed). Item 15: recommend adding a brief statement on missing data handling for clinical covariates in the statistical methods. Item 23: recommend adding a formal STARD participant flow diagram as a supplementary figure.

Checklist 2: TRIPOD+AI (Transparent Reporting of a Multivariable Prediction Model for Individual Prognosis or Diagnosis + Artificial Intelligence)

Applicable to the development of the Renal-Video-AI risk prediction model (Tables 3–4) and its external validation (Table 5). Checklist based on Collins et al., BMJ 2024; 385: e078378.

Item No.	TRIPOD+AI Checklist Item	Reported?	Section / Location	Comment
TITLE AND ABSTRACT				
1	Informative title: identify as prediction model study, target population, outcome	Yes	Title	Identifies AI phenomapping, target population (aging RAS), and outcome (rarefaction)
2	Structured abstract with objectives, methods, results, conclusions	Yes	Abstract	Structured abstract with all required elements
INTRODUCTION				
3	Healthcare context, rationale, and intended use of model	Yes	Introduction §1–4	ARAS clinical conundrum; AI to guide revascularization decisions
4	Study objectives: development, validation, or both	Yes	Introduction §5	Both development (Discovery) and external validation
METHODS — Source of Data				
5	Describe setting, locations, dates of data collection	Yes	Methods 2.2.1–2.2.3	7 centers; Jan 2015–Dec 2024; 6 validation centers
6	Eligibility criteria, treatment received	Yes	Methods 2.2; Table 1	Age≥45, ARAS≥50%, stenting vs. medical in Table 1
7	Differences between development and validation data	Yes	Methods 2.2.2; Table S1C	Different vendors; geographic diversity; comparability in Table S1 Panel C
METHODS — Outcome				
8	Outcome definition, timing, blinding of assessment	Yes	Methods 2.6	MARE composite; 3.5-year median follow-up; competing risk of CV death addressed
METHODS — Predictors				
9	All predictors, including how and when measured	Yes	Methods 2.3; Table 2; Supp. S2	1024-d video features; CEUS acquisition protocol; conventional clinical variables
10	AI model: architecture, inputs, outputs, pre-processing	Yes	Methods 2.3; Supp. S1–S2	Video Swin Transformer Swin-B; VideoMAE; motion correction pipeline
11	Feature engineering and selection method	Yes	Methods 2.3.4; Supp. S2.6	Unsupervised UMAP + Leiden clustering; no manual feature engineering
METHODS — Sample Size				
12	How sample size was determined	Partial	Methods 2.2	N=1226 available patients; formal sample size calculation not performed for AI development
METHODS — Missing Data				
13	How missing data were handled	Partial	Methods 2.3.1; Supp. S1.7	Registration failures excluded; recommend stating

				clinical variable missingness
METHODS — Analysis				
14	Type of model, model-building procedure, internal validation	Yes	Methods 2.3.2–2.3.4; Supp. S2	SSL pre-training; unsupervised clustering; 5-fold CV for risk score; LOCO in Table S3
15	Performance measures: discrimination, calibration	Yes	Table 4–5; Table S3E–F	C-statistic, NRI, IDI (discrimination); Hosmer-Lemeshow, Brier, calibration slope
16	Model comparison methods	Yes	Table 4; Table S4	Stepwise models; head-to-head vs. RI, TIC, clinical model; DeLong test for ΔC
17	Fairness and subgroup evaluation	Yes	Table 3; Table S3B	Subgroup analyses by age, sex, DM, CKD stage, stenosis severity; interaction P
RESULTS				
18	Participant characteristics including demographics	Yes	Table 1; Table S1	Full baseline stratified by phenotype; three cohorts compared
19	Model performance with CIs	Yes	Table 3–5; Table S3	C-statistic 0.88 (0.85–0.91); NRI 0.45 (0.32–0.58); all HRs with CI
20	Calibration results including plots	Yes	Table 5; Table S3F	H-L P, Brier, calibration slope, E/O ratio at 1–5 years
21	Model updating / recalibration if applicable	N/A	N/A	No model updating performed (first report of this model)
DISCUSSION				
22	Limitations including overfitting, generalizability	Yes	Discussion Limitations	Retrospective bias, Visium resolution, animal model limitations; LOCO validates generalizability
23	Implications for clinical use	Yes	Discussion; Fig. 4F	Traffic Light model; NNT=10 in Cluster 2 (Table S2F)
OTHER INFORMATION				
24	Study registration	Partial	Methods 2.1	IRB approvals listed; trial registration number not applicable (not a trial)
25	Model availability: code, weights, access	Yes	Data Availability Statement	Code on GitHub; model weights available upon request; GEO/GSA for transcriptomics
26	Funding and COI	Yes	Back Matter	Funding section; COI declaration (see Back Matter document)
27	Reporting guideline used	Yes	Methods 2.1; This table	STARD-AI and TRIPOD-AI stated in Methods; checklists provided

Item 12: For AI/ML studies, formal a priori sample size calculations are rarely feasible due to the complexity of the learning task. The study reports a pragmatic approach (all available patients meeting eligibility). The large N=1,226 with 268 events provides adequate effective sample size. Item 13: Recommend adding a statement on clinical variable completeness in Methods 2.6.

Checklist 3: ARRIVE 2.0 (Animal Research: Reporting of In Vivo Experiments)

Applicable to Figures 7–9 (2K1C murine model) and Supplementary Table S9. Checklist based on Percie du Sert et al., PLoS Biology 2020; 18: e3000410.

Item No.	ARRIVE 2.0 Essential Item	Reported?	Section / Location	Comment
ESSENTIAL 10 (Must Be Reported)				
1	Study design: groups, experimental unit, controls	Yes	Methods 2.5; Supp. S5.3	2 experiments; 4 groups (Exp 1) + 3 groups (Exp 2); mouse as unit
2	Sample size: a priori calculation, n per group	Yes	Supp. S5.2	G*Power calculation; n=8/group; based on CD31+ pilot data
3	Inclusion/exclusion criteria for animals	Yes	Supp. S5.1; S5.14	Male C57BL/6J; age 3 mo / 18 mo; humane endpoints defined
4	Randomisation: method, who performed it	Yes	Supp. S5.4	Computer-generated random numbers; block randomization for Exp 2; Y.W. performed
5	Blinding: at allocation, conduct, assessment	Yes	Supp. S5.4	4-layer blinding: surgeon unblinded but sham-controlled; CEUS, histology, WB all blinded
6	Outcome measures: primary and secondary, timing	Yes	Methods 2.5; Supp. S5.9– S5.13; Table S9	Primary: CD31+ area; secondary: fibrosis, WB, qPCR, serum biomarkers; all at week 4
7	Statistical methods: tests, software, multiplicity	Yes	Methods 2.6; Supp. S5.16	One-way ANOVA + Tukey; two-way ANOVA for interaction; GraphPad Prism v10.0
8	Experimental animals: species, strain, sex, age, weight, source	Yes	Methods 2.5.1; Supp. S5.1	Male C57BL/6J; 3 mo (~25g) and 18 mo (~32g); Beijing Vital River
9	Experimental procedures: surgery, drugs, doses, routes, timing	Yes	Methods 2.5; Supp. S5.5– S5.7	2K1C 0.12mm clip; MitoTEMPO 0.7 mg/kg/d i.p.; MCC950 10 mg/kg q.o.d. i.p.; 4 weeks
10	Results: effect sizes, CIs or SEM, exact P values, n for each group	Yes	Table S9 Panels A–D	All data as mean±SEM, n=8; exact P from ANOVA; interaction P reported
RECOMMENDED ITEMS				
11	Abstract: species, key methods, principal findings	Yes	Abstract	Aged murine 2K1C model mentioned; MCC950 rescue described
12	Background: literature review, relevance	Yes	Introduction §4	Inflammaging, mitochondrial ROS, NLRP3; 2K1C model context
13	Objectives: primary hypothesis for animal experiments	Yes	Introduction §5	Causal role of mitochondrial-pyroptosis axis in aging rarefaction
14	Ethical statement: IACUC approval, guidelines	Yes	Methods 2.1; Back Matter	IACUC approval number; NIH Guide; ARRIVE 2.0 compliance stated
15	Housing and husbandry: facility, light cycle, diet, water	Yes	Supp. S5.1	SPF; 12h light/dark; standard chow; autoclaved water; 22±2°C
16	Animal care: anesthesia, analgesia, monitoring	Yes	Supp. S5.5	Isoflurane; buprenorphine post-op; daily monitoring 7 days

17	Euthanasia method	Yes	Supp. S5.8	Cervical dislocation under deep isoflurane anesthesia
18	Interpretation: biological relevance, translation to human	Yes	Discussion §3–4	Aged mice mimic Cluster 3; MCC950 as translational target
19	Generalizability: limitations of the model	Yes	Discussion Limitations	2K1C vs. atherosclerotic RAS; no cholesterol emboli; $ApoE^{-/-}$ suggested
20	Funding: source and role	Yes	Back Matter	Funding section with funder role statement
21	Conflicts of interest	Yes	Back Matter	COI declaration provided
ADDITIONAL REPORTING DETAILS				
22	Number of animals excluded and reasons	Yes	Supp. S5.3; S5.14	6/62 surgical mortality (replaced); 2 humane endpoint in Vehicle group
23	Adverse events during experiment	Yes	Supp. S5.14	2 aged Vehicle mice reached >20% weight loss endpoint at weeks 3–4
24	Baseline data for each group	Yes	Table S9A	Body weight, baseline BP for all 4 groups
25	Replicates: biological vs. technical	Yes	Supp. S5.12; S5.13; Table S9	n=8 biological replicates; WB densitometry on independent samples; qPCR in triplicate
26	Protocol registration	Partial	N/A	Animal protocol registered with IACUC but no public registry (e.g., preclinicaltrials.eu)
27	Data availability for animal experiments	Yes	Data Availability; Table S9	Complete quantitative data in Table S9; raw images available upon request
28	Welfare assessment: grimace scale or scoring	Yes	Supp. S5.14	Mouse Grimace Scale ≥2 as humane endpoint criterion
29	Inter-observer reliability	Yes	Supp. S5.4	ICC >0.90 for CD31, fibrosis, TEM scoring; two blinded observers
30	Full Western blot images	Yes	Supp. Fig. S5	Full uncropped blots with molecular weight markers for all panels

Item 26: Public pre-registration of animal protocols (e.g., at preclinicaltrials.eu or OSF) is increasingly encouraged but not yet mandatory for most journals. Consider prospective registration for future animal studies. All other ARRIVE 2.0 essential items (1–10) are fully reported.

Compliance Summary Across Three Checklists

Checklist	Total Items	Fully Reported	Partially Reported	N/A	Compliance Rate
STARD-AI	30	27	2	1	90% (27/30) 97% (29/30 incl. partial)
TRIPOD+AI	27	23	3	1	85% (23/27) 96% (26/27 incl. partial)
ARRIVE 2.0 (Essential 10)	10	10	0	0	100% (10/10)
ARRIVE 2.0 (All 30)	30	28	1	1	93% (28/30) 97% (29/30 incl. partial)
Combined (All 87 items)	87	78	5	3	90% (78/87) 95% (83/87 incl. partial)

Supplementary Table S11.

Multivariable logistic-regression predictors of 3-year event-free survival among stented patients.

S11A. All stented patients (n = 395).

Predictor	Adjusted OR	95% CI	P
Delayed phenotype (vs Rarefied)	3.85	2.10–7.06	<0.001
Preserved phenotype (vs Rarefied)	3.42	1.82–6.43	<0.001
PTC-EC fraction (per +10%)	2.18	1.46–3.25	<0.001
Baseline proteinuria (per +1 g/g)	0.52	0.38–0.71	<0.001
CEUS time-to-peak (per +5 s)	0.74	0.61–0.90	0.002
AI Perfusion Risk Score (per +0.1)	0.69	0.58–0.82	<0.001
eGFR (per +10 mL/min/1.73 m²)	1.12	0.95–1.32	0.18
Anatomical stenosis severity (per +10%)	1.05	0.90–1.23	0.53
Age (per +10 y)	0.88	0.71–1.09	0.24
Male sex	0.93	0.62–1.40	0.74
Diabetes mellitus	0.81	0.55–1.19	0.28

S11B. Within the Delayed phenotype (n = 168).

Predictor	Adjusted OR	95% CI	P
PTC-EC fraction (per +10%)	2.04	1.22–3.41	0.006
Baseline proteinuria (per +1 g/g)	0.57	0.39–0.83	0.004
CEUS time-to-peak (per +5 s)	0.78	0.62–0.98	0.034
AI Perfusion Risk Score (per +0.1)	0.66	0.52–0.84	<0.001

OR = odds ratio; PTC-EC = peritubular-capillary endothelial cell. All-stented model C-statistic 0.84 (optimism-corrected 0.82).

The number of stented patients (395 = 142 Preserved + 168 Delayed + 85 Rarefied) reconciles with Table 1 and Table 3D.

Supplementary Table S12.

Covariate balance before and after 1:1 propensity-score matching (caliper 0.2 SD; 354 matched pairs).

Covariate	Stenting (matched)	Medical (matched)	SMD before	SMD after
Age (years)	68.2 ± 8.4	68.6 ± 8.1	0.18	0.05
Male sex (%)	61.3	59.6	0.12	0.04
eGFR (mL/min/1.73 m²)	48.6 ± 15.2	49.1 ± 14.8	0.21	0.03
Systolic blood pressure (mmHg)	152 ± 18	150 ± 17	0.15	0.06
Peak systolic velocity ratio	3.4 ± 0.9	3.3 ± 0.8	0.24	0.07
Anatomical stenosis severity (%)	71.4 ± 12.6	70.2 ± 13.1	0.28	0.08
Diabetes mellitus (%)	38.1	36.4	0.14	0.04
Prior cardiovascular events (%)	32.5	31.1	0.11	0.03
No. of antihypertensive agents	2.8 ± 1.1	2.7 ± 1.0	0.19	0.06

SMD = standardized mean difference; mean ± SD or %. Propensity scores estimated within each phenotype. After matching all SMDs < 0.10. Matched pairs: Preserved 128, Delayed 152, Rarefied 74 (total 354). Matched treatment effect: Preserved HR 0.94, Delayed HR 0.51 (95% CI 0.33–0.79), Rarefied HR 1.07; interaction P = 0.01 (consistent with Fig S11 and Results §3.3).

Supplementary Table S13.

Editorial and statistical-formatting standardisation changes.

Location	Original	Revised	Type
Abstract, Results	(no absolute rates)	Added 3-y MARE rates, ARR and NNT for the Delayed phenotype	Addition
Introduction, p. 4	48-word compound sentence	Split into two sentences	Clarity
Results, §3.2	“striking dissociation”	“marked dissociation”	Tone
Results, §3.5	“…fueling a pyroptotic loop that obliterates the microvasculature”	Reframed with human-validation caveat	Tone / qualifier
Figure 5 legend	“drastically suppressed”	“markedly reduced”	Tone
Methods, §2.4.1	—	Added registration-accuracy sentence	Addition
Discussion	—	Added ‘Future clinical translation and remaining technical hurdles’	Addition
Global (text/tables/legends)	“P<0.001”, “P=0.002”	“P < 0.001”, “P = 0.002” (spaced operators)	Formatting
Global (terminology)	“elderly”	“middle-aged-and-older”	Terminology

A complete tracked-changes version is provided in the revised manuscript (red font).

Supplementary Table S14.

Correlation between angiographic stenosis severity and the AI Perfusion Risk Score, overall and by phenotype (Discovery Cohort, N = 1,226).

Group	n	Spearman ρ (95% CI)	P	Partial ρ (95% CI)†	P†
Overall	1,226	0.29 (0.24–0.34)	<0.001	0.27 (0.22–0.32)	<0.001
Preserved	510	0.61 (0.55–0.66)	<0.001	0.58 (0.52–0.64)	<0.001
Delayed	435	0.42 (0.34–0.49)	<0.001	0.40 (0.32–0.47)	<0.001
Rarefied	281	0.06 (−0.04–0.16)	0.27	0.05 (−0.05–0.15)	0.34

†Partial Spearman correlation adjusted for age and baseline eGFR. Concordant patterns for the time-to-peak prolongation index and the wash-in slope (overall $\rho = 0.32$ and -0.27; both $P < 0.001$; abolished within Rarefied). Loss of correlation in the Rarefied phenotype indicates hypoperfusion independent of upstream stenosis severity.

Supplementary Table S15.

Pre-surgical metabolic and hemodynamic comparability of the murine groups (one week before surgery; mean ± SD, n = 12 per group).

Parameter (unit)	Young Sham	Young 2K1C	Aged Sham	Aged 2K1C	P*
Body weight (g)	27.8 ± 1.9	27.2 ± 2.1	32.9 ± 1.8	32.4 ± 2.1	0.51
Fasting blood glucose (mmol/L)	7.0 ± 0.8	7.2 ± 0.9	7.0 ± 0.9	6.8 ± 0.7	0.41
HbA1c (%)	4.2 ± 0.3	4.3 ± 0.3	4.5 ± 0.4	4.5 ± 0.3	0.78
Tail-cuff systolic BP (mmHg)	110 ± 6	112 ± 7	116 ± 7	118 ± 8	0.46
Tail-cuff diastolic BP (mmHg)	72 ± 5	73 ± 6	76 ± 6	77 ± 7	0.52
Total cholesterol (mmol/L)	2.2 ± 0.4	2.3 ± 0.4	3.5 ± 0.6	3.4 ± 0.5	0.62
Triglycerides (mmol/L)	0.9 ± 0.2	1.0 ± 0.2	1.2 ± 0.3	1.3 ± 0.4	0.55
HDL cholesterol (mmol/L)	1.4 ± 0.3	1.4 ± 0.2	1.3 ± 0.3	1.2 ± 0.3	0.49
LDL cholesterol (mmol/L)	0.6 ± 0.2	0.7 ± 0.2	1.6 ± 0.4	1.6 ± 0.4	0.71
Serum creatinine (µmol/L)	14.8 ± 2.0	15.1 ± 2.2	16.0 ± 2.4	16.2 ± 2.1	0.78
Urinary albumin/creatinine ratio (mg/g)	12.4 ± 4.1	13.1 ± 4.6	18.2 ± 5.8	19.0 ± 6.2	0.61

**P for Aged Sham vs Aged 2K1C; within-young comparison also non-significant (all $P > 0.10$, two-tailed t-tests with Bonferroni correction). The expected age effect (aged > young) is preserved for weight, BP and lipids ($P < 0.05$ between age strata). Animals with fasting glucose ≥ 11.1 mmol/L or weight beyond 2 SD were excluded a priori; none qualified. Eleven parameters as listed in Methods §2.5.1.*

Supplementary Table S16.

Pre-specified 24-month post-stenting response trajectories by AI-identified hemodynamic phenotype (all stented patients, n = 395).

Phenotype	Stented n	Strong Responder, n (%)	Modest Responder, n (%)	Non-Responder, n (%)	Progressor, n (%)
Cluster 1 (Preserved)	142	22 (15.5)	64 (45.1)	38 (26.8)	18 (12.7)
Cluster 2 (Delayed)	168	62 (36.9)	58 (34.5)	32 (19.0)	16 (9.5)
Cluster 3 (Rarefied)	85	6 (7.1)	14 (16.5)	24 (28.2)	41 (48.2)
**Pooled**	**395**	**90 (22.8)**	**136 (34.4)**	**94 (23.8)**	**75 (19.0)**

Favorable response (Strong + Modest Responder): Preserved 60.6%, Delayed 71.4%, Rarefied 23.6% (Pearson $\chi^2 = 89.4$, df = 6, $P < 0.001$).

Response categories were defined a priori (Methods §2.7.1): Strong Responder, no MARE and 24-month eGFR change ≥ −5% from baseline; Modest Responder, no MARE and eGFR decline 5–20%; Non-Responder, no MARE and eGFR decline > 20%; Progressor, MARE within 24 months or eGFR decline > 50%. Favorable Response combines Strong and Modest Responders. Percentages are row proportions within each phenotype. Distributions were compared by Pearson χ^2 with Wilson confidence intervals.
